# Safety of Supplementation of Omega-3 Polyunsaturated Fatty Acids: A Systematic Review and Meta-Analysis of Randomized Controlled Trials

**DOI:** 10.1016/j.advnut.2023.08.003

**Published:** 2023-08-09

**Authors:** Jane Pei-Chen Chang, Ping-Tao Tseng, Bing-Syuan Zeng, Cheng-Ho Chang, Huanxing Su, Po-Han Chou, Kuan-Pin Su

**Affiliations:** 1 College of Medicine, China Medical University, Taichung, Taiwan; 2 Department of Psychiatry & Mind-Body Interface Laboratory (MBI-Lab), China Medical University Hospital, Taichung, Taiwan; 3 Institute of Biomedical Sciences, National Sun Yat-sen University, Kaohsiung, Taiwan; 4 WinShine Clinics in Specialty of Psychiatry, Kaohsiung, Taiwan; 5 Department of Psychology, College of Medical and Health Science, Asia University, Taichung, Taiwan; 6 Prospect Clinic for Otorhinolaryngology & Neurology, Kaohsiung, Taiwan; 7 Institute of Precision Medicine, National Sun Yat-sen University, Kaohsiung City, Taiwan; 8 Department of Internal Medicine, E-Da Cancer Hospital, I-Shou University, Kaohsiung, Taiwan; 9 Department of Psychiatry, Kaohsiung Veterans General Hospital, Kaohsiung, Taiwan; 10 Department of Food Science, National Pingtung University of Science and Technology, Pingtung, Taiwan; 11 State Key Laboratory of Quality Research in Chinese Medicine, Institute of Chinese Medical Sciences, University of Macau, Macao, China; 12 Department of Psychiatry, China Medical University Hsinchu Hospital, China Medical University, Taichung, Taiwan; 13 An-Nan Hospital, China Medical University, Tainan, Taiwan

**Keywords:** adverse effect, docosahexaenoic acid (DHA), eicosapentaenoid acid (EPA), ω-3 polyunsaturated fatty acids (PUFAs), prescription ω-3 PUFA products (RxOME3FAs), tolerability

## Abstract

There is no comprehensive review of the evidence to support omega-3 polyunsaturated fatty acids (PUFAs) as a relatively safe and tolerable intervention. This study aimed to provide a meta-analytic and comprehensive review on the adverse effects of all kinds of ω-3 PUFA supplementation reported in randomized controlled trials (RCTs) in human subjects. A systematic review of RCTs published between 1987 and 2023 was carried out based on searches of 8 electronic databases. All RCTs that compared the adverse effects of ω-3 PUFAs containing eicosapentaenoic acid, docosahexaenoic acid, or both compared with controls (a placebo or a standard treatment) were included. The primary outcome was the adverse effects related to ω-3 PUFA prescription. A total of 90 RCTs showed that the ω-3 PUFA group, when compared with the placebo, had significantly higher odds of occurrence of diarrhea (odds ratio [OR] = 1.257, *P* = 0.010), dysgeusia (OR = 3.478, *P* < 0.001), and bleeding tendency (OR = 1.260, *P* = 0.025) but lower rates of back pain (OR = 0.727, *P* < 0.001). The subgroup analysis showed that the prescription ω-3 PUFA products (RxOME3FAs) had higher ω-3 PUFA dosages than generic ω-3 PUFAs (OME3FAs) (3056.38 ± 1113.28 mg/d compared with 2315.92 ± 1725.61 mg/d), and studies on RxOME3FAs performed more standard assessments than OME3FAs on adverse effects (63% compared with 36%). There was no report of definite ω-3 PUFA-related serious adverse events. The subjects taking ω-3 PUFAs were at higher odds of experiencing adverse effects; hence, comprehensive assessments of the adverse effects may help to detect minor/subtle adverse effects associated with ω-3 PUFAs.

This study was registered at PROSPERO as CRD42023401169.

**Statement of Significance**

Both prescription and generic omega-3 polyunsaturated fatty acids (PUFAs) might be associated with higher rates of some types of adverse effects. Moreover, although prescription ω-3 PUFAs appear to have more adverse effects than generic ω-3 PUFAs, this difference may be due to the higher dosage and the systematic evaluation of adverse effects commonly performed in the trials using prescription ω-3 PUFAs.

## Introduction

Omega-3 polyunsaturated fatty acids (PUFAs), especially EPA and DHA, are essential for humans and a promising natural remedy for many disorders, as demonstrated in many studies [1]. Several studies have demonstrated the benefits of ω-3 PUFAs in treating mental disorders, including depression, dementia, and attention deficit hyperactivity disorder [[2], [3], [4], [5], [6], [7]] as well as medical disorders, such as cardiovascular disorders, hypertriglyceridemia, and nonalcoholic fatty liver [8,9].

The number of clinical studies using ω-3 PUFAs for treatment has doubled in the past 2 decades, and more trials used higher than the minimum daily recommended nutritional supplementation dosage (200–500 mg DHA + EPA), which may also vary by country [10,11]. In addition, some of the clinical studies focused on patients comorbid with several medical disorders, e.g., patients with type 2 diabetes mellitus (T2DM) or dyslipidemia and pregnant mothers at risk of depression [12]. The safety and tolerability of ω-3 PUFAs have been continuously discussed as its strengths as a potential nutritional therapeutic agent [7,13]. However, we should be aware that the advantages of safety might be overestimated because there have been no extensive investigations of safety in the trials testing ω-3 PUFA supplementation, especially ω-3 PUFAs with high-quality content (e.g., prescription ω-3 PUFAs [RxOME3FAs]).

In this study, we performed a systematic review followed by a meta-analysis on the subjective and objective tolerability profiles of ω-3 PUFA supplementation in all the clinical trials in humans. Moreover, we analyzed the adverse effects by considering the quality of ω-3 PUFAs in the clinical trials. Thus, to the best of our knowledge, this is the first large-scale meta-analysis investigating the potential adverse events (AEs) and laboratory abnormalities associated with ω-3 PUFA supplementation, focusing on RxOME3FAs compared with generic ω-3 PUFAs (OME3FAs) in randomized controlled trials (RCTs).

## Methods

### Protocol

**FIGURE 1:**
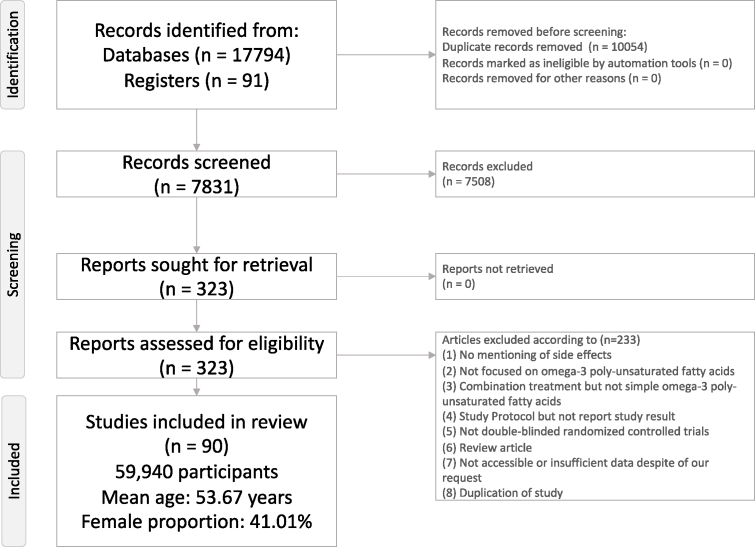
Flow chart of current meta-analysis.

The present study followed the PRISMA guidelines [14] (Figure 1). The current meta-analysis fulfilled the certification requirements of the Institutional Review Board of the Kaohsiung Veterans General Hospital (approval # VGHKS17-EM10-01) and has been registered in PROSPERO (CRD42023401169).

### Statistical analysis

In the present study, the meta-analytic procedures consisted of the following 2 parts: *1*) dichotomous outcomes: the differences in treatment-related AEs by system organ class and by MedDRA Preferred Terms or commonly used terms, and *2*) continuous outcomes: the differences in treatment-related AEs on laboratory measurements.

The meta-analytic procedures were performed in the Comprehensive Meta-Analysis software, version 3 (CMA ver. 3.0; Biostat). A 2-tailed *P* value of <0.05 was considered to indicate statistical significance. Please refer to the Supplementary Material for the details of the methods and statistical analysis.

## Results

### Studies retrieved and characteristics

The full search strategy is illustrated in Figure 1. A total of 331 articles entered the full-text screening stage, and 103 of them were excluded based on our exclusion criteria (Supplementary Tables 1-5). Furthermore, 90 studies were excluded because we could not extract specific AE data from the articles or obtain the unpublished data from the corresponding authors (Supplementary Table 4). Finally, the remaining 90 articles were included in the present meta-analysis.

### Description and characteristics of the included studies

The detailed characteristics of the included studies are listed in Supplementary Table 1. In brief, the included studies originated from Argentina, Australia, Brazil, Canada, China, Denmark, France, Germany, India, Iran, Israel, Italy, Japan, Malaysia, Mexico, Netherlands, New Zealand, Norway, Poland, Romania, Russian Federation, South Africa, Sweden Taiwan, UK, Ukraine, and United States. All trials were published between 1987 and 2023. A total of 90 articles were included with a total of 59,940 participants in the ω-3 PUFA treatment group (mean age = 53.67 ± 11.81 y, mean female proportion = 41.01 ± 23.83 %, mean ω-3 PUFA treatment duration = 41.52 ± 63.06 wk, mean EPA dosage = 1559.20 ± 939 mg/d, mean DHA dosage = 889.05 ± 569.45 mg/d, mean EPA/DHA proportion = 1.38 ± 0.62) and 58,490 subjects in the control group (mean age = 53.58 ± 12.65 y, mean female proportion = 43.38 ± 24.31%). The length of the follow-ups in the trials ranged from 1 wk to 385 wk with a median duration of 18.0 (39 interquartile range) weeks.

Among the 90 included RCTs, the characteristics of study participants included 27 studies in participants with dyslipidemia, 22 in participants with cardiovascular diseases, 8 studies in healthy participants, 7 studies in participants with diabetes, 3 studies in pregnant women, 4 studies in patients on hemodialysis, 2 studies in patients with hypertension, 2 studies in patients with Crohn’s disease, 2 studies in patients with nonalcoholic liver diseases, and 1 study for each in participants with the following clinical conditions: age-related cognitive decline, colorectal adenomas, cognitive decline, cyclosporine-treated liver transplant, depression, dry eye, dysglycemia, end-stage renal diseases, Huntington disease, metabolic syndrome, migraine, women with obesity, psoriasis, renal transplant, stroke, and ultrahigh risk of psychotic disorder. Among the 90 included studies, 46 used RxOME3FAs (US Food and Drug Administration-approved ω-3 PUFAs for clinical application; we have updated 25 new studies since our last report [15]), and 44 used generic ω-3 PUFAs (OME3FAs).

### Methodologic quality of included studies

The details of the methodologic quality assessment of included studies are provided in Supplementary Table 5. The median of the Jadad scores of the included 90 studies was 4, with a 25%–75% interquartile range 3–5.

### Review of serious AEs

Among the 90 included studies, there were no reports of serious AEs.

### Main results of the meta-analysis of prevalence rate of AEs in participants taking ω-3 PUFAs and controls: dichotomous items

**FIGURE 2:**
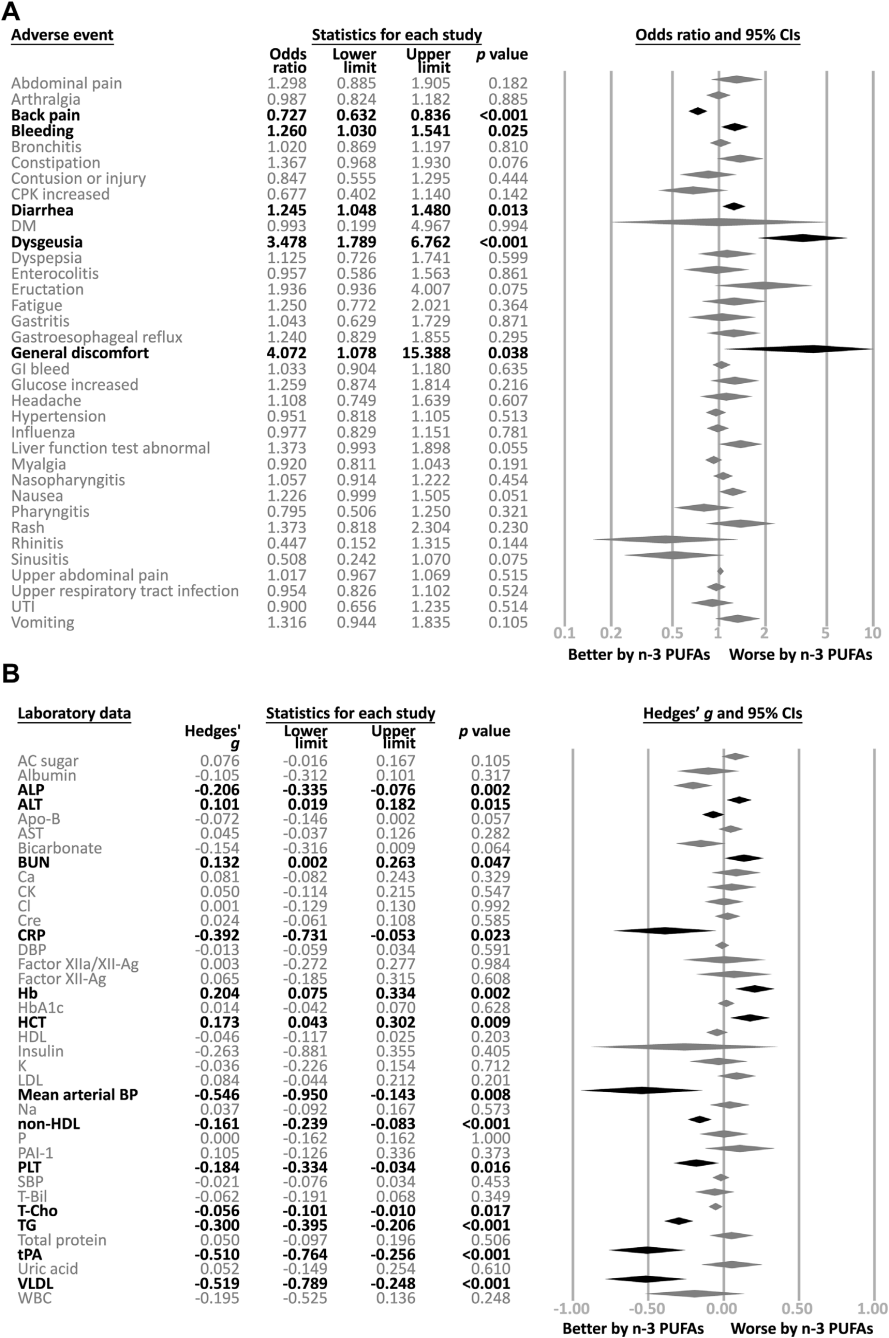
Forest plot of current meta-analysis of adverse events. (A) categorical outcome, (B) continuous outcome. Abbreviations: AC sugar, fasting sugar; Ag, antigen; ALP, alkaline phosphatase; ALT, alanine transaminase; Apo-B, apolipoprotein-B; AST, aspartate transaminase; BP, blood pressure; BUN, blood urea nitrogen; CI, confidence interval; CPK, creatine phosphokinase; Cre, creatinine; CRP, C-reactive protein; DBP, diastolic blood pressure; DM, diabetes mellitus; GI, gastrointestinal; Hb, hemoglobin; HbA1c, glycated hemoglobin; Hct, hematocrit; HDL, high-density lipoprotein; LDL, low-density lipoprotein; MA, meta-analysis; Plt, platelet; PUFA, polyunsaturated fatty acid; SBP, systolic blood pressure; T-Bil, total bilirubin; T-Cho, total cholesterol; TG, triglyceride; tPA, tissue-type plasminogen activator; UTI, urinary tract infection; VLDL, very low-density lipoprotein; WBC, white blood cell.

The results of the meta-analysis regarding the rate of AEs in participants taking ω-3 PUFAs and those taking the placebo were shown in Tables 1-2 and Figure 2. In brief, we found significantly higher rates of “diarrhea” (odds ratio [OR]: 1.245; 95% confidence interval [CI]: 1.048, 1.480; *P* = 0.013), “dysgeusia” (OR: 3.478; 95% CI: 1.789, 6.762; *P* < 0.001), and “bleeding tendency” (OR: 1.260; 95% CI: 1.030, 1.541; *P* = 0.025), but a lower rate of “back pain” (OR: 0.727; 95% CI: 0.632, 0.836; *P* < 0.001) in participants receiving ω-3 PUFA supplementation than those receiving placebo (Table 1 and Figure 2A).

**TABLE 1:** Meta-analysis of prevalence rate of adverse events

Treatment-emergent adverse events		Meta-analysis result				Heterogeneity				Publication bias		
SOC	Adverse events	Data	OR	95% CI	*P*	Q value	df	*I*^*2*^ (%)	*p*	Significance	Adj. ES	95% CI
Gastrointestinal disorders	Abdominal pain	14	1.298	0.8885, 1.905	0.182	22.982	13	43.435	0.042	n/s	—	—
	Constipation	12	1.367	0.968, 1.930	0.076	24.017	11	54.199	0.013	Sig.	1.246	0.884, 1.756
	Diarrhea	43	1.257	1.056, 1.496	0.010	86.21	42	51.282	<0.001	Sig.	1.012	0.840, 1.219
	Dysgeusia	19	3.478	1.789, 6.762	<0.001	107.148	18	83.201	<0.001	Sig.	3.264	1.706, 6.247
	Dyspepsia	11	1.125	0.726, 1.741	0.599	9.880	10	<0.001	0.451	n/s	—	—
	Eructation	17	1.936	0.936, 4.007	0.075	50.042	16	68.027	<0.001	n/s	—	—
	Gastroesophageal reflux	14	1.240	0.829, 1.855	0.295	15.920	13	18.342	0.253	n/s	—	—
	Gastrointestinal bleeding	8	1.033	0.904, 1.180	0.635	5.182	7	<0.001	0.638	n/s	—	—
	Nausea	30	1.226	0.999, 1.505	0.051	42.353	29	31.528	0.052	Sig.	1.132	0.894, 1.434
	Liver function abnormal	13	1.354	0.973, 1.882	0.072	7.882	12	<0.001	0.794	Sig.	1.354	0.973, 1.882
	Abdominal pain, upper	8	1.017	0.967, 1.069	0.515	3.752	7	<0.001	0.808	n/s	—	—
	Vomiting	16	1.316	0.944, 1.835	0.105	13.370	15	<0.001	0.574	n/s	—	—
General disorders and administration site conditions	Fatigue	7	1.250	0.772, 2.021	0.364	2.447	6	<0.001	0.874	Sig.	1.154	0.725, 1.837
Musculoskeletal and connective tissue disorders	Arthralgia	13	0.987	0.824, 1.182	0.885	13.805	12	13.075	0.313	Sig.	0.966	0.759, 1.229
	Back pain	11	0.727	0.632, 0.836	<0.001	4.982	10	<0.001	0.892	Sig.	0.725	0.631, 0.833
	CPK increased	5	0.677	0.402, 1.140	0.142	8.436	4	52.581	0.077	Sig.	0.976	0.596, 1.599
	Myalgia	12	0.920	0.811, 1.043	0.191	7.382	11	<0.001	0.767	n/s	—	—
Infections and infestations	Bronchitis	6	1.020	0.869, 1.197	0.810	1.098	5	<0.001	0.954	Sig.	1.014	0.865, 1.189
	Gastritis	11	1.043	0.629, 1.729	0.871	8.306	10	<0.001	0.599	n/s	—	—
	Enterocolitis	8	0.957	0.586, 1.563	0.861	3.436	7	<0.001	0.842	Sig.	0.914	0.563, 1.483
	Influenza	9	0.977	0.829, 1.151	0.781	4.154	8	<0.001	0.843	Sig.	0.968	0.822, 1.140
	Nasopharyngitis	17	1.057	0.890, 1.255	0.529	17.342	16	7.737	0.364	n/s	—	—
	Pharyngitis	5	0.756	0.479, 1.193	0.229	2.877	4	<0.001	0.579	Sig.	0.852	0.555, 1.308
	Rhinitis	3	0.447	0.152, 1.315	0.144	0.348	2	<0.001	0.840	n/s	—	—
	Sinusitis	6	0.508	0.242, 1.070	0.075	5.807	5	13.891	0.325	Sig.	0.875	0.359, 2.133
	Upper respiratory tract infection	10	0.967	0.835, 1.119	0.653	7.114	9	<0.001	0.622	Sig.	0.974	0.842, 1.127
	Urinary tract infection	6	0.975	0.826, 1.151	0.765	2.789	5	<0.001	0.733	Sig.	0.971	0.823, 1.146
Injury, poisoning, and procedural complications	Contusion	6	0.847	0.555, 1.295	0.444	2.309	5	<0.001	0.805	n/s	—	—
Nervous system and psychiatric disorders	Headache	12	1.108	0.749, 1.639	0.607	11.219	11	1.951	0.425	n/s	—	—
Skin and subcutaneous tissue disorders	Rash	18	1.373	0.818, 2.304	0.230	50.580	17	66.390	0.001	n/s	—	—
Cardiovascular disorders	Hypertension	7	0.945	0.813, 1.100	0.467	4.393	6	<0.001	0.624	Sig.	0.940	0.809, 1.093
Coagulopathy	Bleeding tendency	22	1.260	1.030, 1.541	0.025	33.197	21	36.740	0.044	n/s	—	—
Metabolism	Diabetes mellitus	3	0.993	0.199, 4.967	0.994	1.865	2	<0.001	0.394	n/s	—	—
	Blood sugar increased	8	1.259	0.874, 1.814	0.216	6.000	7	<0.001	0.540	Sig.	1.219	0.851, 1.748

Abbreviations: adj. ES, adjusted effect size; CI, confidence interval; CPK, creatine phosphokinase; df, degree of freedom; n/s, not significant; OR, odds ratio; Sig., significant; SOC, System Organ Class.

We then performed subgroup analysis on the types of ω-3 PUFAs (RxOME3FAs or OME3FAs), the ω-3 PUFA dosage (≥3000 mg/d compared with <3000 mg/d), and whether the trial performed routine standard AE evaluations (such as periodic AE assessment with scales). Subgroup analysis showed that the RxOME3FAs, a dosage ≥3000 mg/d, and routine standard AE evaluations were associated with a lower OR of back pain; OME3FAs, a dosage <3000 mg/d, and routine standard AE evaluations were associated with a higher OR of diarrhea; RxOME3FAs were associated with a higher OR of bleeding tendency; both RxOME3FAs and OME3FAs were associated with a higher OR of dysgeusia; and OME3FAs were associated with a higher OR for dysgeusia than RxOME3FAs (Table 3, Supplementary Material, Supplementary Table 2).

**TABLE 3:** Differences (of treatment-emergent adverse effects) between RxOME3FAs and OME3FAs

Treatment-emergent adverse effects		RxOME3FAs	OME3FAs	Differences via interaction test
Adverse events (categorical)	Dysgeusia	OR = 1.789 (1.084–2.951), *P* = 0.023	OR = 6.009 (3.332–10.837), *P <* 0.001	*P =* 0.002
	Skin rashes	OR = 1.398 (0.783–2.497), *P* = 0.257	OR = 1.397 (0.452–4.313), *P =* 0.561	*P*=0.999
	Constipation	OR = 1.335 (0.911–1.957), *P* = 0.139	OR = 1.583 (0.661–3.790), *P =* 0.303	*P*=0.726
	Abdominal pain	OR = 1.136 (0.846–1.525), *P* = 0.397	OR = 1.438 (0.666–3.104), *P*=0.355	*P*=0.575
	Arthralgia	OR = 1.004 (0.818–1.231), *P* = 0.972	Insufficient data	n/a
	Back pain	OR = 0.714 (0.619–0.823), *P* < 0.001	Insufficient data	n/a
	Bleeding tendency	OR = 1.366 (1.025–1.820), *P*=0.033	OR = 1.133 (0.832–1.544), *P*=0.428	*P*=0.386
	Bronchitis	OR = 1.017 (0.866–1.194), *P*=0.838	Insufficient data	n/a
	Contusion	OR = 0.840 (0.388–1.820), *P*=0.658	Insufficient data	n/a
	CPK increase	OR = 0.677 (0.402–1.140), *P*=0.142	Insufficient data	n/a
	Diarrhea	OR = 1.044 (0.881–1.238), *P*=0.618	OR = 1.716 (1.155–2.551), *P*=0.008	*P*=0.024
	Dyspepsia	OR = 1.154 (0.664–2.006), *P*=0.612	OR = 1.208 (0.242–6.025), *P*=0.818	*P*=0.958
	Enterocolitis	OR = 0.957 (0.586–1.563), *P*=0.861	Insufficient data	n/a
	Eructation	OR = 1.408 (0.750–2.643), *P*=0.287	OR = 3.446 (1.292–9.193), *P*=0.013	*P*=0.132
	Liver function abnormal	OR = 1.342 (0.957–1.882), *P*=0.088	OR = 1.601 (0.363–7.056), *P*=0.534	*P*=0.820
	Fatigue	OR = 1.281 (0.774–2.121), *P*=0.335	Insufficient data	n/a
	Gastritis	OR = 0.979 (0.364–2.633), *P*=0.967	OR = 1.050 (0.547–2.016), *P*=0.883	*P*=0.908
	GERD	OR = 1.074 (0.841–1.372), *P*=0.568	OR = 1.219 (0.496–3.001), *P*=0.666	*P*=0.790
	GI bleeding	OR = 1.027 (0.898–1.174), *P*=0.696	OR = 3.074 (0.480–19.675), *P*=0.236	*P*=0.248
	Headache	OR = 0.916 (0.549–1.528), *P*=0.737	OR = 1.594 (0.854–2.976), *P*=0.143	*P*=0.179
	Hypertension	OR = 0.927 (0.792–1.085), *P*=0.344	Insufficient data	n/a
	Influenza	OR = 0.977 (0.829–1.151), *P*=0.781	Insufficient data	n/a
	Myalgia	OR = 0.902 (0.785–1.036), *P*=0.145	OR = 1.004 (0.747–1.349), *P*=0.979	*P*=0.520
	Nasopharyngitis	OR = 1.063 (0.899–1.257), *P*=0.477	Insufficient data	n/a
	Nausea	OR = 1.112 (0.923–1.338), *P*=0.264	OR = 1.737 (0.901–3.349), *P*=0.099	*P*=0.200
	Pharyngitis	OR = 0.756 (0.479–1.139), *P*=0.229	Insufficient data	n/a
	Rhinitis	Insufficient data	Insufficient data	n/a
	Sinusitis	OR = 0.217 (0.081–0.583), *P*=0.002	Insufficient data	n/a
	Upper abdominal pain	OR = 1.016 (0.966–1.068), *P*=0.545	OR = 1.622 (0.585–4.500), *P*=0.352	*P*=0.369
	URI	OR = 0.981 (0.845–1.138), *P*=0.797	Insufficient data	n/a
	UTI	OR = 0.953 (0.800–1.136), *P*=0.594	OR = 1.190 (0.705–2.009), *P*=0.514	*P*=0.431
	Vomiting	OR = 1.298 (0.770–2.185), *P*=0.327	OR = 1.503 (0.826–2.736), *P*=0.182	*P*=0.716
Effects on lipid profiles	TG	Hedges’ *g* = −0.380 (−0.496 to −0.264), *P*<0.001	Hedges’ *g* = −0.218 (−0.412 to −0.023), *P*=0.029	*P*=0.161
	HDL	Hedges’ *g* = −0.072 (−0.215 to 0.072), *P*=0.330	Hedges’ *g* = −0.035 (−0.058 to −0.012), *P*=0.003	*P*=0.621
	VLDL	Hedges’ *g* = −0.519 (−0.789 to −0.248), *P*<0.001	Insufficient data	n/a
	T−Cho	Hedges’ *g* = −0.108 (−0.211 to −0.006), *P*=0.038	Hedges’ *g* = −0.015 (−0.089 to 0.060), *P*=0.696	*P*=0.148
	Non-HDL	Hedges’ *g* = −0.229 (−0.372 to −0.086), *P*=0.002	Insufficient data	n/a
	LDL	Hedges’ *g* = 0.237 (−0.036 to 0.510), *P*=0.089	Hedges’ *g* = −0.053 (−0.234 to 0.128), *P*=0.566	*P*=0.083
Adverse effects on nonlipid profiles	AC blood sugar	Hedges’ *g* = 0.113 (0.029–0.198), *P*=0.008	Hedges’ *g* = 0.029 (−0.222 to 0.280), *P*=0.821	*P*=0.532
	ALT	Hedges’ *g* =0.099 (0.014–0.184), *P*=0.022	Insufficient data	n/a
	Hb	Hedges’ *g* =0.204 (0.075–0.334), *P*=0.002	Insufficient data	n/a
	Hct	Hedges’ *g* =0.173 (0.043–0.302), *P*=0.009	Insufficient data	n/a
	ALP	Hedges’ *g* = −0.206 (−0.335 to −0.076), *P*=0.002	Insufficient data	n/a
	Plt	Hedges’ *g* = −0.184 (−0.334 to −0.034), *P*=0.016	Insufficient data	n/a
	Apo-B	Hedges’ *g* = −0.129 (−0.277 to 0.019), *P*=0.088	Hedges’ *g* = −0.024 (−0.055 to 0.008), *P*=0.140	*P*=0.173
	AST	Hedges’ *g* = −0.173 (−0.426 to 0.081), *P*=0.182	Insufficient data	n/a
	BUN	Hedges’ *g* = 0.132 (0.002–0.263), *P*=0.047	Insufficient data	n/a
	Ca	Hedges’ *g* = 0.081 (−0.082 to 0.243), *P*=0.329	Insufficient data	n/a
	CK	Hedges’ *g* = 0.050 (−0.114 to 0.215), *P*=0.547	Insufficient data	n/a
	Cl	Hedges’ *g* = 0.001 (−0.129 to 0.130), *P*=0.992	Insufficient data	n/a
	Cre	Hedges’ *g* = 0.024 (−0.061 to 0.108), *P*=0.585	Insufficient data	n/a
	CRP	Hedges’ *g* = −0.003 (−0.214 to 0.209), *P*=0.979	Hedges’ *g* = −0.803 (−1.631 to 0.025), *P*=0.057	*P*=0.067
	DBP	Hedges’ *g* = −0.302 (−0.779 to 0.174), *P*=0.213	Hedges’ *g* = −0.005 (−0.039 to 0.030), *P*=0.795	*P*=0.222
	HbA1c	Hedges’ *g* = 0.064 (−0.079 to 0.207), *P =* 0.380	Hedges’ *g* = −0.007 (−0.069 to 0.082), *P =* 0.864	*P =* 0.486
	Insulin	Insufficient data	Insufficient data	n/a
	K	Hedges’ *g* = −0.036 (−0.226 to 0.154), *P =* 0.712	Insufficient data	n/a
	Mean arterial BP	Hedges’ *g* = −0.546 (−0.950 to −0.143), *P =* 0.008	Insufficient data	n/a
	Na	Hedges’ *g* = 0.037 (−0.092 to 0.167), *P =* 0.573	Insufficient data	n/a
	P	Hedges’ *g* = 0.000 (−0.162 to 0.162), *P =* 0.999	Insufficient data	n/a
	Platelet	Hedges’ *g* = −0.184 (−0.334 to −0.034), *P =* 0.016	Insufficient data	n/a
	SBP	Hedges’ *g* = −0.485 (−1.128 to 0.159), *P =* 0.140	Hedges’ *g* = −0.011 (−0.075 to 0.052), *P =* 0.723	*P =* 0.151
	T-Bil	Hedges’ *g* = −0.062 (−0.191 to 0.068), *P =* 0.349	Insufficient data	n/a
	Total protein	Hedges’ *g* = 0.050 (−0.097 to 0.196), *P =* 0.506	Insufficient data	n/a
	Uric acid	Hedges’ *g* = 0.052 (−0.149 to 0.254), *P =* 0.610	Insufficient data	n/a
	WBC	Hedges’ *g* = 0.014 (−0.118 to 0.147), *P =* 0.833	Insufficient data	n/a
	Bicarbonate	Hedges’ *g* = −0.154 (−0.316 to 0.009), *P =* 0.064	Insufficient data	n/a
	Albumin	Hedges’ *g* = −0.105 (−0.312 to 0.101), *P =* 0.317	Insufficient data	n/a
	tPA	Insufficient data	Hedges’ *g* = −0.510 (−0.764 to −0.256), *P <* 0.001	n/a

Abbreviation: AC sugar, fasting sugar; adj. ES, adjusted effect size; ALP, alkaline phosphatase; ALT, alanine transaminase; Apo-B, apolipoprotein-B; AST, aspartate transaminase; BP, blood pressure; BUN, blood urea nitrogen; CI, confidence interval; CPK, creatine phosphokinase; Cre, creatinine; CRP, C-reactive protein; DBP, diastolic blood pressure; GERD, gastroesophageal reflux disorder; GI, gastrointestinal; Hb, hemoglobin; Hct, hematocrit; HDL, high-density lipoprotein; LDL, low-density lipoprotein; n/a: not available; OME3FA: generic ω-3 fatty acid; OR, odds ratio; Plt, platelet; RxOME3FA, prescription ω-3 fatty acid; SBP, systolic blood pressure; T-Bil, total bilirubin; T-Cho, total cholesterol; TG, triglyceride; tPA, tissue-type plasminogen activator; URI, upper respiratory tract infection; UTI, urinary tract infection; VLDL, very low-density lipoprotein; WBC, white blood cell.

### Main results of the meta-analysis of differences of AEs in laboratory data from participants taking ω-3 PUFAs and controls: continuous variables

The detailed results of our meta-analysis of AEs measured by laboratory data in the participants taking ω-3 PUFAs and those taking placebo are listed in Table 2 and Figure 2B.

**TABLE 2:** Meta-analysis of laboratory effects

Adverse effect		Meta-analysis result				Heterogeneity				Publication bias		
		Data	Hedges’ *g*	95% CI	*P*	Q value	df	*I*^*2*^ (%)	*P*	Significance	Adj. ES	95% CI
Lipid profile	HDL	35	−0.051	−0.135, 0.033	0.233	323.658	34	89.495	<0.001	n/s	—	—
	LDL	34	0.087	−0.066, 0.240	0.263	1031.151	33	96.800	<0.001	n/s	—	—
	Non-HDL	10	−0.155	−0.245, −0.066	0.001	35.456	9	74.617	<0.001	Sig.	−0.070	−0.167, 0.027
	T-Cho	38	−0.052	−0.106, 0.001	0.055	79.857	37	53.667	<0.001	n/s	—	—
	TG	39	−0.294	−0.378, −0.210	<0.001	251.526	38	84.892	<0.001	Sig.	−0.158	−0.247, -0.069
	VLDL	7	−0.519	-0.789, -0.248	<0.001	28.533	6	78.971	<0.001	n/s	—	—
Nonlipid	AC sugar	17	0.061	−0.035, 0.157	0.211	32.227	16	50.352	0.009	n/s	—	—
	Albumin	3	−0.105	−0.312, 0.101	0.317	3.231	2	38.107	0.199	n/s	—	—
	ALP	5	−0.206	−0.335, −0.076	0.002	3.897	4	<0.001	0.420	Sig.	−0.232	−0.351, −0.112
	ALT	9	0.099	0.015, 0.183	0.021	1.701	8	<0.001	0.989	n/s	—	—
	Apo-B	8	−0.072	−0.146, 0.002	0.057	19.682	7	64.434	0.006	Sig.	-0.086	−0.159, −0.014
	AST	9	0.036	−0.048, 0.120	0.401	4.262	8	<0.001	0.833	Sig.	0.068	−0.009, 0.145
	Bicarbonate	3	−0.154	−0.316, 0.009	0.064	0.617	2	<0.001	0.734	n/s	—	—
	BUN	5	0.132	0.002, 0.263	0.047	4.060	4	1.488	0.398	n/s	—	—
	Ca	3	0.081	−0.082, 0.243	0.329	1.892	2	<0.001	0.388	n/s	—	—
	Cl	5	0.001	−0.129, 0.130	0.992	0.371	4	<0.001	0.985	Sig.	0.010	−0.107, 0.128
	CPK	5	0.050	−0.114, 0.215	0.547	10.937	4	63.429	0.027	Sig.	−0.068	−0.253, 0.116
	Cre	8	0.024	−0.061, 0.108	0.585	5.948	7	<0.001	0.546	Sig.	0.064	−0.008, 0.136
	CRP	10	−0.392	−0.731, −0.053	0.023	121.729	9	92.606	<0.001	Sig.	−0.829	−1.277, −0.382
	DBP	9	−0.016	−0.067, 0.036	0.545	12.381	8	35.384	0.135	n/s	—	—
	Factor XIIa/XII-Ag	4	0.003	−0.272, 0.277	0.984	3.597	3	16.597	0.308	n/s	—	—
	Factor XII-Ag	4	0.065	−0.185, 0.315	0.608	0.834	3	<0.001	0.841	n/s	—	—
	Hb	6	0.204	0.075, 0.334	0.002	2.921	5	<0.001	0.712	n/s	—	—
	HbA1c	9	0.019	−0.040, 0.079	0.522	15.366	8	47.936	0.052	n/s	—	—
	Hct	6	0.173	0.043, 0.302	0.009	1.810	5	<0.001	0.875	Sig.	0.116	0.006, 0.226
	Insulin	3	−0.263	−0.881, 0.355	0.405	8.964	2	77.689	0.011	Sig.	0.171	−0.367, 0.709
	K	5	−0.036	−0.226, 0.154	0.712	8.471	4	52.781	0.076	Sig.	−0.129	−0.312, 0.053
	Mean arterial BP	3	−0.546	−0.950, −0.143	0.008	2.260	2	11.517	0.323	n/s	—	—
	Na	5	0.037	−0.092, 0.167	0.573	3.749	4	<0.001	0.441	n/s	—	—
	P	3	0.000	−0.162, 0.162	1.000	<0.001	2	<0.001	1.000	n/s	—	—
	Plasminogen activator inhibitor-1	5	0.105	−0.126, 0.336	0.373	0.501	4	<0.001	0.973	Sig.	0.127	−0.082, 0.336
	Plt	8	−0.184	−0.334, −0.034	0.016	10.214	7	31.463	0.177	Sig.	−0.199	−0.343, −0.055
	SBP	10	−0.020	−0.076, 0.037	0.497	27.628	9	67.424	0.001	Sig.	0.010	−0.058, 0.079
	T-Bil	5	−0.062	−0.191, 0.068	0.349	1.803	4	<0.001	0.772	Sig.	−0.078	−0.196, 0.039
	Total protein	5	0.050	−0.097, 0.196	0.506	5.112	4	21.756	0.276	n/s	—	—
	tPA	4	−0.510	−0.764, −0.256	<0.001	0.001	3	<0.001	0.999	Sig.	−0.512	−0.738, −0.285
	Uric acid	3	0.052	−0.149, 0.254	0.610	0.453	2	<0.001	0.797	Sig.	0.068	−0.122, 0.259
	WBC	6	−0.195	−0.525, 0.136	0.248	31.512	5	84.133	<0.001	Sig.	−0.358	−0.694, −0.022

Abbreviations: AC sugar, fasting sugar; adj. ES, adjusted effect size; Ag, antigen; ALP, alkaline phosphatase; ALT, alanine transaminase; Apo-B, apolipoprotein-B; AST, aspartate transaminase; BP, blood pressure; BUN, blood urea nitrogen; CI, confidence interval; CPK, creatine phosphokinase; Cre, creatinine; CRP, C-reactive protein; DBP, diastolic blood pressure; df, degree of freedom; Hb, hemoglobin; HbA1c, glycated hemoglobin; Hct, hematocrit; HDL, high-density lipoprotein; LDL, low-density lipoprotein; n/s, not significant; Plt, platelet; SBP, systolic blood pressure; Sig., significant; T-Bil, total bilirubin; T-Cho, total cholesterol; TG, triglyceride; tPA, tissue-type plasminogen activator; VLDL, very low-density lipoprotein; WBC, white blood cell.

In brief, at the end of the study period, the ω-3 PUFA group had a significantly lower level of non-HDL (Hedges’ *g* = −0.161), VLDL (Hedges’ *g* = −0.519), total cholesterol (T-Cho, Hedges’ *g* = −0.056), and triglyceride (TG, Hedges’ *g* = −0.300). Subjects receiving ω-3 PUFAs also had a significantly lower level of alkaline phosphatase (ALP, Hedges’ *g* = −0.206), tissue plasminogen activator (Hedges’ *g* = −0.510), hemoglobin (Hedges’ *g* = 0.204), hematocrit (Hedges’ *g* = 0.173), mean arterial pressure (Hedges’ *g* = −0.546), and C-reactive protein (CRP, Hedges’ *g* = −0.392). On the other hand, subjects taking ω-3 PUFAs had a significantly higher level of alanine transaminase (ALT, Hedges’ *g* = 0.101), platelets (Hedges’ *g* = −0.184), and blood urea nitrogen (BUN, Hedges’ *g* = 0.132). We then performed subgroup analysis to examine if our results would be affected by the types (RxOME3FAs compared with OME3FAs), the dosages of ω-3 PUFAs (≥3000 mg/d compared with <3000 mg/d), and the application of routine standard AE evaluation. We found that the ω-3 PUFAs were associated with a better effect on triglycerides in all subgroups, regardless of ω-3 PUFA types (RxOME3FAs compared with OME3FAs), ω-3 PUFA dosage (≥3000 mg/d compared with <3000 mg/d), and routine standard AE evaluations (compared with no routine standard AE evaluations). We found that RxOME3FAs, an ω-3 PUFA dosage of ≥3000 mg/d, and routine standard AE evaluations were associated with a better effect on non-HDL and VLDL in the treatment group, whereas RxOME3FAs, an ω-3 PUFA dosage of ≥3000 mg/d, and routine standard AE evaluations were associated with a worse effect on fasting glucose in the treatment group. RxOME3FAs and routine standard AE evaluations were associated with a better effect of ω-3 PUFAs on T-Cho, whereas trials using OME3FAs reported a better effect of ω-3 PUFAs on HDL (Table 3, Supplementary Material, Supplementary Table 3).

## Discussion

To our knowledge, this is the first large-scale meta-analysis focusing on the safety and tolerability of all types of ω-3 PUFA supplementation. Trials using RxOME3FA and OME3FA supplementation were included. Among the 90 included studies (with a median duration of follow-up of 18 wk), there was no report of definite ω-3 PUFA-related serious AEs. Our study showed that patients taking ω-3 PUFAs experienced some AEs. Moreover, when we compared studies using RxOME3FAs and OME3FAs, we found that studies using RxOME3FAs used a higher mean dosage of ω-3 PUFAs (3056.38 ± 1113.28 mg/d compared with 2315.92 ± 1725.61 mg/d, *P* = 0.009) and performed more routine standard AE evaluations (63% compared with 36%, *P* = 0.011). In terms of nonserious adverse effects, our findings are comprised of 3 parts: *1*) treatment-related AEs; *2*) treatment-related abnormal laboratory changes of lipid profiles; *3*) treatment-related abnormal nonlipid laboratory changes.

### Treatment-related AEs (dichotomous items)

Our study showed that subjects receiving ω-3 PUFAs had a higher rate of diarrhea and dysgeusia than those receiving placebo. Moreover, the occurrence of bleeding tendency was associated with RxOME3FAs but not associated with the dosages of ω-3 PUFAs. Our finding of non–dose-dependent bleeding tendency is consistent with the GISSI-Prevenzione study, which showed that daily intake of dosage higher than 3000 mg of ω-3 PUFAs was not associated with reports of higher rates of bleeding; moreover, >80% of patients were taking aspirin concomitantly in the study [16]. In another study conducted by Wachira et al. [17], ω-3 PUFAs did not increase the risk of clinically significant bleeding when used alone or in combination with anticoagulation agents.

### Treatment-related laboratory changes of lipid profiles (continuous variables)

Table 2 shows that ω-3 PUFAs had significantly beneficial effects on non-HDL, T-Cho, TG, and VLDL when compared with the placebo group. ω-3 PUFAs increase the “good cholesterol” and lower the “bad cholesterol” by having an impact on reverse cholesterol transport. Reverse cholesterol transport is the process in which excess peripheral cholesterol is transported to the liver for hepatobiliary excretion, thus inhibiting foam cell formation and the development of atherosclerosis [18]. Both DHA and EPA have been suggested to exert atheroprotective functions by promoting intracellular catabolism of apolipoprotein-B-100–containing lipoproteins, suppressing hepatic apolipoprotein-B production, stimulating plasma TG clearance via lipoprotein lipase, increasing the VLDL to LDL conversion rate, reducing LDL synthesis, and attenuating postprandial lipemia [19,20].

Of note, there was a greater improvement in the serum levels of TG and T-Cho in patients receiving the RxOME3FAs but not in patients receiving OME3FAs (Table 3). This finding further supports the notion that RxOME3FAs have more beneficial effects on the lipid profile than OME3FAs. Moreover, regardless of the ω-3 PUFA dosage and ω-3 types (RxOME3FAs compared with OME3FAs), ω-3 PUFAs lowered serum levels of TG. ω-3 PUFAs were also associated with lower TG and T-Cho levels in the trials with routine standard AE evaluations but a higher T-Cho level in the trials lacking standard AE evaluations. Our results suggested that the lipid-lowering effect of ω-3 PUFAs can be well demonstrated only if ω-3 PUFAs are RxOME3FAs and only when the trials performed routine and standard AE evaluations.

### Treatment-related nonlipid laboratory changes (continuous variables)

The results showed that participants in the ω-3 PUFA group, when compared to those in the placebo group, had lower serum levels of ALP and CRP but a higher level of ALT and BUN (Table 2). The CRP-lowering effects in the ω-3 PUFA group are consistent with those reported in previous studies in which serum CRP levels are inversely related to blood levels of DHA and EPA [21], and duration of 6-mo supplementation with ω-3 PUFAs was associated with reduced CRP serum levels [22]. Moreover, our findings further supported the notion that ω-3 PUFAs provide anti-inflammatory effects in inflammation-associated disorders through the mechanism counteracting the actions of ω-6 PUFAs [3,23]. Our finding of elevated ALT levels in participants taking ω-3 PUFAs is in accordance with previous studies showing the beneficial effects of ω-3 PUFAs on γ-glutamyl transferase but not on other liver function measurements, such as ALT and aspartate aminotransferase [24,25].

Surprisingly, the subgroup analysis of our findings showed studies that used RxOME3FAs and performed routine standard AE evaluations were associated with elevated fasting glucose and a higher level of glycated hemoglobin (HbA1c, routine standard AE evaluations only). This is inconsistent with a previous study that found ω-3 PUFAs were able to significantly reduce the risk of T2DM [26] but is consistent with the findings of a meta-analysis showing no beneficial effects of ω-3 PUFAs on glucose control in patients with T2DM [27]. One of the possible explanations for this inconsistency of blood glucose may be due to the short duration of some of the studies with blood glucose measurements included in our meta-analysis, which may not be reflective of the long-term effects of ω-3 PUFAs on the nonlipid laboratory measurements, including fasting glucose and HbA1c; instead, it may underestimate the beneficial effects of ω-3 PUFAs. Moreover, the heterogeneity of the study population in our meta-analysis should also be considered, as different populations may have different sensitivities to changes in blood glucose. In addition, the routine standard AE evaluations applied in over half of the RxOME3FA trials included in our meta-analysis may also help to explain our finding that subtle physiologic changes, including blood sugar levels, may be more easily detected in trials that exerted rigorous methodology than in those that did not.

### Limitations

There are several limitations in this meta-analysis. First, because the study populations in the included trials were mostly middle-aged patients with dyslipidemia, cardiovascular diseases, and T2DM, the generalizability of the abovementioned findings may be limited. The safety and tolerability profiles may be different among different populations such as the elderly, pregnant women, or patients with other comorbidities. Second, regarding the concern about the bleeding tendency, this cannot be answered by the current study because there were no adequate data either using binary (bleeding events) or continuous (bleeding time) outcomes in this meta-analysis. There is considerable debate as to the relative merits of using RCT data as opposed to observational data in systematic reviews of AEs. In theory, well-conducted RCTs yield unbiased estimates of treatments and AEs. However, in RCTs, the characteristics of study participants are highly selected for research purpose, and the study sample size is well designed to reach statistical power for estimating the treatment efficacy [28]. Therefore, as a meta-analysis of RCTs, the results of the present study may not be able to identify rare or long-term AEs in real-world practice.

### Conclusion

In short, our meta-analysis showed that patients receiving all types of ω-3 PUFAs would experience higher rates of some mild AEs. Moreover, although RxOME3FAs may appear to have more AEs than OME3FAs, these differences in the occurrence rates of AEs may be due to the higher dosage and the systematic AE evaluations commonly performed in the trials using RxOME3FAs. In short, ω-3 PUFAs are not without AE; however, applying routine and standard AE evaluations in trials using RxOME3FAs may help to provide patients with complex medical comorbidities with a safe and tolerable treatment option.

## Author contributions

The authors‘ responsibilities were as follows – PTT: had full access to all the data in the study and takes responsibility for the integrity of the data and the accuracy of the data analysis; KPS: concept and design of the study; JPC, PTT, BSZ, CHC: acquisition, analysis, and interpretation of data. JPC, PTT, BSZ: drafting of the manuscript. CHC, HXS, PHC, KPS: critical revision of the manuscript for important intellectual content; PTT, BSZ: statistical analysis; CHC, KPC: administrative, technical, and material support; KPS: supervision; and all authors: read and approved the final version of the manuscript.

## Conflict of interest

The authors report no conflicts of interest.

## Funding

The authors of this work were supported by the following grants: MOST 109-2320-B-038-057-MY3, 110-2321-B-006-004, 110-2811-B-039-507, 110-2320-B-039-048-MY2,110-2320-B-039-047-MY3, 110-2813-C-039-327-B, 110-2314-B-039-029-MY3, 111-2321-B-006-008, and NSTC 111-2314-B-039-041-MY3 from the National Science and Technology Council, Taiwan; ANHRF 109-31, 109-40, 110-13, 110-26, 110-44, 110-45, 111-27, 111-28, 111-47, 111-48, and 111-52 from An-Nan Hospital, China Medical University, Tainan, Taiwan; CMRC-CMA-2 from Higher Education Sprout Project by the Ministry of Education (MOE), Taiwan; CMU 110-AWARD-02, 110-N-17, 1110-SR-73 from the China Medical University, Taichung, Taiwan; and DMR-106-101, 106-227, 109-102, 109-244, 110-124, 111-245, 112-097, 112-086, 112-109, 112-232 and DMR-HHC-109-11, HHC-109-12, HHC-110-10, and HHC-111-8 from the China Medical University Hospital, Taichung, Taiwan.; and Grant VGHKS107-075 from Kaohsiung Veterans General Hospital, Kaohsiung, Taiwan.

## Appendix A Supplementary data

Mutimedia component1 Mutimedia component1
