# Supplementary material for: Safety of Supplementation of Omega-3 Polyunsaturated Fatty Acids: A Systematic Review and Meta-Analysis of Randomized Controlled Trials
Source: Adv Nutr. 2023 Aug 9;14(6):1326–36. doi: 10.1016/j.advnut.2023.08.003 (PMC10721469; doi:10.1016/j.advnut.2023.08.003)
Supplement: Mutimedia component1 [file mmc1.docx]

**Supplementary Material**

This supplementary material has been provided by the authors to give readers additional information about their work.

**1. Supplementary Method**

**2. Supplementary Results**

**Supplementary Table 1 Characteristics of included studies**

**Supplementary Table 2: Summary of meta-regression of dichotomous outcomes**

**Supplementary Table 3: Summary of meta-regression of continuous outcomes**

**Supplementary Table 4: excluded studies and reasons**

**Supplementary Table 5: Jadad scores of recruited studies**

**1. Supplementary Method**

## 1.1 Literature Search and Screening

The current meta-analysis had been registered in PROSPERO (CRD42023401169). To identify eligible studies, we searched electronic databases of PubMed, Embase, ProQuest, ScienceDirect, Cochrane Library, ClinicalKey, Web of Science, and ClinicalTrials.gov to date of February 20^th^, 2023. The keyword applied in current meta-analysis were as followed "omega-3-acid ethyl ester" OR "omega-3-acid ethyl ester 90" OR "omega-3-acid ethyl ester A" OR “omega-3-carboxylic acid” OR “omega-3 PUFAs triglyceride form” OR “icosapent ethyl” OR “ethyl icosapentate” OR “lovaza” OR “omacor” OR “lotriga” OR “epanova” OR “omtryg” OR “vascepa” OR “epadel”. To expand the potentially eligible study as possible, we did not set any limitation of language. Furthermore, to make hand search of potentially eligible articles, we refer to the references list in the review articles in this area.(1-4)

At the initial stage, two authors (JP Chang and PT Tseng) independently screened the titles and abstracts of the search results to determine whether the studies were potentially eligible for the full-text screen. In case of disagreement on eligibility, we reached a third author (KP Su) to reach an agreement through consensus.

## 1.2 Inclusion and Exclusion Criteria

At the stage of the full-text screen, all the articles should have to meet the following inclusion criteria to be included: (1) randomized controlled trials (RCTs) that compared the adverse effect of omega-3 PUFAs, containing eicosapentaenoic acid (EPA), docosahexaenoic acid (DHA) or both, versus controls, either in forms of a placebo or a standard treatment; (2) human trials. To include as many eligible studies as possible, we did not set a limit on the diseases in articles. Studies would be excluded in case of exclusion criteria: (1) not human clinical studies; (2) non-RCT designs; and (3) topics not related to the adverse effects by omega-3 PUFAs supplementation.

## 1.3 Methodological Quality Appraisal

The quality of each included study was evaluated by the Jadad scale.(5) Jadad consisted of three dimensions of questions, including the quality of randomization, blindness, and cohort follow-up. The total scores ranged from zero (the worst) to five (the best).

## 1.4 Data Extraction and Correspondence with the Authors

Using a predetermined list of data forms of interest, two review authors (JP Chang and PT Tseng) independently evaluated the selected studies for review. The data extraction form included a description of the types of participants, the type, dosage and duration of marketed prescription omega-3 PUFAs (RxOME3FAs), as well as the adverse events (AEs), serious adverse events (SAEs), and laboratory measurements for each of the reviewed studies. When needed, an email addressed to the corresponding author was sent requesting unpublished data. SAEs refer to serious adverse events resulting in death, life-threatening experience, inpatient hospitalization, persistent or significant disability or incapacity, congenital anomaly or birth defect, or a condition that requires further intervention to prevent one of the consequences mentioned above.(6)

## 1.5 Heterogeneity, Publication Bias, and Sensitivity Test

We investigated heterogeneity among the recruited studies with Q statistics and the corresponding *p* values.(7) The *I^2^* statistics indicated the proportion of variation among the included studies.(8) We investigated the potential publication bias within the recruited studies via funnel plot(9) or Egger’s regression.(10) When there was a significance of publication bias, we performed Duval and Tweedie’s trim-and-fill test to adjust the ESs for potential publication bias.(11) Furthermore, to investigate the potential confounding effect by any outlier within the recruited studies, we used a sensitivity test with one study removal method to detect the potential outliers. In brief, we removed one study at a time and re-analyzed the result of the meta-analysis to see if there was any change in the results of the meta-analysis. If the results of the meta-analysis would change, then, this study might be the outlier or has larger sample sizes.(12)

## 1.6 Meta-regression and Subgroup Meta-analysis

In addition, we used meta-regression to investigate the possible confounding effects of clinical variables, such as age, female gender and treatment duration, using the unrestricted maximum likelihood method in the situation of at least five datasets. Furthermore, another aim of the current study was to compare the potential differences of adverse effects by the different quality of omega-3 products (i.e., the RxOME3FAs or general omega-3 PUFAs supplement (OME3FAs), we arranged a subgroup meta-analysis to compare the different adverse effect between RxOME3FAs and OME3Fas. Furthermore, when there was evidence of different adverse effects between RxOME3Fas and OME3Fas supplements in a specific adverse effect, we further made subgroups based upon (1) the omega-3 PUFA dosage of at least 3000 mg/day or less than 3000 mg/day or (2) whether the study was designed with a standard evaluation of adverse effect during the study. All of the subgroup meta-analysis procedures were performed when at least three articles were included.(13) Furthermore, to find the potential differences of estimates between subgroups, we also arranged interaction tests and the corresponding *p*-value(14) according to manuals of CMA ver. 3.0.

## 1.7 Statistical Analysis

Due to anticipated heterogeneity, all the effect measures were analyzed using a random-effects model rather than a fixed-effects meta-analysis because random-effects modelling is more stringent in terms of inclusion of among-study variance.(15) Under the preliminary assumption that the units of measurement of target laboratory data are heterogeneous among the recruited studies, we chose Hedges’ *g* and 95% confidence intervals (CIs) to combine the effect sizes (ESs) for continuous variables, according to the manual of the Comprehensive Meta-Analysis ver. 3.0 software. We defined the clinical meanings of our effect sizes direction as below. A Hedges’ *g* greater than 0 indicated a significantly worse effect by supplementation of omega-3 PUFAs in the situation of continuous outcomes; in the case of dichotomous outcomes, the summary of effect sizes was defined as the odds ratio (OR), which indicated that an OR higher than 1 suggested a significantly more adverse effect by supplementation of omega-3 PUFAs.

**References list in the end of supplementary material**

**2. Supplementary Results**

A total of 90 articles were included in the present meta-analysis(16-104) (See Supplementary Table 1).

## 2.1 Meta-analysis of Prevalence Rate of Adverse Events in Participants with Omega-3 PUFAs and Those in Controls: Dichotomous Variables

### 2.1.1 Sensitivity Test

The main results of the meta-analysis did not change after the removal of any one of the included studies except for the situations listed below. The prevalence rate of back pain changed into “statistically insignificant” after the removal of the dataset of Bhatt, D.L..(20) There was a “significantly” higher prevalence of skin rash in omega-3 PUFAs treatment after removal of the dataset of Manson, J.E. (2019).(56) There was a “significantly” higher prevalence of constipation in omega-3 PUFAs treatment after the removal of the dataset of Manson, J.E. (2019).(56) The prevalence rate of bleeding tendency changed to “statistical insignificance” after the removal of some of the datasets. The prevalence rate of diarrhea changed to “statistically insignificant” after the removal of some of the datasets. There was a “significantly” lower prevalence of sinusitis in omega-3 PUFAs treatment after the removal of some of the datasets. There was a “significantly” higher prevalence of liver function abnormality in omega-3 PUFAs treatment after the removal of the dataset of Kowey, P. R., 2010.(51) There was a “significantly” higher prevalence of CPK elevation in omega-3 PUFAs treatment after the removal of the dataset of Yokoyama, M. (2007).(83) There was a “significantly” higher prevalence of vomiting in omega-3 PUFAs treatment after the removal of the dataset of Carlson, S.E. (2013).(25)

### 2.1.2 Meta-regression

The main results of meta-regression were listed in Supplementary Table 2. Specifically, there were significant inverse associations between the OR of constipation and age (slope=-0.02, *k* =11, *p*=.01), of female proportion (slope=-0.02, *k*=11, *p*=.001), and of omega-3 PUFAs treatment dosage (slope=0.001, *k*=11, *p*=.001), significant inverse associations between the OR of abdominal pain and female proportion (slope=-0.01, *k*=12, *p*=.02), significant inverse associations between the OR of nausea and treatment duration (slope=-0.001,*k* =25, *p*=.02), and significant inverse associations between the OR of dysgeusia and age (slope=-0.06, *k*=16, *p*=.001) and treatment duration (slope=-0.005, *k*=13, *p*=.001). In addition, there were significant positive associations between the OR of abdominal pain and age (slope=0.03, *k*=12, *p*=.001), significant positive associations between the OR of constipation and omega-3 PUFAs treatment dosage (slope=0.001, *k*=11, *p*=.001), significant positive associations between the OR of gastritis and treatment duration (slope=0.04, *k*=6, *p*=.04), and significant positive associations between the OR of sinusitis and female proportion (slop =0.04, *k*=6, *p*=.03).

### 2.1.3 Subgroup Analysis

#### 2.1.3.1 RxOME3FAs versus OME3FAs

Both the RxOME3FAs and the OME3FAs subgroups had a significantly higher OR of dysgeusia than the placebo; furthermore, the OR of the general OME3FAs subgroup was significantly higher than that of RxOME3FAs subgroup (OR=6.009, 3.332 to 10.837, *p*<0.001; OR=1.789, 1.084 to 2.951, *p*=0.023, respectively; *p*=0.002 between these two ORs). The subgroup analysis showed that only the RxOME3FAs had a lower OR of back pain than the placebo (OR=0.714, 0.619 to 0.823,  *p*<0.001), while there was insufficient data to perform the subgroup meta-analysis for the OME3FAs subgroup. The subgroup analysis showed that only the RxOME3FAs had a higher OR of bleeding tendency than placebo (OR=1.366, 1.025 to 1.820,  *p*=0.033) but not in the general OME3FAs subgroup. Similarly, the subgroup meta-analysis showed that RxOME3FAs had a lower OR of sinusitis than placebo (OR=0.22, 0.08 to 0.58, *p*=.002) but the OME3FAs subgroup only had two studies with data. On the other hand, only OME3FAs had a higher OR of diarrhea (OR=1.716, 1.155 to 2.551, *p*=0.008) and eructation (OR = 3.446, 1.292 to 9.193, *p*=0.013) than placebo, but the not RxOME3Fas subgroup, Table 3.

#### 2.1.3.2 Different dosage

The subgroup analysis further showed that omega-3 PUFAs had a lower OR of back pain than placebo in the at least 3000 mg/day (OR=0.71, 95% CIs=0.62 to 0.82, *p*<0.001) subgroup analysis vs the less than 3000 mg/day (OR=0.89, 95% CIs=0.55 to 1.43, *p*=.62) subgroup analysis; there was no significant difference between these two ORs via interaction test (*p*=.40). There was no significant difference in the OR of bleeding tendency regardless of the dosage of at least or less than 3000mg/day subgroup analyses when omega-3 PUFAs was compared to placebo (OR=1.247, 0.991 to 1.570, *p*=0.060 vs OR=1.336, 0.969 to 1.842, *p* =0.077); there was no significant difference between these two ORs via interaction test (*p*=0.731). There was only a significantly higher OR of diarrhea with omega-3 PUFAs when compared to placebo in the less than 3000 mg/day (OR=1.333, 95% CIs=1.049 to 1.694, *p*=0.019) subgroup analysis, but not in the at least 3000 mg/day (OR=1.184, 95% CIs = 0.840 to 1.668, *p*=0.335) subgroup analysis; there was no significant difference between these two ORs via interaction test (*p*=0.577). There was only a significantly higher OR of eructation in the omega-3 PUFAs group than placebo group in the at least 3000 mg/day (OR=2.878, 1.041 to 7.959, *p*=0.042) subgroup analysis, but not in less than 3000mg/day (OR=1.613, 0.613 to 4.245, *p*=0.333) subgroup analysis; there was no significant difference between these two ORs via interaction test (*p*=0.419).

#### 2.1.3.3 Standard evaluation of adverse effect or not

There was only a significantly lower OR of back pain in the omega-3 PUFAs group when compared to the placebo group in the subgroup analysis with routine standard evaluation (OR=0.718, 0.623 to 0.827, *p*<0.001) but not in the subgroup analysis without routine standard evaluation (OR=1.123, 0.485 to 2.598, *p*=0.786); there was a significant difference between these two ORs via interaction test (*p*=0.302). There was no significant difference in the OR of bleeding tendency when comparing omega-3 PUFAs to placebo in the subgroup analysis with routine standard evaluation (OR=1.204, 0.986 to 1.470, *p*=0.068) or in the subgroup analysis without routine standard evaluation (OR=1.798, 0.963 to 3.356, *p*=0.066); there was no significant difference between these two ORs via interaction test (*p*=0.231). There was a significantly higher OR of diarrhea in omega-3 PUFAs than placebo in the subgroup analysis with routine standard evaluation (OR=1.365, 1.097 to 1.699, *p*=0.005) but not in the subgroup analysis without routine standard evaluation (OR=1.082, 0.847 to 1.383, *p*=0.527); there was no significant difference between these two ORs via interaction test (*p*=0.167). The subgroup analysis showed no significant difference in OR of eructation when comparing omega-3 PUFAs to placebo in the subgroup analysis with routine standard evaluation (OR=1.828, 0.676 to 4.947, *p*=0.235) or in the subgroup analysis without routine standard evaluation (OR=1.504, 0.751 to 3.012, *p*=0.249); there was no significant difference between these two ORs via interaction test (*p*=0.753).

## 2.2 Meta-analysis of Differences of Adverse Effects on Laboratory Data in Participants with Omega-3 PUFAs and Those in Controls: Continuous Variables

### 2.2.1 Sensitivity Test

The main results of the meta-analysis did not change after the removal of any of the included studies except for the situation listed below. The significant result of the meta-analysis of the differences in adverse effects on ALP, ALT, BUN, CRP, mean arterial pressure, and platelet changed to “insignificant” after the removal of some included datasets; this phenomenon might be due to the smaller sample sizes after the removal of those datasets. There were “significantly” higher levels of insulin in the omega-3 PUFAs group after removal of the dataset of Sirtori, C.R. (1997).(76) The were “significantly” higher levels of AC sugar in the omega-3 PUFAs group after removal of the dataset of Song, J. (2018).(77) There were “significantly” higher levels of HbA1c in the omega-3 PUFAs group after the removal of the dataset of Pooya, S. (2010).(65)

### 2.2.2 Meta-regression

The main results of the meta-regression were listed in Supplementary Table 3. Non-HDL and T-Cho were both inversely associated with female proportions (*k*=10, slope=-0.01, *p*=.01; *k*=29, slope=-0.01, *p*=.003, respectively). The LDL was inversely associated with omega-3 PUFAs treatment dosage (*k*=29, slope=-0.001, *p*=.04). There was also a positive association between VLDL and treatment duration (*k*=7, slope=0.03, *p*<.001). Also, Apo-B was inversely associated with female proportion (*k*=8, slope=-0.01, *p*=.01). Moreover, white blood count (WBC) was negatively associated with age (*k*=6, slope=-0.16, *p*= .004). Finally, systolic blood pressure was positively associated with age (*k*=6, slope=0.04, *p*=.003) but inversely associated with omega-3 PUFAs treatment dosage (*k*=6, slope=-0.001, *p*=.003). Otherwise, there was no significant association between other clinical variables and adverse effects on laboratory data.

### 2.2.3 Subgroup Analysis

#### 2.2.3.1 RxOME3FAs versus OME3FAs

There were inconsistent findings in some of the lipid profiles. There was a significantly better effect on TG in both the RxOME3FAs subgroup (Hedges’ *g*=−0.345, -0.456 to -0.233, *p*<0.001) and the OME3FAs subgroup (Hedges’ *g*=-0.237, -0.401 to -0.073, *p*=0.005) without significant difference between these two subgroups via interaction test (*p*=0.285). Similarly, we also found that RxOME3FAs significantly improved of T-Cho than placebo (Hedges’ *g*=-0.113, -0.204 to -0.021, *p*=0.016), but not in the OME3FAs subgroup (Hedges’ *g*=-0.027, -0.085 to 0.030, *p*=0.354), there was no significant difference between these two subgroups via interaction test (*p*=0.123). On the other hand, general OME3FAs significantly improved HDL (Hedges’ *g*=-0.038; -0.057 to -0.019, *p*<0.001) than placebo, but not the RxOME3FAs subgroup (Hedges’ *g*=-0.056; -0.188 to 0.076, *p*=0.406); there was no significant difference between these two subgroups via interaction test (*p*=0.790). Finally, there was a significantly worse effect on fasting sugar in the RxOME3FAs than placebo (Hedges’ *g*=0.125, 0.045 to 0.206, *p*=0.002), but a similar finding was not observed in the OME3FAs subgroup (Hedges’ *g*=0.017, -0.222 to 0.257, *p*=0.887); there was no significant difference of estimated ESs between these two subgroups via interaction test (*p*=0.403). There was a significantly improved non-HDL in the omega-3 PUFAs when compared to placebo in the subgroups of RxOME3FAs (Hedges’ *g*=-0.228, -0.358 to -0.098, *p*=0.001) but not in the subgroups of general OME3FAs (Hedges’ *g*=-0.095, -0.237 to 0.047, *p*=0.189); there was no significant difference of estimated ESs between these two subgroups (*p*=0.176) via interaction test. Finally, there was significantly improved VLDL in the omega-3 PUFAs when compared to placebo in the subgroups of RxOME3FAs (Hedges’ *g*=-0.519, -0.789 to -0.248, *p*<0.001); the analysis in subgroups of general OME3FAs could not be performed because of insufficient data (Table 3).

#### 2.2.3.2 Different dosage

The subgroup analysis revealed that omega-3 PUFAs had a significantly better effect on TG than placebo in both the at least 3000 mg/day (Hedges’ *g*=-0.361, -0.503 to -0.218, *p*<0.001) and the less than 3000 mg/day (Hedges’ *g*=-0.146, -0.231 to -0.061, *p*=0.001) subgroup analyses; there was a significantly better effect by the subgroup with a dosage of at least 3000 mg/day than the subgroup with a dosage of less than 3000 mg/day via interaction test (*p*=0.012). The subgroup analysis found that there was a significantly better improvement of T-Cho in omega-3 PUFAs than the controls in the subgroup analysis with a dosage of at least 3000 mg/day (Hedges’ *g*=-0.102, -0.203 to -0.001, *p*=0.048) but not in subgroup analysis with a dosage of less than 3000 mg/day (Hedges’ *g*=-0.014, -0.033 to 0.005, *p* =0.149); there was no significant difference between these two estimated ESs via interaction test (*p*=0.093). Next, the subgroup analysis found that there was no significant difference in HDL between omega-3 PUFAs and controls in either the subgroup analysis with a dosage of at least 3000 mg/day (Hedges’ *g*=-0.056, -0.142 to 0.031, *p*=0.206) or the subgroup analysis with a dosage of less than 3000 mg/day (Hedges’ *g*=-0.027, -0.145 to 0.090, *p*=0.648); there was no significant difference between these two estimated ESs via interaction test (*p*=0.703). The subgroup analysis found that there was a significantly improvement of non-HDL in the omega-3 PUFAs when compared to controls in subgroup analysis with a dosage of at least 3000 mg/day (Hedges’ *g*=-0.208, -0.299 to -0.118, *p*<0.001) but not in the subgroup analysis with a dosage of less than 3000 mg/day (Hedges’ *g*=-0.026, -0.057 to 0.005, *p*=0.099); there was a significant difference between these two estimated ESs via interaction test (*p*<0.001). Finally, the VLDL was only significantly improved in the omega-3 PUFAs when compared to the placebo in subgroup analysis with a dosage of at least 3000 mg/day (Hedges’ *g*=-0.607, -0.892 to -0.323, *p*<0.001); the subgroup analysis with a dosage of less than 3000 mg/day could not be performed because of insufficient data. There was a significant worse effect on fasting sugar in the omega-3 PUFAs than placebo in the subgroup analysis with a dosage of at least 3000 mg/day (Hedges’ *g*=0.168, 0.057 to 0.279, *p*=0.003) but not in the subgroup analysis with a dosage of less than 3000 mg/day (Hedges’ *g*=0.010, -0.116 to 0.135, *p*=0.878); there was no significant difference between these two estimated Ess via interaction test (*p*=0.064). The subgroup analysis found that there was no significant difference in HbA1c between omega-3 PUFAs and placebo in either the subgroup analysis with a dosage of at least 3000 mg/day (Hedges’ *g*=0.018, -0.180 to 0.218, *p*=0.860) or the subgroup analysis with a dosage of less than 3000 mg/day (Hedges’ *g*=0.011, -0.053 to 0.075, *p*=0.731); there was no significant difference between these two estimated ESs via interaction test (*p*=0.950).

#### 2.2.3.3 Standard evaluation of adverse effect or not

Similarly, the subgroup analysis revealed that omega-3 PUFAs have a better effect on TG than placebo in both the subgroup with standard evaluation of adverse effect (Hedges’ *g*=-0.309, -0.420 to -0.198, *p*<0.001) and in the subgroup without standard evaluation of adverse effect (Hedges’ *g*=-0.299, -0.579 to -0.020, *p*=0.036); there was no significant difference between these two estimated ESs via interaction test (*p*=0.951). The subgroup analysis found that there was a significantly better effect on T-Cho in the omega-3 PUFAs than placebo in the subgroup analysis with standard evaluation of adverse effect (Hedges’ *g*=-0.064, -0.107 to -0.022, *p* =0.003) but not in the subgroup without a standard evaluation of adverse effect (Hedges’ *g*=0.056, -0.100 to 0.212, *p*=0.479); . Also, there was a significant difference among these two estimated ESs via interaction test (*p*=0.144). However, in the aspect of HDL, The subgroup analysis found that there was no significantly difference of HDL between omega-3 PUFAs and placebo in the subgroup analysis with standard evaluation of adverse effect (Hedges’ *g*=-0.047, -0.128 to 0.033, *p* =0.248) or the subgroup analysis without standard evaluation of adverse effect (Hedges’ *g*=-0.039, -0.198 to 0.120, *p*=0.629); there was no significant difference between these two estimated ESs via interaction test (*p*=0.928). Finally, the non-HDL (Hedges’ *g*=−0.164, -0.243 to -0.086, *p*<0.001) and VLDL (Hedges’ *g*=-0.533, -0.821 to -0.244, *p*<0.001) were significantly improved in omega-3 PUFAs compared to placebo in the subgroups with standard evaluation of adverse effect; the subgroup analysis without standard evaluation of adverse effect could not be performed because of insufficient data. Similarly, there was a significantly worse effect on fasting sugar in the omega-3 PUFAs than placebo in the subgroup analysis with standard evaluation of adverse effect (Hedges’ *g*=0.048, 0.016 to 0.080, *p*=0.003) but not in the subgroup analysis without standard evaluation of adverse effect (Hedges’ *g*=0.019, -0.322 to 0.359, *p*=0.915); there was no significant difference between these two estimated ESs via interaction test (*p*=0.866). Finally, there was only a significantly worse effect on HbA1c in the omega-3 PUFAs than placebo in the subgroup analysis with standard evaluation of adverse effect (Hedges’ *g*=0.025, 0.002 to 0.048, *p*=0.035) but not in the subgroup analysis without standard evaluation of adverse effect (Hedges’ *g*=-0.121, -0.567 to 0.325, *p*=0.595); there was no significant differences between these two estimated ESs via interaction test (*p*=0.521).

**References list in the end of supplementary material**

**Supplementary Table 1** **Characteristics of included studies**

| Author, year | Country | Study design | Populations | | | Interventions | | | | Jadad scores |
| --- | --- | --- | --- | --- | --- | --- | --- | --- | --- | --- |
|  |  |  | Number and gender (female %) | Age (mean±SD) | Health status | Sup. Types  (Extracted data*) | Comparison | Ratio of  EPA to DHA | Duration (weeks) |  |
| ASCEND Study, 2018(16) | UK | DB-RCT | 7740 (37.4)  7740 (37.4) | 63.3±9.2  63.3±9.2 | DM | General  (1),(2),(3) | Omega-3 1000mg/d (EPA 460 mg/d + DHA 380 mg/d)  Placebo | 1.2 | 385 | 5 |
| Badalamenti, 1995(17) | Italy | DB-RCT | 14 (38.5)  13 (46.2) | 44.8±9.0  47.8±3.0 | Cyclosporine treated liver transplant | General  (3) | Omega-3 12000 mg/d (EPA 2160 mg/d + DHA 1440 mg/d)  Placebo | 1.5 | 8 | 3 |
| Ballantyne, 2012 (18) | USA | DB-RCT | 233 (39.0)  236 (39.0)  233 (38.0) | 61.1±10.0  61.8±9.4  61.1±10.1 | Dyslipidemia^#^ | RxOME3FAs  (1), (3) | Vascepa 4000 mg/d (EPA 4000 mg/d)  Vascepa 2000 mg/d (EPA 2000 mg/d)  Placebo | NA | 12 | 4 |
| Belluzzi, 1996(19) | Italy | DB-RCT | 39(51.3)  39(48.7) | 34(18-67)  39(20-65) | Crohn’s disease | General  (1) | Omega-3 2700 mg/d (EPA 1800 mg/d + DHA 900 mg/d) | 2.0 | 52 | 4 |
| Bhatt, 2019(20) | 11 Countries | DB-RCT | 4089(28.4)  4090(29.2) | 46.0(57-69)  46.0(57-69) | Dyslipidemia^#^ | RxOME3FAs (1), (2), (3) | Vascepa 4000 mg/d (EPA 4000 mg/d)  Placebo | NA | 254.8 | 5 |
| Bianconi, 2011 (21) | Italy | DB-RCT | 104 (29.5)  100 (32.6) | 69.3±8.0  69.2±7.8 | Persistent AF | General  (1) | Omega-3 2000 mg/d  Placebo | 1.2 | 24 | 5 |
| Borthwick, 1998 (22) | UK | DB-RCT | 29 (17.2)  26 (23.1) | 54.1±9.2  52.8±9.2 | Dyslipidemia^#^ | RxOME3FAs (1), (2) | Omacor 4000 mg/d (EPA 1840 mg/d + DHA 1520 mg/d)  Corn oil | 1.2 | 22 | 3 |
| Brinton, 2018 (23) | USA | DB-RCT | 74 (100.0)  72 (100.0) | 61.2±9.4  62.1±9.7 | Diabetes mellitus and Dyslipidemia^#^ | RxOME3FAs  (1),(2),(3) | Vascepa 4000 mg/d (Icosapent ethyl 4000mg/d) | NA | 12 | 3 |
| Brouwer, 2009 (24) | 26 cardiology clinical across Europe | DB-RCT | 273 (15.0)  273 (16.0) | 60.5±12.8  62.4±11.4 | Implantable cardioverter defibrillators | General  (1) | Omega-3 900 mg/d (EPA 450 mg /d+ DHA 350 mg/d)  Placebo | 1.3 | 51 (2 to 54 ) | 5 |
| Budoff (2021)(97) | USA | DB-RCT | 30(47)  37(46) | 55.6±7.7  58.3±8.6 | Coronary atherosclerosis | General  (2), (3) | Omega-3 4000mg/d (EPA 3000mg/d)  Placebo | NA | 36 | 4 |
| Carlson, 2013 (25) | USA | DB-RCT | 154 (100.0)  147 (100.0) | 25.3±4.9  24.8±4.7 | Pregnant mothers <20 weeks of gestation o birth | General  (1) | Omega-3 1500 mg/d (DHA 600mg/d)  Placebo | NA | <20 week gestation to birth | 3 |
| Carney, 2009 (26) | USA | DB-RCT | 62(35.5)  60(31.7) | 58.1±9.4  58.6±8.5 | Depression | RxOME3FAs  (1) | Lovaza 2000 mg/d (EPA 930 mg/d + DHA 750 mg/d) | 1.2 | 10 | 5 |
| Dasarathy, 2015 (27) | USA | DB-RCT | 18 (66.7)  19 (89.5) | 51.5±6.9  49.8±12.1 | DM | General  (2), (3) | Omega-3 3600 mg/d (EPA 2160 mg/d+ DHA 1400 mg/d)  Placebo | 1.5 | 48 | 5 |
| Davidson, 2007(28) | USA | DB-RCT | 122 (45.9)  132 (39.4) | 60.3±10.1  59.3±10.8 | Dyslipidemia^#^ | RxOME3FAs  (1),(2) | Simvastatin + Lovaza 4000 mg/d (EPA 1860 mg/d + DHA 1500 mg/d)  Simvastatin + vegetable oil capsules | 1.2 | 8 | 4 |
| de Ferranti, 2014(29) | USA | DB-RCT | 12 (58.0)  12 (25.0) | 13.3±2.4  14.7±2.7 | Dyslipidemia^#^ | RxOME3FAs  (2) | Lovaza 4000 mg/d (EPA 1860 mg/d + DHA 1500 mg/d)  Corn oil | 1.2 | 24 | 5 |
| Dry Eye Group, 2018(30) | USA | DB-RCT | 349 (81.4)  186 (80.6) | 58.3±13.5  57.5±12.6 | Dry Eye | General  (1) | Omega-3 3000 mg/d (EPA 2000 mg/d + DHA 1000 mg/d)  Placebo | 2.0 | 52 | 5 |
| Eide, 2019(102) | Norway | DB-RCT | 66(28.8)  66 (22.7) | 52.8±13.5  54.1±14.2 | Renal transplant recipient | RxOME3FAs  (1), (2), (3) | Omacor 2600mg (EPA 1380mg /d + DHA 1140mg/d )  Placebo | 1.2 | 44 | 5 |
| Farquharson, 2011(95) | 2011 | DB-RCT | 97(18)  97(36) | 64±10  64±11 | Receiving cardiac surgery or CABG | General  (1) | Omega-3 4600mg/d (EPA 2700mg/d +DHA 1900mg/d)  Placebo | 1.4 | 4 | 5 |
| Fayh,2018(31) | Brazil | DB-RCT | 15 (46.7)  15 (73.3) | 50.5±6.1  50.7±6.7 | DM | General  (2),(3) | Omega-300mg/d (EPA180mg/d + 120mg/d)  Placebo | 1.5 | 8 | 5 |
| Feagan, 2008(32) | Canada, Europe, Israel, USA | DB-RCT | 183 (51.9)  180 (58.9) | 40.5±15.2  38.2±13.1 | Crohn’s Disease | RxOME3FAs  (1) | Epanova 2000 mg/d (EPA 1000-1200 mg/d + DHA 300-500 mg/d)  Placebo | 2.4 to 3.3 | 52 | 4 |
| Finnegan, 2003(33) | UK | DB-RCT | 30 (41.9)  31 (43.3)  30 (43.3)  29 (41.4)  30 (40.0) | 53.0±2.0  54.0±2.0  52.0±2.0  54.0±2.0  55.0±2.0 | Dyslipidemia^#^ | General  (2),(3) | Omega-3 (moderate EPA + DHA group) 800 mg/d  Omega-3 (high EPA + DHA group) 1700 mg/d  Omega-3 (moderate ALA group) 4500 mg/d  Omega-3 (high ALA group) 9500 mg/d  Placebo | NA | 24 | 5 |
| Fujioka, 2006(34) | Japan | DB-RCT | 68 (57.4)  73 (58.9) | 38.8±11.6  37.0±11.5 | Healthy middle- aged men | General  (3) | Omega-3 860 mg/d (EPA 860 mg/d)  Placebo | NA | 12 | 5 |
| Geelen, 2005(35) | Netherlands | DB-RCT | 38 (28.9)  36 (52.8) | 68.0±11.0  62.0±14.0 | Frequent premature ventricular complexes | General  (1) | Omega-3 1500 mg/d (EPA700 mg /d+ DHA 500 mg/d)  Placebo | 1.4 | 14 | 4 |
| GISSI-HF Investigators 2008(105) | Italy | DB-RCT | 3494 (22.2)  3481 (22.2) | 67.0±11.0  67.0±11.0 | Heart Failure | General  (1) | Omega-3 1000 mg/d (EPA 386-340 mg/d + DHA 464-408 mg/d) | 0.8 | 202.8 | 5 |
| Goodfellow, 2000(36) | UK | DB-RCT | 15 (42.9)  15 (26.7) | 56.0±13.0  50.0±12.0 | Dyslipidemia^#^ | General  (2),(3) | Omega-3 4000 mg/d  Placebo | NS | 16 | 2 |
| Grigg, 1989(37) | Australia | DB-RCT | 52 (23.0)  56 (12.0) | 51.8±9.9  55.1±11.6 | Coronary angioplasty | RxOME3FAs  (2),(3) | Maxepa 3000mg/d (EPA 1800 mg/d+ DHA 1560 mg/d)  Placebo | 1.2 | 12 | 3 |
| Hamazaki, 1996(38) | Japan | DB-RCT | 13 (61.5)  11 (54.5) | 22 (21-30) | Healthy | General  (3) | Omega-3 3000-3600mg/d  Placebo (mainly DHA) | NA | 13 | 4 |
| Hamazaki, 1996(39) | Japan | DB-RCT | 22(77.3)  20 (65.0) | 22 (21-30) | Healthy | General  (2),(3) | Omega-3 3000-3600mg/d (EPA 201-241 mg/d + DHA 1470-1774.8mg/d)  Placebo | 0.2 to 0.1 | 12 | 4 |
| Harper, 2010(40) | USA | DB-RCT | 434 (100.0)  418 (100.0) | 28(23-32)  27(24-32) | Women with a history of prior spontaneous singleton preterm birth and a current singleton gestation | General  (1) | Omega-3 2200 mg/d (EPA 1200mg/d + DHA 800 mg/d)  Placebo | 1.5 | NA | 4 |
| Harris, 1997(41) | USA | DB-RCT | 22 (22.7)  20 (35.0) | 46.0±11.0  45.0±9.0 | Dyslipidemia^#^ | RxOME3FAs  (2),(3) | Omacor 4000 mg/d (EPA 1840 mg /d+ DHA 1520 mg/d)  Corn oil | 1.2 | 16 | 3 |
| Heydari,2016(42) | USA | DB-RCT | 180 (18.0)  178 (21.0) | 60.0±10.0  58.0±10.0 | AMI | RxOME3FAs  (1),(2) | Lovaza 4000 mg/d (EPA 1860 mg /d+ DHA 1500 mg/d)  Corn oil | 1.2 | 24 | 4 |
| Himmelfarb, 2007(43) | USA | DB-RCT | 31 (25.8)  32 (46.9) | 58.0±2.0  61.2±1.8 | Hemodialysis | General  (1),(3) | Omega-3 800 mg/d (DHA 800mg/d)  Placebo | NA | 8 | 4 |
| Holman, 2009(44) | USA | DB-RCT | 200 (45.0)  197 (44.0)  201 (39.0)  202 (42.0) | 63 (57-72)  64 (55.5-72)  64 (55-72)  65 (57-73) | Type 2 DM | RxOME3FAs  (1) | Atorvastatin + Omacor 2000 mg/d (EPA 920 mg/d + DHA 760 mg/d)  Omacor 2000 mg/d (EPA 920 mg/d + DHA 760 mg/d) + olive oil  Atorvastatin + olive oil  Olive oil+ olive oil | 1.2 | 16 | 5 |
| Hull, 2018(45) | UK | DB-RCT | 178 (22)  176(21) | 65(62-69)  65(62-69) | Colorectal adenomas | General  (1) | Omega-3 2000 mg/d (EPA 2000 mg/d) | NA | 52 | 5 |
| Irish, 2017(46) | Australia, Malaysia, New Zealand, UK | DB, RCT | 284 (37)  283 (37) | 54.1±14.0  55.6±14.5 | Hemodialysis | RxOME3FAs  (1) | Omacor 4000 mg/d (EPA 1840 mg/d + 1520 mg/d) | 1.2 | 12 | 5 |
| Jacobson, 2012(47) | USA | DB-RCT | 77 (23.0)  76 (12.0)  76 (24.0) | 51.9±10.3  53.4±9.3  53.4±8.3 | Dyslipidemia^#^ | RxOME3FAs  (1),(3) | Vascepa 4000 mg/d (EPA 4000 mg/d)  Vascepa 2000 mg/d (EPA 2000 mg/d)  Placebo | NA | 12 | 3 |
| Johansen, 1999(48) | Norway | DB-RCT | 196 (25.5)  192 (19.3) | 60.3±9.3  59.1±9.3 | PTCA | RxOME3FAs  (1),(2) | Omacor 4000 mg/d (EPA 1840 mg/d + DHA 1520 mg/d)  Corn oil | 1.2 | 24 | 3 |
| Kastelein, 2014(49) | USA | DB-RCT | 100 (20.0)  101 (21.8)  99 (28.3)  99 (22.2) | 51.1±9.8  51.2±8.8  52.9±10.9  50.8±10.6 | Dyslipidemia^#^ | RxOME3FAs  (1),(2),(3) | Epanova 2000 mg/d (EPA 1100 mg/d + DHA 400 mg/d)  Epanova 3000 mg/d (EPA 1650 mg/d + DHA 600 mg/d)  Epanova 4000 mg/d (EPA 2200 mg/d + DHA 800 mg/d)  Corn oil | 2.8 | 12 | 5 |
| Kobayashi, 2007(50) | Japan | DB-RCT | 20 (50.0)  18 (50.0) | 48.5±7.8  48.4±7.7 | Healthy patients | General  (1),(2) | Omega-3 (EPA 660 mg/d + DHA 280 mg/d)  Placebo | 2.4 | 8 | 4 |
| Kowey, 2010(51) | USA | DB-RCT | 332 (40.0)  331 (47.0) | 59.8±13.4  61.2±12.3 | sPAF | RxOME3FAs  (1) | Lovaza 4000 mg/d (EPA 1860 mg/d + DHA 1500 mg/d)  Corn oil | 1.2 | 24 | 5 |
| Kristensen, 2016(52) | Denmark | DB-RCT | 73 (55.6)  72 (60.6) | 53.2±11.4  50.7±11.5 | Psoriasis | General  (3) | Omega-3 3000 mg/d (EPA 1500 mg/d+ DHA 1500mg/d)  Placebo | 1.0 | 24 | 4 |
| Lok, 2012(92) | Canada | DB-RCT | 99 (53)  97 (47) | 62.5 (28-88)  63.4(27-87) | ESRD | General  (1), (3) | Omega-3 4000mg/d (EPA 1600mg/d + DHA 800mg/d)  Placebo | 2 | 52 | 4 |
| Macchia, 2013(96) | Italy | DB-RCT | 289(42.2)  297(48.1) | 66.3±12  65.9±10.5 | Symptomatic paroxysmal AF requiring cardioversion | General  (1) | Omeag-3 1000mg/d | NA | 52 | 5 |
| Maki, 2008(53) | USA | DB-RCT | 39 (64.0)  39 (64.0) | 60.3±10.1  59.3±10.8 | Dyslipidemia^#^ | RxOME3FAs  (1) | Simvastatin + Lovaza 4000 mg/d (EPA 1860 mg/d + DHA 1500 mg/d)  Simvastatin + vegetable oils | 1.2 | 6 | 2 |
| Maki, 2009(54) | USA | DB-RCT | 17 (64.0)  17 (64.0) | 60.1±2.7 | Dyslipidemia^#^ | RxOME3FAs  (1) | Lovaza 4000 mg/d (EPA 1860 mg/d + DHA 1500 mg/d) + simvastatin  Placebo + simvastatin | 1.2 | 12 | 2 |
| Maki, 2011(55) | USA | DB-RCT | 19 (61.3)  19 (61.3) | 56.4±2.7 | Dyslipidemia^#^ | RxOME3FAs  (1),(2) | Lovaza 4000 mg/d (EPA 1860 mg /d+ DHA 1500 mg/d)  Corn oil | 1.2 | 6 | 3 |
| Manson, 2019(56) | USA | DB-RCT | 12933 (50.6)  12938 (50.5) | 67.2±7.1  67.1±7.1 | Healthy | ROME3FAs (1) | Omacor 860 mg/d (EPA 460 mg/d + DHA 380 mg/d)  Placebo | 1.2 | 275.6 | 5 |
| Maresta, 2002(57) | Italy | DB-RCT | 125 (14.4)  132 (16.7) | 58.9±9.5  58.6±8.7 | Coronary artery stenosis | General  (1),(2) | Omega-3 5100 mg/d (EPA 2250 mg/d + DHA 1785mg/d)  Placebo | 1.3 | 24 | 4 |
| McGorry, 2017(58) | Australia | DB-RCT | 153 (49.0)  151 (59.6) | 19.4±4.8  18.9±4.3 | Ultrahigh risk for psychotic disorders | General  (1) | Omega-3 1400 mg/d (EPA 840 mg/d + DHA 560 mg/d)  Placebo | 1.5 | 24 | 5 |
| Mendez-Sanchez, 2001(59) | Mexico | DB-RCT | 12 (100.0)  11 (100.0) | 37.0±9.8  39.0±8.7 | Obese Women | General  (1) | Omega-3 11300 mg/d  Placebo | NA | 6 | 3 |
| Miller, 1988(104) | UK | DB-RCT | 48 (29.7)  38 (26.7) | 49.9(34-60)  48.0(25-66) | Dyslipidemia^#^ | RxOME3FAs  (1) | Maxepa 2850 mg/d (EPA 1700 mg/d + DHA 1150 mg/d)  Placebo | 1.5 | 12 | 3 |
| Miller, 2020(98) | USA | DB-RCT | 8  5  (*77% of total 13 participants were female) | 63±9  59±10 | Metabolic Syndrome | General  (2), (3) | Omega-3 2000mg/d (EPA 2000mg/d)  Placebo | NA | 36 | 3 |
| Mozaffarian, 2012(60) | Argentina, Italy, USA | DB-RCT | 758 (22.3)  758 (28.4) | 63.8±12.6  63.6±12.4 | Scheduled for cardiac surgery | RxOME3FAs  (1) | Omacor (Pre-operative loading of 10000 mg over 3-5 days or 8000 mg over 2 days followed post-operatively by 2000 mg/d)  Placebo | NA | Until hospital discharge or post-op day 10, whichever first | 5 |
| Naesgaard, 2017(61) | Norway | DB-RCT | 114 (18.4)  114 (18.4) | Men:  61.6±12.4  Women:  67.8±11.0 | AMI | RxOME3FAs  (2),(3) | Omacor 4000mg/d (EPA1840mg/d +DHA 1520mg/d)  Placebo | 1.2 | 52 | 4 |
| Nicholls, 2020(99) | USA | DB-RCT | 6539(35)  6539(34.9) | 62.5±9  62.5±9 | High cardiovascular risk | General  (1), (2), (3) | Omeaga-3 4000mg/d  Placebo | NA | 52 | 5 |
| Nigam, 2014(94) | Canada | DB-RCT | 153(31)  163(35) | 60±12  62±13 | Symptomatic paroxysmal or persistent AF | General  (1) | Omega-3 4000mg/d (EPA 1600mg/d + DHA 800mg/d)  Placebo | 2 | 38.7 (median) | 4 |
| Nilsen, 2001(93) | Norway | DB-RCT | 150(23.3)  150(23.3) | 64.4 (28.9-86.7)  63.6 (29.3-87.7) | AMI | RxOME3FAs  (2) | Omacor 2000mg/d (EPA 920mg/d +DHA760mg/d) | 1.2 | 78 | 4 |
| Noda, 2018(62) | Japan | DB-RCT | 6 (0.0)  6 (0.0)  6 (0.0) | 26.7±8.7  28.3±6.2  32.3±6.1 | Healthy Male | RxOME3FAs  (1),(3) | Epanova 2000mg/d  Epanova 4000 mg/d  Placebo | NA | 3 | 2 |
| Nodari, 2011(90) | Italy | DB-RCT | 67 (4.5)  66(15.1) | 61±11  64±9 | Non-Ischemic Dilated cardiomyopathy | RxOME3FAs  (2), (3) | Omacor 2000mg (EPA 920mg/d+ DHA 760mg/d)  Placebo | 0.9 to 1.5 | 96 | 5 |
| Nordoy, 2000(85) | Norway | DB-RCT | 21 (28.6)  20 (30.0) | 46.8±9.2  46.7±7.8 | Dyslipidemia^#^ | RxOME3FAs  (2),(3) | Omacor 4000 mg/d (EPA 1800 mg/d + DHA 1560 mg)/d + simvastatin  Placebo + simvastatin | 1.2 | 5 | 3 |
| Oscarsson, 2018(103) | Sweden | DB-RCT | 25(36)  26(46) | 60 (44-74)  59.5(45-72) | Dyslipidemia^#^, NAFLD | RxOME3FAs  (2), (3) | Epanova 4000mg/d (EPA 3200mg/d+ DHA 800mg/d)  Placebo | 4 | 12 | 5 |
| Olsen, 1992(63) | Denmark | DB-RCT | 266(100)  136(100) | 29.4±4.4  29.7±4.3 | Pregnant women | General  (1) | Omega-3 2000 mg/d (EPA 864 mg/d + DHA 621 mg/d) | 1.4 | NS | 5 |
| ORIGIN TRIAL, 2012 (64) | 40 countries | DB-RCT | 6281 (34.6)  6255 (35.3) | 63.5±7.8  63.6±7.9 | Dysglycemia | RxOME3FAs  (2),(3) | Omacor 1020mg/d (EPA 465mg/d + DHA 375 mg/d | 1.2 | 364 | 5 |
| Pooya, 2010(65) | Iran | DB-RCT | 40 (NA)  41 (NA) | 56.4±9.2  52.7±10.7 | Type 2 DM | General  (1),(3) | Omega 2714 mg/d (EPA 1548 mg/d +DHA 828mg/d)  Placebo | 1.9 | 8 | 3 |
| Poppitt, 2009(66) | New Zealand | DB-RCT | 51 (19.6)  51 (39.2) | 64.0±10.0  65.0±12.0 | Stroke | General  (2),(3) | Omega-3 1200 mg/d  Placebo | NA | 12 | 3 |
| Pradalier, 2001(67) | France | DB-RCT | 100 (82.0)  96 (79.0) | 39.3±11.9  39.2±10.3 | Migraine | RxOME3FAs  (1) | Maxepa 6000 mg/d (EPA 1080 mg/d + DHA 720 mg/d)  Placebo | 1.5 | 16 | 1 |
| Prisco, 1998(68) | Italy | DB-RCT | 8 (0.0)  8 (0.0) | 44.0±7.0  45.0±6.0 | Mild essential hypertensive male | RxOME3FAs  (2), (3) | Esapent 4000mg/d (EPA 2040 mg/d + DHA 1400 mg/d)  Placebo | 1.5 | 16 | 3 |
| Puri, 2005(69) | UK | DB-RCT | 67 (43.0)  68 (56.0) | 50.0±9.3  49.0±9.0 | HD | RxOME3FAs  (1) | Vascepa 2000 mg/d (EPA 2000 mg/d)  Placebo | NA | 48 | 5 |
| Qi, 2021(101) | China | DB-RCT | 122  126 |  | Dyslipidemia^3^ | RxOME3FAs  (1), (2) | Omacor 2000mg (EPA 920mg/d + DHA 760mg/d) for 4 weeks + Omacor 4000 mg (EPA 1840mg/d + DHA 1520mg/d) for 8 weeks  Placebo | 1.2 | 12 | 4 |
| Quinn, 2010(70) | USA | DB-RCT | 238(47.1)  164(59.8) | 76.0±9.3  76.0±7.8 | Alzheimer Disease | General  (1) | Omega-3 2000 mg/d (DHA 2000 mg/d) | NA | 72 | 5 |
| Radack, 1990(71) | USA | DB-RCT | 10 (30.0)  8 (25.0) | 51.0±11.5  47.0±13.9 | Dyslipidemia^#^ | General  (1) | Omega-3 2000 mg/d  Placebo | NA | 20 | 4 |
| Raitt, 2005(72) | USA | DB-RCT | 100 (14.0)  100 (14.0) | 63.0±13.0  62.0±13.0 | Implantable cardioverter defibrillator (ICD) and recent episode of sustained VT or VF | General  (1) | Omega-3 1800 mg/d (EPA 756mg/d + DHA 540mg/d)  Placebo | 1.4 | 103 (3 to 118) | 4 |
| Rantanen, 2018(89) | Denmark | DB-RCT | 56 (33.9)  56(33.9) | 64.2±14.5  60.5±13.9 | Chronic dialysis | General  (1), (2) | Omega-3 2000mg (EPA 1000mg + DHA 1000mg)  Placebo | 1 | 12 | 5 |
| Reis, 1989(73) | USA | DB-RCT | 137(27)  67(24) | 50.0±10.0  57.0±9.0 | PTCA | General  (1) | Omega-3 6000 mg/d  Placebo | NS | 24 | 4 |
| Risk And Prevention, 2013(74) | Italy | DB-RCT | 6239 (37.7)  6266 (39.4) | 63.9±9.3  64.0±9.6 | Multiple cardiovascular risk factors | General  (1), (2), (3) | Omega-3 1000 mg/d (EPA 447-510 mg/d + DHA 340-403 mg/d)  Placebo | 1.27-1.31 | 260 | 3 |
| Sandhu, 2016(75) | USA | SB-RCT | 54 (100.0)  53 (100.0)  53 (100.0) | 56.6±6.9  57.9±5.1  57.1±5.9 | PM healthy women | RxOME3FAs  (1),(2) | Lovaza 4000 mg/d (EPA 1860 mg/d + DHA 1500 mg/d)  Lovaza 4000 mg/d (EPA 1860 mg/d + DHA 1500 mg/d) + raloxifene  No treatment | 1.2 | 96 | 3 |
| Scorletti (2014)(100) | UK | DB-RCT | 51(51)  52(33) | 48.6±9.6  54±11.1 | NAFLD | RxOME3FAs  (2), (3) | Omacor 4000mg/d (EPA 1840 mg/d + DHA 1520mg/d)  Placebo | 1.2 | 66 | 2 |
| Singer, 2004(91) | Germany | DB-RCT | 33(48)  32(53) | 45±8  42±9 | Cardia arrhythmia | General  (2), (3) | Omega-3 1000mg/d (EPA 180mg +DHA 120mg) | 1.5 | 24 | 5 |
| Sirtori, 1997(76) | Italy | DB-RCT | 470 (37.4)  465 (37.8) | 58.2±9.1  58.8±9.0 | Dyslipidemia^#^ | RxOME3FAs  (2),(3) | Esapent 2580 mg/d (EPA 1530 mg/d + DHA 1050 mg/d)  placebo | 1.5 | 24 | 4 |
| Song, 2018(77) | China | DB-RCT | 51 (72.5)  52 (75.0)  51 (60.8)  47 (78.7) | 62.0±8.0  60.0±8.0  61.0±8.0  61.0±7.0 | Healthy | General  (2),(3) | Omega-3 310mg/d (EPA 186mg/d + DHA 124 mg/d)  Omega-3 630mg/d (EPA 372mg/d + DHA 248mg/d)  Omega-3 1240mg/d (744 mg/d + DHA 496 mg/d)  Placebo | 1.5 | 12 | 5 |
| Stroes, 2018(78) | USA | DB-RCT | 81 (19.8)  81 (23.5) | 50.3±10.6  50.0±20.9 | Dyslipidemia | RxOME3FAs  (1) | OM3-CA 2000 mg/d  Placebo | NA | 12 | 3 |
| Su, 2017(86) | Taiwan | DB-RCT | 82 (34.5)  84 (42.7)  87 (22.4) | 54.7±9.2  53.7±11.0  54.4±10.7 | Dyslipidemia^#^ | RxOME3FAs  (1),(2),(3) | Omacor 2000 mg/d (EPA 920 mg/d + DHA 760 mg/d)  Omacor 4000 mg/d (EPA 1840 mg/d + DHA 1520 mg/d)  Olive oil | 1.2 | 8 | 5 |
| Svensson, 2006(87) | Denmark | DB-RCT | 103 (33.0)  103 (38.0) | 66.0±11.0  68.0±12.0 | Hemodialysis | RxOME3FAs  (1) | Omacor 1700 mg/d (EPA 765 mg/d + DHA 637.5 mg/d)  Placebo | 1.2 | 80 (31 to 104) | 5 |
| Tatsuno, 2013  (ORD)(79) | Japan | DB-RCT | 206 (22.3)  210 (25.2)  195 (19.5) | 53.9±10.8  55.0±10.5  55.6±10.5 | Dyslipidemia^#^ | RxOME3FAs  (1),(2) | TAK-085 2000 mg/d (EPA 930 mg/d + DHA 750 mg/d)  TAK-085 4000 mg/d (EPA 1860 mg/d + DHA 1500 mg/d)  EPA-E 1800 mg/d (EPA 1800 mg/d) | 1.2 | 12 | 5 |
| Tatsuno, 2013  (ORL)(80) | Japan | SB-RCT | 165 (28.5)  171 (29.2)  167 (28.7) | 56.0±11.0  55.9±10.1  55.8±10.3 | Dyslipidemia^#^ | RxOME3FAs  (1) | TAK-085 2000 mg/d (EPA 930 mg/d + DHA 750 mg/d)  TAK-085 4000 mg/d (EPA 1860 mg/d + DHA 1500 mg/d)  EPA-E 1800 mg/d (EPA 1800 mg/d) | 1.2 | 52 | 2 |
| Toft, 1995(81) | Norway | DB-RCT | 38 (42.1)  40 (30.0) | 52.9±9.5  54.4±8.9 | Untreated hypertension | RxOME3FAs  (2),(3) | Omacor-3 3400 mg/d  Placebo | NA | 16 | 4 |
| van Dam, 2001(82) | Netherlands | DB-RCT | 45 (4.4)  44 (11.4) | 49.7(36-69)  50.1(33.75) | Dyslipidemia^#^ | RxOME3FAs  (1) | Omacor 4000 mg/d (EPA 1840 mg/d + DHA 1520 mg/d)  Gemifibrozil | 1.2 | 12 | 4 |
| Von Schacky, 1999(88) | Germany | DB-RCT | 111(18)  112(21.4) | 57.8±9.7  58.9±8,1 | Coronary artery disease | General  (2), (3) | Omega-3 3330mg/d for 3m + Omega-3 1650 mg/d for 21m | NA | 96 | 5 |
| Yokoyama, 2007(83) | Japan | SB-RCT | 9326 (58.0)  9319 (59.0) | 61.0±8.0  61.0±9.0 | Dyslipidemia^#^ | RxOME3FAs  (1) | EPADEL 1800 mg/d (EPA 1800 mg/d) + statin  Statin | NA | 261 | 3 |
| Yurko-Mauro, 2010(84) | USA | DB-RCT | 219 (56.0)  218 (60.0) | 70.0±9.3  70.0±8.7 | Age-related cognitive decline | General  (1) | Omega-3 900 mg/d (DHA 900 mg/d)  Placebo | NA | 24 | 4 |

Extracted data*:(1) adverse events; (2) lipid profiles; (3) non-lipid laboratory measurements

number of studies*: (1) = 60; (2) = 43; (3) =39

# Dyslipidemia patients include patients with hypercholesterolemia, hyperlipidemia, and hypertriglyceridemia

Abbreviations: Af: atrial fibrillation; AMI: acute myocardial infarction; DHA, docosahexaenoic acid; DM: diabetes mellitus; EPA, eicosapentaenoid acid; HD: Huntington disease; ICU: intensive care unit; m: month; NA: not applicable; NAFLD: non-alcoholic fatty liver diseases; NS: not specified; ORD: omega-3 fatty acids randomized double-blind study; ORL: omega-3 fatty acids randomized long-term study; mg/d: milligram per day; PTCA: percutaneous transluminal coronary angioplasty; PM: post-menopausal; RA: Rheumatoid arthritis; RCT: randomized controlled trial; DB-RCT: double-blind randomized controlled trial; SB-RCT: single-blind randomized controlled trial; RxOM3FAs, prescription omega 3 fatty acids; sPAF: symptomatic paroxysmal atrial fibrillation; UK: the United Kingdom; USA: the United States of America.

**References list in the end of supplementary material**

**Supplementary Table 2: Summary of meta-regression of dichotomous outcomes**

| Treatment-related Adverse Events | | Age | | | Female proportion | | | Omega 3 dosage | | | Tx duration | | |
| --- | --- | --- | --- | --- | --- | --- | --- | --- | --- | --- | --- | --- | --- |
| SOC | Adverse events | data | slope | *p* | data | slope | *p* | data | slope | *p* | data | slope | *p* |
| Gastrointestinal Disorders | Abdominal pain | 12 | 0.034 | 0.001 | 12 | -0.014 | 0.016 | 12 | -0.001 | 0.890 | 11 | 0.019 | 0.130 |
|  | Constipation | 11 | -0.019 | 0.011 | 11 | -0.022 | 0.001 | 11 | 0.001 | 0.001 | 1 | -0.003 | 0.188 |
|  | Diarrhea | 37 | 0.004 | 0.663 | 37 | -0.007 | 0.185 | 35 | -0.001 | 0.734 | 35 | -0.002 | 0.195 |
|  | Dysgeusia | 16 | -0.055 | 0.001 | 16 | 0.010 | 0.306 | 16 | 0.001 | 0.301 | 13 | -0.005 | 0.001 |
|  | Dyspepsia | 11 | 0.006 | 0.817 | 11 | -0.017 | 0.316 | 11 | 0.001 | 0.909 | 11 | -0.011 | 0.486 |
|  | Eructation | 16 | -0.037 | 0.244 | 16 | 0.016 | 0.122 | 16 | 0.001 | 0.203 | 15 | -0.018 | 0.110 |
|  | Gastritis | 8 | -0.018 | 0.430 | 8 | 0.006 | 0.507 | 7 | 0.001 | 0.735 | 6 | 0.042 | 0.041 |
|  | GERD | 12 | 0.006 | 0.688 | 12 | -0.001 | 0.875 | 12 | 0.001 | 0.202 | 11 | -0.002 | 0.484 |
|  | GI bleeding | 6 | -0.005 | 0.654 | 6 | -0.019 | 0.090 | 6 | 0.001 | 0.167 | 6 | -0.001 | 0.913 |
|  | Liver function abnormal | 12 | 0.027 | 0.288 | 12 | 0.008 | 0.314 | 12 | -0.001 | 0.120 | 11 | 0.002 | 0.306 |
|  | Nausea | 27 | -0.005 | 0.554 | 27 | -0.001 | 0.779 | 26 | -0.001 | 0.931 | 25 | -0.001 | 0.024 |
|  | Upper abdominal pain | 7 | -0.022 | 0.600 | 7 | -0.015 | 0.441 | 7 | 0.001 | 0.739 | 7 | -0.002 | 0.375 |
|  | Vomit | 14 | 0.005 | 0.736 | 14 | -0.002 | 0.811 | 13 | 0.001 | 0.342 | 11 | -0.006 | 0.566 |
| Musculoskeletal and Connective Tissue Disorders | Arthralgia | 13 | -0.013 | 0.107 | 13 | -0.006 | 0.305 | 13 | 0.001 | 0.658 | 13 | -0.002 | 0.071 |
|  | Back pain | 11 | -0.008 | 0.589 | 11 | 0.007 | 0.202 | 11 | -0.001 | 0.391 | 10 | 0.001 | 0.997 |
|  | CPK increased | n/d | n/d | n/d | n/d | n/d | n/d | n/d | n/d | n/d | n/d | n/d | n/d |
|  | Myalgia | 9 | -0.011 | 0.243 | 9 | -0.005 | 0.162 | 9 | 0.001 | 0.182 | 9 | -0.001 | 0.652 |
| Infections and Infestations | Bronchitis | 6 | -0.012 | 0.661 | 6 | 0.011 | 0.504 | 6 | -0.001 | 0.824 | 5 | 0.001 | 0.845 |
|  | Enterocolitis | 8 | 0.012 | 0.689 | 8 | -0.005 | 0.786 | 8 | 0.001 | 0.567 | 8 | -0.041 | 0.102 |
|  | Influenza | 9 | 0.018 | 0.596 | 9 | -0.008 | 0.543 | 9 | -0.001 | 0.960 | 9 | -0.001 | 0.720 |
|  | Nasopharyngitis | 17 | -0.002 | 0.909 | 17 | 0.002 | 0.770 | 17 | -0.001 | 0.215 | 16 | 0.001 | 0.947 |
|  | Pharyngitis | 5 | 0.689 | 0.221 | 5 | 0.031 | 0.533 | 5 | -0.001 | 0.907 | 5 | 0.027 | 0.094 |
|  | Rhinitis | n/d | n/d | n/d | n/d | n/d | n/d | n/d | n/d | n/d | n/d | n/d | n/d |
|  | Sinusitis | 6 | -0.007 | 0.853 | 6 | 0.043 | 0.027 | 6 | -0.001 | 0.767 | 5 | 0.007 | 0.829 |
|  | Upper respiratory tract infection | 10 | 0.012 | 0.349 | 10 | -0.007 | 0.272 | 10 | 0.001 | 0.300 | 9 | -0.001 | 0.842 |
|  | Urinary tract infection | 6 | 0.002 | 0.805 | 6 | 0.002 | 0.755 | 6 | -0.001 | 0.910 | n/d | n/d | n/d |
| Injury, poisoning and procedural complications | Contusion or injury | 6 | -0.002 | 0.917 | 6 | -0.003 | 0.881 | 6 | -0.001 | 0.735 | 6 | -0.007 | 0.575 |
| General Disorders and Administration Site Conditions | Fatigue | 7 | 0.049 | 0.259 | 7 | -0.013 | 0.402 | 7 | 0.001 | 0.432 | 6 | -0.023 | 0.189 |
| Coagulopathy | Bleeding tendency | 14 | 0.009 | 0.411 | 14 | -0.004 | 0.463 | 14 | 0.001 | 0.591 | 12 | -0.001 | 0.758 |
| Nervous System and psychiatric Disorders | Headache | 12 | -0.015 | 0.484 | 12 | 0.002 | 0.810 | 11 | -0.001 | 0.880 | 11 | -0.021 | 0.186 |
|  | Neuropsychiatric disease | 5 | -0.030 | 0.265 | 5 | 0.003 | 0.873 | n/d | n/d | n/d | n/d | n/d | n/d |
| Metabolism | Glucose increase | 8 | 0.050 | 0.407 | 8 | 0.011 | 0.325 | 8 | 0.001 | 0.779 | 8 | 0.001 | 0.764 |
| Cardiovascular disorders | Hypertension | 7 | -0.016 | 0.245 | 7 | 0.005 | 0.251 | 7 | -0.001 | 0.257 | 6 | 0.001 | 0.970 |
| Skin and subcutaneous tissue disorders | Skin rash | 18 | 0.009 | 0.629 | 18 | 0.013 | 0.151 | 15 | -0.001 | 0.869 | 16 | 0.002 | 0.434 |

Abbreviation: adj. ES: Adjusted effect size; CI: confidence interval; CPK: creatine phosphokinase; df: degree of freedom; n/s: not significant; OR: odds ratio; Sig.: significant; SOC: System Organ Class

**Supplementary Table 3: Summary of meta-regression of continuous outcomes**

| Treatment-related laboratory findings | | Age | | | Female proportion | | | Omega 3 dosage | | | Tx duration | | |
| --- | --- | --- | --- | --- | --- | --- | --- | --- | --- | --- | --- | --- | --- |
|  |  | data | slope | *p* | data | slope | *p* | data | slope | *p* | data | slope | *p* |
| Lipid profile | HDL | 31 | -0.004 | 0.441 | 29 | 0.001 | 0.784 | 28 | -0.001 | 0.927 | 28 | -0.001 | 0.433 |
|  | LDL | 31 | 0.003 | 0.729 | 28 | -0.001 | 0.767 | 29 | -0.001 | 0.036 | 28 | 0.001 | 0.183 |
|  | Non-HDL | 10 | -0.010 | 0.260 | 10 | -0.014 | 0.008 | 10 | -0.001 | 0.093 | 10 | 0.010 | 0.191 |
|  | T-Cho | 33 | 0.002 | 0.632 | 29 | -0.005 | 0.003 | 30 | -0.001 | 0.195 | 30 | 0.001 | 0.441 |
|  | TG | 34 | 0.007 | 0.483 | 30 | -0.005 | 0.137 | 31 | 0.001 | 0.618 | 31 | 0.001 | 0.232 |
|  | VLDL | 7 | -0.001 | 0.906 | 6 | -0.010 | 0.304 | 7 | -0.001 | 0.144 | 7 | 0.053 | <0.001 |
| Non-lipid profile | AC sugar | 15 | -0.017 | 0.192 | 12 | -0.006 | 0.016 | 17 | 0.001 | 0.034 | 12 | -0.001 | 0.060 |
|  | Albumin | n/d | n/d | n/d | n/d | n/d | n/d | n/d | n/d | n/d | n/d | n/d | n/d |
|  | ALP | 5 | 0.033 | 0.486 | 5 | 0.008 | 0.608 | 5 | -0.001 | 0.309 | 5 | -0.039 | 0.257 |
|  | ALT | 7 | 0.020 | 0.518 | 7 | 0.002 | 0.818 | 9 | 0.001 | 0.732 | 7 | -0.002 | 0.809 |
|  | Apo-B | 8 | -0.006 | 0.470 | 8 | -0.011 | 0.009 | 7 | -0.001 | 0.207 | 8 | 0.001 | 0.681 |
|  | AST | 7 | -0.005 | 0.874 | 7 | -0.003 | 0.681 | 9 | 0.001 | 0.928 | 7 | -0.005 | 0.573 |
|  | Bicarbonate | n/d | n/d | n/d | n/d | n/d | n/d | n/d | n/d | n/d | n/d | n/d | n/d |
|  | BUN | 5 | 0.018 | 0.727 | 5 | 0.008 | 0.581 | 5 | 0.001 | 0.692 | 5 | 0.003 | 0.950 |
|  | Ca | n/d | n/d | n/d | n/d | n/d | n/d | n/d | n/d | n/d | n/d | n/d | n/d |
|  | Cl | 5 | 0.006 | 0.900 | 5 | 0.002 | 0.871 | 5 | 0.001 | 0.702 | 5 | 0.001 | 0.994 |
|  | CPK | 5 | -0.001 | 0.719 | n/d | n/d | n/d | n/d | n/d | n/d | n/d | n/d | n/d |
|  | Cre | 6 | 0.004 | 0.904 | 6 | -0.001 | 0.992 | 8 | 0.001 | 0.279 | 6 | 0.001 | 0.776 |
|  | CRP | 10 | -0.019 | 0.741 | 9 | -0.006 | 0.088 | 9 | -0.001 | 0.866 | 7 | 0.003 | 0.719 |
|  | DBP | 5 | 0.017 | 0.137 | n/d | n/d | n/d | 5 | -0.001 | 0.161 | 5 | 0.001 | 0.511 |
|  | Factor XIIa/XII-Ag | n/d | n/d | n/d | n/d | n/d | n/d | n/d | n/d | n/d | n/d | n/d | n/d |
|  | Factor XII-Ag | n/d | n/d | n/d | n/d | n/d | n/d | n/d | n/d | n/d | n/d | n/d | n/d |
|  | Hb | 6 | 0.036 | 0.263 | 6 | 0.019 | 0.151 | 6 | 0.001 | 0.699 | 6 | -0.031 | 0.295 |
|  | HbA1c | 9 | -0.001 | 0.974 | 8 | -0.003 | 0.648 | 9 | -0.001 | 0.873 | 9 | -0.001 | 0.368 |
|  | Hct | 6 | 0.012 | 0.696 | 6 | 0.013 | 0.337 | 6 | 0.001 | 0.685 | 6 | -0.010 | 0.728 |
|  | Insulin | n/d | n/d | n/d | n/d | n/d | n/d | n/d | n/d | n/d | n/d | n/d | n/d |
|  | K | 5 | -0.042 | 0.550 | 5 | -0.018 | 0.376 | 5 | 0.001 | 0.112 | 5 | 0.023 | 0.680 |
|  | Mean arterial BP | n/d | n/d | n/d | n/d | n/d | n/d | n/d | n/d | n/d | n/d | n/d | n/d |
|  | Na | 5 | -0.063 | 0.153 | 5 | -0.020 | 0.125 | 5 | -0.001 | 0.661 | 5 | 0.037 | 0.277 |
|  | P | n/d | n/d | n/d | n/d | n/d | n/d | n/d | n/d | n/d | n/d | n/d | n/d |
|  | Plasminogen activator inhibitor-1 | 5 | -0.031 | 0.496 | n/d | n/d | n/d | 5 | 0.001 | 0.637 | 5 | -0.012 | 0.506 |
|  | Plt | 8 | -0.007 | 0.813 | 6 | -0.018 | 0.186 | 8 | 0.001 | 0.781 | 8 | 0.062 | 0.016 |
|  | SBP | 6 | 0.035 | 0.003 | 5 | 0.001 | 0.543 | 6 | -0.001 | 0.003 | 6 | -0.001 | 0.947 |
|  | T-Bil | 5 | -0.056 | 0.210 | 5 | -0.017 | 0.203 | 5 | 0.001 | 0.719 | 5 | 0.042 | 0.216 |
|  | Total protein | 5 | -0.068 | 0.121 | 5 | -0.021 | 0.122 | 5 | 0.001 | 0.143 | 5 | 0.056 | 0.102 |
|  | tPA | n/d | n/d | n/d | n/d | n/d | n/d | n/d | n/d | n/d | n/d | n/d | n/d |
|  | Uric acid | n/d | n/d | n/d | n/d | n/d | n/d | n/d | n/d | n/d | n/d | n/d | n/d |
|  | WBC | 6 | -0.161 | 0.004 | 6 | -0.055 | 0.170 | 6 | 0.001 | 0.051 | 6 | 0.109 | 0.408 |

Abbreviation: AC sugar: fasting sugar; adj. ES: Adjusted effect size; Ag: antigen; ALP: alkaline phosphatase; ALT: alanine transaminase; Apo-B: apo-lipoprotein-B; AST: aspartate transaminase; BP: blood pressure; BUN: blood urea nitrogen; Ca: calcium; CI: confidence interval; CPK: creatine phosphokinase; Cl: chloride; Cre: creatinine; CRP: C-reactive protein; DBP: diastolic blood pressure; df: degree of freedom; Hb: hemoglobin; Hct: hematocrit; HDL: high-density lipoprotein; K: potassium; LDL: low-density lipoprotein; Na: sodium; non-HDL: non-High-density lipoprotein; n/s: not significant; P: phosphate; Plt: platelet; SBP: systolic blood pressure; Sig.: significant; T-Bil: total bilirubin; T-Cho: total cholesterol; TG: triglyceride; tPA: tissue-type plasminogen activator; VLDL: very low-density lipoprotein; WBC: white blood cell

**Supplementary Table 4: excluded studies and reasons**

| Reason | Number | Reference |
| --- | --- | --- |
| No mentioning of side effects | 90 | (106-195) |
| Not focused on omega-3 poly-unsaturated fatty acids | 11 | (196-206) |
| Combination treatment but not simple omega-3 poly-unsaturated fatty acids | 34 | (207-240) |
| Excluded patients with adverse events | 1 | (241) |
| Study Protocol but not report study result | 3 | (242-244) |
| Not double-blinded randomized controlled trials | 49 | (245-293) |
| Review article | 3 | (294-296) |
| Not accessible or insufficient data despite of our request | 4 | (297-300) |
| Adverse event well tolerated and no any subjects reporting adverse event | 2 | (301, 302) |
| Duplication of study | 36 | (303-338) |

**References list in the end of supplementary material**

**Supplementary Table 5: Jadad scores of recruited studies**

| Author (year) | Randomization | Blindness | Cohort follow up | Total Jadad score | Country |
| --- | --- | --- | --- | --- | --- |
| Reis, 1989 | 1 | 2 | 1 | 4 | USA |
| Olsen, 1992 | 2 | 2 | 1 | 5 | Denmark |
| Miller, 1988 | 1 | 1 | 1 | 3 | UK |
| Grigg, 1989 | 2 | 1 | 0 | 3 | Australia |
| Radack, 1990 | 1 | 2 | 1 | 4 | US |
| Badalamenti, 1995 | 2 | 1 | 0 | 3 | Italy |
| Toft, 1995 | 2 | 2 | 0 | 4 | Norway |
| Belluzzi, 1996 | 1 | 2 | 1 | 4 | Italy |
| Hamazaki, 1996 | 1 | 2 | 1 | 4 | Japan |
| Hamazaki, 1996 | 1 | 2 | 1 | 4 | Japan |
| Harris, 1997 | 1 | 1 | 1 | 3 | USA |
| Sirtori, 1997 | 2 | 1 | 1 | 4 | Italy |
| Borthwick, 1998 | 1 | 1 | 1 | 3 | UK |
| Prisco, 1998 | 1 | 1 | 1 | 3 | Italy |
| Johansen, 1999 | 2 | 1 | 1 | 4 | Norway |
| Von Schacky, 1999 | 2 | 2 | 1 | 5 | Germany |
| Goodfellow, 2000 | 1 | 1 | 0 | 2 | UK |
| Nordoy, 2000 | 1 | 2 | 0 | 3 | Norway |
| Mendez-Sanchez, 2001 | 1 | 1 | 1 | 3 | Mexico |
| Nilsen, 2001 | 1 | 2 | 1 | 4 | Norway |
| Pradalier, 2001 | 1 | 0 | 0 | 1 | France |
| van Dam, 2001 | 2 | 1 | 1 | 4 | Netherlands |
| Maresta, 2002 | 1 | 2 | 1 | 4 | Italy |
| Finnegan, 2003 | 2 | 2 | 1 | 5 | UK |
| Singer, 2004 | 2 | 2 | 1 | 5 | Germany |
| Geelen, 2005 | 2 | 2 | 0 | 4 | Netherland |
| Puri, 2005 | 2 | 2 | 1 | 5 | UK |
| Raitt, 2005 | 2 | 1 | 1 | 4 | USA |
| Fujioka, 2006 | 2 | 2 | 1 | 5 | Japan |
| Svensson, 2006 | 2 | 2 | 1 | 5 | Denmark |
| Davidson, 2007 | 2 | 1 | 1 | 4 | USA |
| Himmelfarb, 2007 | 2 | 1 | 1 | 4 | USA |
| Kobayashi, 2007 | 2 | 1 | 1 | 4 | Japan |
| Yokoyama, 2007 | 2 | 0 | 1 | 3 | Japan |
| Feagan, 2008 | 2 | 1 | 1 | 4 | Canada, Europe, Isarael, USA |
| GISSI-HF Investigators, 2008 | 2 | 2 | 1 | 5 | Italy |
| Maki, 2008 | 0 | 1 | 1 | 2 | USA |
| Brouwer, 2009 | 2 | 2 | 1 | 5 | 26 cardiology clinical across Europe |
| Carney, 2009 | 2 | 2 | 1 | 5 | USA |
| Holman, 2009 | 2 | 2 | 1 | 5 | USA |
| Maki, 2009 | 0 | 1 | 1 | 2 | USA |
| Poppitt, 2009 | 2 | 1 | 0 | 3 | New Zealand |
| Harper, 2010 | 2 | 1 | 1 | 4 | USA |
| Kowey, 2010 | 2 | 2 | 1 | 5 | USA |
| Pooya, 2010 | 1 | 2 | 0 | 3 | Iran |
| Quinn, 2010 | 2 | 2 | 1 | 5 | USA |
| Yurko-Mauro, 2010 | 2 | 2 | 0 | 4 | USA |
| Bianconi, 2011 | 2 | 2 | 1 | 5 | Italy |
| Faraquharson, 2011 | 2 | 2 | 1 | 5 | Australia |
| Maki, 2011 | 1 | 1 | 1 | 3 | USA |
| Nodari, 2011 | 2 | 2 | 1 | 5 | Italy |
| Ballantyne, 2012 | 1 | 2 | 1 | 4 | USA |
| Jacobson, 2012 | 1 | 2 | 1 | 4 | USA |
| Lok, 2012 | 1 | 2 | 1 | 4 | Canada |
| Mozaffarian, 2012 | 2 | 2 | 1 | 5 | US, Italy and Argentina |
| Carlson, 2013 | 1 | 1 | 1 | 3 | USA |
| Macchia, 2013 | 1 | 2 | 1 | 4 | Italy |
| Mozurkewich, 2013 | 2 | 2 | 0 | 4 | USA |
| Risk And Prevention, 2013 | 1 | 1 | 1 | 3 | Italy |
| Tatsuno, 2013 (ORD) | 2 | 2 | 1 | 5 | Japan |
| Tatsuno, 2013 (ORL) | 1 | 0 | 1 | 2 | Japan |
| de Ferranti, 2014 | 2 | 2 | 1 | 5 | USA |
| Kastelein, 2014 | 2 | 2 | 1 | 5 | USA |
| Nigman, 2014 | 1 | 2 | 1 | 4 | Canada |
| Scorletti, 2014 | 1 | 1 | 0 | 2 | UK |
| Dasarathy, 2015 | 1 | 2 | 1 | 4 | USA |
| Heydari,2016 | 2 | 1 | 1 | 4 | USA |
| Kristensen, 2016 | 2 | 1 | 1 | 4 | Denmark |
| Sandhu, 2016 | 2 | 0 | 1 | 3 | USA |
| Irish, 2017 | 2 | 2 | 1 | 5 | Australia, Malaysia, New Zealand, UK |
| McGorry, 2017 | 2 | 2 | 1 | 5 | Australia |
| Naesgaard, 2017 | 2 | 1 | 1 | 4 | Norway |
| Su, 2017 | 2 | 2 | 1 | 5 | Taiwan |
| ASCEND, 2018 | 2 | 2 | 1 | 5 | UK |
| Brinton, 2018 | 1 | 1 | 1 | 3 | USA |
| Fayh, 2018 | 2 | 2 | 1 | 5 | Brazil |
| Hull, 2018 | 2 | 2 | 1 | 5 | UK |
| Noda, 2018 | 1 | 0 | 1 | 2 | Japan |
| Oscarsson, 2018 | 2 | 2 | 1 | 5 | Sweden |
| Rantanen, 2018 | 2 | 2 | 1 | 5 | Denmark |
| Song, 2018 | 2 | 2 | 1 | 5 | China |
| Stroes, 2018 | 1 | 1 | 1 | 3 | USA |
| Bhatt, 2019 | 2 | 2 | 1 | 5 | 11 countries |
| Eide, 2019 | 2 | 2 | 1 | 5 | Norway |
| Manson, 2019 | 2 | 2 | 1 | 5 | USA |
| Miller, 2020 | 1 | 1 | 1 | 3 | USA |
| Nicholls, 2020 | 2 | 2 | 1 | 5 | USA |
| Budoff, 2021 | 2 | 2 | 0 | 4 | USA |
| Qi, 2021 | 2 | 2 | 0 | 4 | China |

Abbreviation: UK: United Kingdom; USA: United States of America

**References of the supplement materials:**

1. Rizos EC, Ntzani EE, Bika E, Kostapanos MS, Elisaf MS. Association between omega-3 fatty acid supplementation and risk of major cardiovascular disease events: A systematic review and meta-analysis. Jama 2012;308(10):1024-1033.

2. Scorletti E, Byrne CD. Omega-3 fatty acids and non-alcoholic fatty liver disease: Evidence of efficacy and mechanism of action. Mol Aspects Med 2018;64:135-146.

3. Meyer BJ, Groot RHM. Effects of Omega-3 Long Chain Polyunsaturated Fatty Acid Supplementation on Cardiovascular Mortality: The Importance of the Dose of DHA. Nutrients 2017;9(12).

4. Agostoni C, Nobile M, Ciappolino V, Delvecchio G, Tesei A, Turolo S et al. The Role of Omega-3 Fatty Acids in Developmental Psychopathology: A Systematic Review on Early Psychosis, Autism, and ADHD. Int J Mol Sci 2017;18(12).

5. Jadad AR, Moore RA, Carroll D, Jenkinson C, Reynolds DJ, Gavaghan DJ et al. Assessing the quality of reports of randomized clinical trials: is blinding necessary? Control Clin Trials 1996;17(1):1-12.

6. Frankos VH, Street DA, O'Neill RK. FDA regulation of dietary supplements and requirements regarding adverse event reporting. Clin Pharmacol Ther 2010;87(2):239-244.

7. Higgins JP, Thompson SG. Quantifying heterogeneity in a meta-analysis. Stat Med 2002;21(11):1539-1558.

8. Borenstein M, Higgins JP, Hedges LV, Rothstein HR. Basics of meta-analysis: I(2) is not an absolute measure of heterogeneity. Res Synth Methods 2017;8(1):5-18.

9. Higgins JP, Green S. 10.4.3.1 Recommendations on testing for funnel plot asymmetry. In: Higgins JP, Green S, editors. Cochrane Handbook for Systemic Review of Interventions. 5.1.0 ed: The Cochrane Collaboration and John Wiley & Sons Ltd.; 2008.

10. Egger M, Davey Smith G, Schneider M, Minder C. Bias in meta-analysis detected by a simple, graphical test. BMJ 1997;315(7109):629-634.

11. Duval S, Tweedie R. Trim and fill: A simple funnel-plot-based method of testing and adjusting for publication bias in meta-analysis. Biometrics 2000;56(2):455-463.

12. Tobias A. Assessing the influence of a single study in meta-analysis. Stata Tech Bull 1999;47(8):15-17.

13. Davey J, Turner RM, Clarke MJ, Higgins JP. Characteristics of meta-analyses and their component studies in the Cochrane Database of Systematic Reviews: a cross-sectional, descriptive analysis. BMC Med Res Methodol 2011;11:160.

14. Altman DG, Bland JM. Interaction revisited: the difference between two estimates. Bmj 2003;326(7382):219.

15. Borenstein M, Hedges LV, Higgins JP, Rothstein HR. A basic introduction to fixed-effect and random-effects models for meta-analysis. Res Synth Methods 2010;1(2):97-111.

16. Group ASC, Bowman L, Mafham M, Wallendszus K, Stevens W, Buck G et al. Effects of n-3 Fatty Acid Supplements in Diabetes Mellitus. N Engl J Med 2018;379(16):1540-1550.

17. Badalamenti S, Salerno F, Lorenzano E, Paone G, Como G, Finazzi S et al. Renal effects of dietary supplementation with fish oil in cyclosporine-treated liver transplant recipients. Hepatology 1995;22(6):1695-1671.

18. Ballantyne CM, Bays HE, Kastelein JJ, Stein E, Isaacsohn JL, Braeckman RA et al. Efficacy and safety of eicosapentaenoic acid ethyl ester (AMR101) therapy in statin-treated patients with persistent high triglycerides (from the ANCHOR study). Am J Cardiol 2012;110(7):984-992.

19. Belluzzi A, Brignola C, Campieri M, Pera A, Boschi S, Miglioli M. Effect of an enteric-coated fish-oil preparation on relapses in Crohn's disease. N Engl J Med 1996;334(24):1557-1560.

20. Bhatt DL, Steg PG, Miller M, Brinton EA, Jacobson TA, Ketchum SB et al. Cardiovascular Risk Reduction with Icosapent Ethyl for Hypertriglyceridemia. N Engl J Med 2019;380(1):11-22.

21. Bianconi L, Calo L, Mennuni M, Santini L, Morosetti P, Azzolini P et al. n-3 polyunsaturated fatty acids for the prevention of arrhythmia recurrence after electrical cardioversion of chronic persistent atrial fibrillation: a randomized, double-blind, multicentre study. Europace 2011;13(2):174-181.

22. Borthwick L, Group UKS. The effects of an omega-3 ethyl ester concentrate on blood lipid concentrations in patients with hyperlipidaemia. Clin Drug Investig 1998;15(5):397-404.

23. Brinton EA, Ballantyne CM, Guyton JR, Philip S, Doyle RT, Jr., Juliano RA et al. Lipid Effects of Icosapent Ethyl in Women with Diabetes Mellitus and Persistent High Triglycerides on Statin Treatment: ANCHOR Trial Subanalysis. Journal of women's health (2002) 2018;27(9):1170-1176.

24. Brouwer IA, Raitt MH, Dullemeijer C, Kraemer DF, Zock PL, Morris C et al. Effect of fish oil on ventricular tachyarrhythmia in three studies in patients with implantable cardioverter defibrillators. Eur Heart J 2009;30(7):820-826.

25. Carlson SE, Colombo J, Gajewski BJ, Gustafson KM, Mundy D, Yeast J et al. DHA supplementation and pregnancy outcomes. Am J Clin Nutr 2013;97(4):808-815.

26. Carney RM, Freedland KE, Rubin EH, Rich MW, Steinmeyer BC, Harris WS. Omega-3 augmentation of sertraline in treatment of depression in patients with coronary heart disease: a randomized controlled trial. JAMA 2009;302(15):1651-1657.

27. Dasarathy S, Dasarathy J, Khiyami A, Yerian L, Hawkins C, Sargent R et al. Double-blind randomized placebo-controlled clinical trial of omega 3 fatty acids for the treatment of diabetic patients with nonalcoholic steatohepatitis. J Clin Gastroenterol 2015;49(2):137-144.

28. Davidson MH, Stein EA, Bays HE, Maki KC, Doyle RT, Shalwitz RA et al. Efficacy and tolerability of adding prescription omega-3 fatty acids 4 g/d to simvastatin 40 mg/d in hypertriglyceridemic patients: an 8-week, randomized, double-blind, placebo-controlled study. Clin Ther 2007;29(7):1354-1367.

29. de Ferranti SD, Milliren CE, Denhoff ER, Steltz SK, Selamet Tierney ES, Feldman HA et al. Using high-dose omega-3 fatty acid supplements to lower triglyceride levels in 10- to 19-year-olds. Clin Pediatr (Phila) 2014;53(5):428-438.

30. Dry Eye A, Management Study Research G, Asbell PA, Maguire MG, Pistilli M, Ying GS et al. n-3 Fatty Acid Supplementation for the Treatment of Dry Eye Disease. N Engl J Med 2018;378(18):1681-1690.

31. Fayh APT, Borges K, Cunha GS, Krause M, Rocha R, de Bittencourt PIH, Jr. et al. Effects of n-3 fatty acids and exercise on oxidative stress parameters in type 2 diabetic: a randomized clinical trial. J Int Soc Sports Nutr 2018;15:18.

32. Feagan BG, Sandborn WJ, Mittmann U, Bar-Meir S, D'Haens G, Bradette M et al. Omega-3 free fatty acids for the maintenance of remission in Crohn disease: the EPIC Randomized Controlled Trials. JAMA 2008;299(14):1690-1697.

33. Finnegan YE, Howarth D, Minihane AM, Kew S, Miller GJ, Calder PC et al. Plant and marine derived (n-3) polyunsaturated fatty acids do not affect blood coagulation and fibrinolytic factors in moderately hyperlipidemic humans. J Nutr 2003;133(7):2210-2213.

34. Fujioka S, Hamazaki K, Itomura M, Huan M, Nishizawa H, Sawazaki S et al. The effects of eicosapentaenoic acid-fortified food on inflammatory markers in healthy subjects--A randomized, placebo-controlled, double-blind study. J Nutr Sci Vitaminol (Tokyo) 2006;52(4):261-265.

35. Geelen A, Zock PL, Brouwer IA, Katan MB, Kors JA, Ritsema van Eck HJ et al. Effect of n-3 fatty acids from fish on electrocardiographic characteristics in patients with frequent premature ventricular complexes. Br J Nutr 2005;93(6):787-790.

36. Goodfellow J, Bellamy MF, Ramsey MW, Jones CJ, Lewis MJ. Dietary supplementation with marine omega-3 fatty acids improve systemic large artery endothelial function in subjects with hypercholesterolemia. J Am Coll Cardiol 2000;35(2):265-270.

37. Grigg LE, Kay TW, Valentine PA, Larkins R, Flower DJ, Manolas EG et al. Determinants of restenosis and lack of effect of dietary supplementation with eicosapentaenoic acid on the incidence of coronary artery restenosis after angioplasty. J Am Coll Cardiol 1989;13(3):665-672.

38. Hamazaki T, Sawazaki S, Asaoka E, Itomura M, Mizushima Y, Yazawa K et al. Docosahexaenoic acid-rich fish oil does not affect serum lipid concentrations of normolipidemic young adults. J Nutr 1996;126(11):2784-2789.

39. Hamazaki T, Sawazaki S, Itomura M, Asaoka E, Nagao Y, Nishimura N et al. The effect of docosahexaenoic acid on aggression in young adults. A placebo-controlled double-blind study. J Clin Invest 1996;97(4):1129-1133.

40. Harper M, Thom E, Klebanoff MA, Thorp J, Jr., Sorokin Y, Varner MW et al. Omega-3 fatty acid supplementation to prevent recurrent preterm birth: a randomized controlled trial. Obstet Gynecol 2010;115(2 Pt 1):234-242.

41. Harris WS, Ginsberg HN, Arunakul N, Shachter NS, Windsor SL, Adams M et al. Safety and efficacy of Omacor in severe hypertriglyceridemia. J Cardiovasc Risk 1997;4(5-6):385-391.

42. Heydari B, Abdullah S, Pottala JV, Shah R, Abbasi S, Mandry D et al. Effect of Omega-3 Acid Ethyl Esters on Left Ventricular Remodeling After Acute Myocardial Infarction: The OMEGA-REMODEL Randomized Clinical Trial. Circulation 2016;134(5):378-391.

43. Himmelfarb J, Phinney S, Ikizler TA, Kane J, McMonagle E, Miller G. Gamma-tocopherol and docosahexaenoic acid decrease inflammation in dialysis patients. J Ren Nutr 2007;17(5):296-304.

44. Holman RR, Paul S, Farmer A, Tucker L, Stratton IM, Neil HA et al. Atorvastatin in Factorial with Omega-3 EE90 Risk Reduction in Diabetes (AFORRD): a randomised controlled trial. Diabetologia 2009;52(1):50-59.

45. Hull MA, Sprange K, Hepburn T, Tan W, Shafayat A, Rees CJ et al. Eicosapentaenoic acid and aspirin, alone and in combination, for the prevention of colorectal adenomas (seAFOod Polyp Prevention trial): a multicentre, randomised, double-blind, placebo-controlled, 2 x 2 factorial trial. Lancet 2018;392(10164):2583-2594.

46. Irish AB, Viecelli AK, Hawley CM, Hooi LS, Pascoe EM, Paul-Brent PA et al. Effect of Fish Oil Supplementation and Aspirin Use on Arteriovenous Fistula Failure in Patients Requiring Hemodialysis: A Randomized Clinical Trial. JAMA Intern Med 2017;177(2):184-193.

47. Jacobson TA. A new pure omega-3 eicosapentaenoic acid ethyl ester (AMR101) for the management of hypertriglyceridemia: the MARINE trial. Expert Rev Cardiovasc Ther 2012;10(6):687-695.

48. Johansen O, Brekke M, Seljeflot I, Abdelnoor M, Arnesen H. N-3 fatty acids do not prevent restenosis after coronary angioplasty: results from the CART study. Coronary Angioplasty Restenosis Trial. J Am Coll Cardiol 1999;33(6):1619-1626.

49. Kastelein JJ, Maki KC, Susekov A, Ezhov M, Nordestgaard BG, Machielse BN et al. Omega-3 free fatty acids for the treatment of severe hypertriglyceridemia: the EpanoVa fOr Lowering Very high triglyceridEs (EVOLVE) trial. J Clin Lipidol 2014;8(1):94-106.

50. Kobayashi K, Hamazaki K, Fujioka S, Terao K, Yamamoto J, Kobayashi S. The effect of n - 3 PUFA/gamma-cyclodextrin complex on serum lipids in healthy volunteers--a randomized, placebo-controlled, double-blind trial. Asia Pac J Clin Nutr 2007;16(3):429-434.

51. Kowey PR, Reiffel JA, Ellenbogen KA, Naccarelli GV, Pratt CM. Efficacy and safety of prescription omega-3 fatty acids for the prevention of recurrent symptomatic atrial fibrillation: a randomized controlled trial. JAMA 2010;304(21):2363-2372.

52. Kristensen S, Schmidt EB, Schlemmer A, Rasmussen C, Lindgreen E, Johansen MB et al. The effect of marine n-3 polyunsaturated fatty acids on cardiac autonomic and hemodynamic function in patients with psoriatic arthritis: a randomised, double-blind, placebo-controlled trial. Lipids Health Dis 2016;15(1):216.

53. Maki KC, McKenney JM, Reeves MS, Lubin BC, Dicklin MR. Effects of adding prescription omega-3 acid ethyl esters to simvastatin (20 mg/day) on lipids and lipoprotein particles in men and women with mixed dyslipidemia. Am J Cardiol 2008;102(4):429-433.

54. Maki KC, Lubin BC, Reeves MS, Dicklin MR, Harris WS. Prescription omega-3 acid ethyl esters plus simvastatin 20 and 80 mg: effects in mixed dyslipidemia. J Clin Lipidol 2009;3(1):33-38.

55. Maki KC, Lawless AL, Kelley KM, Dicklin MR, Kaden VN, Schild AL et al. Effects of prescription omega-3-acid ethyl esters on fasting lipid profile in subjects with primary hypercholesterolemia. J Cardiovasc Pharmacol 2011;57(4):489-494.

56. Manson JE, Cook NR, Lee IM, Christen W, Bassuk SS, Mora S et al. Marine n-3 Fatty Acids and Prevention of Cardiovascular Disease and Cancer. N Engl J Med 2019;380(1):23-32.

57. Maresta A, Balduccelli M, Varani E, Marzilli M, Galli C, Heiman F et al. Prevention of postcoronary angioplasty restenosis by omega-3 fatty acids: main results of the Esapent for Prevention of Restenosis ITalian Study (ESPRIT). Am Heart J 2002;143(6):E5.

58. McGorry PD, Nelson B, Markulev C, Yuen HP, Schafer MR, Mossaheb N et al. Effect of omega-3 Polyunsaturated Fatty Acids in Young People at Ultrahigh Risk for Psychotic Disorders: The NEURAPRO Randomized Clinical Trial. JAMA psychiatry 2017;74(1):19-27.

59. Mendez-Sanchez N, Gonzalez V, Aguayo P, Sanchez JM, Tanimoto MA, Elizondo J et al. Fish oil (n-3) polyunsaturated fatty acids beneficially affect biliary cholesterol nucleation time in obese women losing weight. J Nutr 2001;131(9):2300-2303.

60. Mozaffarian D, Marchioli R, Macchia A, Silletta MG, Ferrazzi P, Gardner TJ et al. Fish oil and postoperative atrial fibrillation: the Omega-3 Fatty Acids for Prevention of Post-operative Atrial Fibrillation (OPERA) randomized trial. JAMA 2012;308(19):2001-2011.

61. Naesgaard PA, Grundt H, Nordoy AF, Staines H, Nilsen DWT. Vitamin D Uptake in Patients Treated with a High-Dosed Purified Omega-3 Compound in a Randomized Clinical Trial Following an Acute Myocardial Infarction. Front Cardiovasc Med 2017;4:41.

62. Noda Y, Nilsson C, Shimada H, Kim H, Lundstrom T, Yajima T. Safety, Tolerability, and Pharmacokinetics of Single and Multiple Oral Doses of an Omega-3-Carboxylic Acid Formulation in Healthy Male Japanese Subjects: A Phase 1 Single-Blind, Randomized, Placebo-Controlled Trial. Clin Pharmacol Drug Dev 2018;7(2):177-187.

63. Olsen SF, Sorensen JD, Secher NJ, Hedegaard M, Henriksen TB, Hansen HS et al. Randomised controlled trial of effect of fish-oil supplementation on pregnancy duration. Lancet 1992;339(8800):1003-1007.

64. Investigators OT, Bosch J, Gerstein HC, Dagenais GR, Diaz R, Dyal L et al. n-3 fatty acids and cardiovascular outcomes in patients with dysglycemia. N Engl J Med 2012;367(4):309-318.

65. Pooya S, Jalali MD, Jazayery AD, Saedisomeolia A, Eshraghian MR, Toorang F. The efficacy of omega-3 fatty acid supplementation on plasma homocysteine and malondialdehyde levels of type 2 diabetic patients. Nutr Metab Cardiovasc Dis 2010;20(5):326-331.

66. Poppitt SD, Howe CA, Lithander FE, Silvers KM, Lin RB, Croft J et al. Effects of moderate-dose omega-3 fish oil on cardiovascular risk factors and mood after ischemic stroke: a randomized, controlled trial. Stroke 2009;40(11):3485-3492.

67. Pradalier A, Bakouche P, Baudesson G, Delage A, Cornaille-Lafage G, Launay JM et al. Failure of omega-3 polyunsaturated fatty acids in prevention of migraine: a double-blind study versus placebo. Cephalalgia 2001;21(8):818-822.

68. Prisco D, Paniccia R, Bandinelli B, Filippini M, Francalanci I, Giusti B et al. Effect of medium-term supplementation with a moderate dose of n-3 polyunsaturated fatty acids on blood pressure in mild hypertensive patients. Thromb Res 1998;91(3):105-112.

69. Puri BK, Leavitt BR, Hayden MR, Ross CA, Rosenblatt A, Greenamyre JT et al. Ethyl-EPA in Huntington disease: a double-blind, randomized, placebo-controlled trial. Neurology 2005;65(2):286-292.

70. Quinn JF, Raman R, Thomas RG, Yurko-Mauro K, Nelson EB, Van Dyck C et al. Docosahexaenoic acid supplementation and cognitive decline in Alzheimer disease: a randomized trial. Jama 2010;304(17):1903-1911.

71. Radack KL, Deck CC, Huster GA. n-3 fatty acid effects on lipids, lipoproteins, and apolipoproteins at very low doses: results of a randomized controlled trial in hypertriglyceridemic subjects. Am J Clin Nutr 1990;51(4):599-605.

72. Raitt MH, Connor WE, Morris C, Kron J, Halperin B, Chugh SS et al. Fish oil supplementation and risk of ventricular tachycardia and ventricular fibrillation in patients with implantable defibrillators: a randomized controlled trial. JAMA 2005;293(23):2884-2891.

73. Reis GJ, Boucher TM, Sipperly ME, Silverman DI, McCabe CH, Baim DS et al. Randomised trial of fish oil for prevention of restenosis after coronary angioplasty. Lancet 1989;2(8656):177-181.

74. Risk, Prevention Study Collaborative G, Roncaglioni MC, Tombesi M, Avanzini F, Barlera S et al. n-3 fatty acids in patients with multiple cardiovascular risk factors. N Engl J Med 2013;368(19):1800-1808.

75. Sandhu N, Schetter SE, Liao J, Hartman TJ, Richie JP, McGinley J et al. Influence of Obesity on Breast Density Reduction by Omega-3 Fatty Acids: Evidence from a Randomized Clinical Trial. Cancer Prev Res (Phila) 2016;9(4):275-282.

76. Sirtori CR, Paoletti R, Mancini M, Crepaldi G, Manzato E, Rivellese A et al. N-3 fatty acids do not lead to an increased diabetic risk in patients with hyperlipidemia and abnormal glucose tolerance. Italian Fish Oil Multicenter Study. Am J Clin Nutr 1997;65(6):1874-1881.

77. Song J, Hu M, Li C, Yang B, Ding Q, Wang C et al. Dose-dependent effects of fish oil on cardio-metabolic biomarkers in healthy middle-aged and elderly Chinese people: a double-blind randomized controlled trial. Food Funct 2018;9(6):3235-3243.

78. Stroes ESG, Susekov AV, de Bruin TWA, Kvarnstrom M, Yang H, Davidson MH. Omega-3 carboxylic acids in patients with severe hypertriglyceridemia: EVOLVE II, a randomized, placebo-controlled trial. Journal of clinical lipidology 2018;12(2):321-330.

79. Tatsuno I, Saito Y, Kudou K, Ootake J. Efficacy and safety of TAK-085 compared with eicosapentaenoic acid in Japanese subjects with hypertriglyceridemia undergoing lifestyle modification: the omega-3 fatty acids randomized double-blind (ORD) study. Journal of clinical lipidology 2013;7(3):199-207.

80. Tatsuno I, Saito Y, Kudou K, Ootake J. Long-term safety and efficacy of TAK-085 in Japanese subjects with hypertriglyceridemia undergoing lifestyle modification: the omega-3 fatty acids randomized long-term (ORL) study. J Clin Lipidol 2013;7(6):615-625.

81. Toft I, Bonaa KH, Ingebretsen OC, Nordoy A, Jenssen T. Effects of n-3 polyunsaturated fatty acids on glucose homeostasis and blood pressure in essential hypertension. A randomized, controlled trial. Ann Intern Med 1995;123(12):911-918.

82. van Dam M, Stalenhoef AF, Wittekoek J, Trip MD, Prins MH, Kastelein JJ. Efficacy of Concentrated n-3 Fatty Acids in Hypertriglyceridaemia : A Comparison with Gemfibrozil. Clinical drug investigation 2001;21(3):175-181.

83. Yokoyama M, Origasa H, Matsuzaki M, Matsuzawa Y, Saito Y, Ishikawa Y et al. Effects of eicosapentaenoic acid on major coronary events in hypercholesterolaemic patients (JELIS): a randomised open-label, blinded endpoint analysis. Lancet 2007;369(9567):1090-1098.

84. Yurko-Mauro K, McCarthy D, Rom D, Nelson EB, Ryan AS, Blackwell A et al. Beneficial effects of docosahexaenoic acid on cognition in age-related cognitive decline. Alzheimers Dement 2010;6(6):456-464.

85. Nordoy A, Bonaa KH, Sandset PM, Hansen JB, Nilsen H. Effect of omega-3 fatty acids and simvastatin on hemostatic risk factors and postprandial hyperlipemia in patients with combined hyperlipemia. Arterioscler Thromb Vasc Biol 2000;20(1):259-265.

86. Su TC, Hwang JJ, Huang KC, Chiang FT, Chien KL, Wang KY et al. A Randomized, Double-Blind, Placebo-Controlled Clinical Trial to Assess the Efficacy and Safety of Ethyl-Ester Omega-3 Fatty Acid in Taiwanese Hypertriglyceridemic Patients. J Atheroscler Thromb 2017;24(3):275-289.

87. Svensson M, Schmidt EB, Jorgensen KA, Christensen JH, Group OS. N-3 fatty acids as secondary prevention against cardiovascular events in patients who undergo chronic hemodialysis: a randomized, placebo-controlled intervention trial. Clin J Am Soc Nephrol 2006;1(4):780-786.

88. von Schacky C, Angerer P, Kothny W, Theisen K, Mudra H. The effect of dietary omega-3 fatty acids on coronary atherosclerosis. A randomized, double-blind, placebo-controlled trial. Ann Intern Med 1999;130(7):554-562.

89. Rantanen JM, Riahi S, Johansen MB, Schmidt EB, Christensen JH. Effects of Marine n-3 Polyunsaturated Fatty Acids on Heart Rate Variability and Heart Rate in Patients on Chronic Dialysis: A Randomized Controlled Trial. Nutrients 2018;10(9).

90. Nodari S, Triggiani M, Campia U, Manerba A, Milesi G, Cesana BM et al. Effects of n-3 polyunsaturated fatty acids on left ventricular function and functional capacity in patients with dilated cardiomyopathy. J Am Coll Cardiol 2011;57(7):870-879.

91. Singer PW, M. can n-3 PUFA reduce cardiac arrhythmias? Results of a clinical trial. Prostalandins Leuko Essent Fatty Acids 2004;8(3):153-159.

92. Lok CE, Moist L, Hemmelgarn BR, Tonelli M, Vazquez MA, Dorval M et al. Effect of fish oil supplementation on graft patency and cardiovascular events among patients with new synthetic arteriovenous hemodialysis grafts: a randomized controlled trial. JAMA 2012;307(17):1809-1816.

93. Nilsen DW, Albrektsen G, Landmark K, Moen S, Aarsland T, Woie L. Effects of a high-dose concentrate of n-3 fatty acids or corn oil introduced early after an acute myocardial infarction on serum triacylglycerol and HDL cholesterol. Am J Clin Nutr 2001;74(1):50-56.

94. Nigam A, Talajic M, Roy D, Nattel S, Lambert J, Nozza A et al. Fish oil for the reduction of atrial fibrillation recurrence, inflammation, and oxidative stress. J Am Coll Cardiol 2014;64(14):1441-1448.

95. Farquharson AL, Metcalf RG, Sanders P, Stuklis R, Edwards JR, Gibson RA et al. Effect of dietary fish oil on atrial fibrillation after cardiac surgery. Am J Cardiol 2011;108(6):851-856.

96. Macchia A, Grancelli H, Varini S, Nul D, Laffaye N, Mariani J et al. Omega-3 fatty acids for the prevention of recurrent symptomatic atrial fibrillation: results of the FORWARD (Randomized Trial to Assess Efficacy of PUFA for the Maintenance of Sinus Rhythm in Persistent Atrial Fibrillation) trial. J Am Coll Cardiol 2013;61(4):463-468.

97. Budoff MJ, Muhlestein JB, Bhatt DL, Le Pa VT, May HT, Shaikh K et al. Effect of icosapent ethyl on progression of coronary atherosclerosis in patients with elevated triglycerides on statin therapy: a prospective, placebo-controlled randomized trial (EVAPORATE): interim results. Cardiovasc Res 2021;117(4):1070-1077.

98. Miller M, Ryan A, Reed RM, Goggins C, Sorkin J, Goldberg AP. Effect of Icosapent Ethyl on Gynoid Fat and Bone Mineral Health in the Metabolic Syndrome: A Preliminary Report. Clin Ther 2020;42(11):2226-2230.

99. Nicholls SJ, Lincoff AM, Garcia M, Bash D, Ballantyne CM, Barter PJ et al. Effect of High-Dose Omega-3 Fatty Acids vs Corn Oil on Major Adverse Cardiovascular Events in Patients at High Cardiovascular Risk: The STRENGTH Randomized Clinical Trial. JAMA 2020;324(22):2268-2280.

100. Scorletti E, Bhatia L, McCormick KG, Clough GF, Nash K, Hodson L et al. Effects of purified eicosapentaenoic and docosahexaenoic acids in nonalcoholic fatty liver disease: results from the Welcome* study. Hepatology 2014;60(4):1211-1221.

101. Qi L, Zhang Q, Zheng Z, Pei Z, Mao H, Jiang T et al. Treatment of Chinese Patients with Hypertriglyceridemia with a Pharmaceutical-Grade Preparation of Highly Purified Omega-3 Polyunsaturated Fatty Acid Ethyl Esters: Main Results of a Randomized, Double-Blind, Controlled Trial. Vasc Health Risk Manag 2021;17:571-580.

102. Eide IA, Reinholt FP, Jenssen T, Hartmann A, Schmidt EB, Asberg A et al. Effects of marine n-3 fatty acid supplementation in renal transplantation: A randomized controlled trial. Am J Transplant 2019;19(3):790-800.

103. Oscarsson J, Onnerhag K, Riserus U, Sunden M, Johansson L, Jansson PA et al. Effects of free omega-3 carboxylic acids and fenofibrate on liver fat content in patients with hypertriglyceridemia and non-alcoholic fatty liver disease: A double-blind, randomized, placebo-controlled study. J Clin Lipidol 2018;12(6):1390-1403 e1394.

104. Miller JP, Heath ID, Choraria SK, Shephard NW, Gajendragadkar RV, Harcus AW et al. Triglyceride lowering effect of MaxEPA fish lipid concentrate: a multicentre placebo controlled double blind study. Clin Chim Acta 1988;178(3):251-259.

105. Tavazzi L, Maggioni AP, Marchioli R, Barlera S, Franzosi MG, Latini R et al. Effect of n-3 polyunsaturated fatty acids in patients with chronic heart failure (the GISSI-HF trial): a randomised, double-blind, placebo-controlled trial. Lancet 2008;372(9645):1223-1230.

106. Ginty AT, Muldoon MF, Kuan DCH, Schirda B, Kamarck TW, Jennings JR et al. Omega-3 Supplementation and the Neural Correlates of Negative Affect and Impulsivity: A Double-Blind, Randomized, Placebo-Controlled Trial in Midlife Adults. Psychosom Med 2017;79(5):549-556.

107. Jamilian M, Samimi M, Ebrahimi FA, Hashemi T, Taghizadeh M, Razavi M et al. The effects of vitamin D and omega-3 fatty acid co-supplementation on glycemic control and lipid concentrations in patients with gestational diabetes. Journal of clinical lipidology 2017;11(2):459-468.

108. Barbosa MM, Melo AL, Damasceno NR. The benefits of omega-3 supplementation depend on adiponectin basal level and adiponectin increase after the supplementation: A randomized clinical trial. Nutrition 2017;34:7-13.

109. Bisgaard H, Stokholm J, Chawes BL, Vissing NH, Bjarnadottir E, Schoos AM et al. Fish Oil-Derived Fatty Acids in Pregnancy and Wheeze and Asthma in Offspring. N Engl J Med 2016;375(26):2530-2539.

110. Toupchian O, Sotoudeh G, Mansoori A, Abdollahi S, Ali Keshavarz S, Djalali M et al. DHA-enriched fish oil upregulates cyclin-dependent kinase inhibitor 2A (P16(INK)) expression and downregulates telomerase activity without modulating effects of PPARgamma Pro12Ala polymorphism in type 2 diabetic patients: A randomized, double-blind, placebo-controlled clinical trial. Clin Nutr 2018;37(1):91-98.

111. van Dijk SJ, Zhou J, Peters TJ, Buckley M, Sutcliffe B, Oytam Y et al. Effect of prenatal DHA supplementation on the infant epigenome: results from a randomized controlled trial. Clin Epigenetics 2016;8:114.

112. Rice SM, Hickie IB, Yung AR, Mackinnon A, Berk M, Davey C et al. Youth depression alleviation: the Fish Oil Youth Depression Study (YoDA-F): A randomized, double-blind, placebo-controlled treatment trial. Early intervention in psychiatry 2016;10(4):290-299.

113. Toupchian O, Sotoudeh G, Mansoori A, Nasli-Esfahani E, Djalali M, Keshavarz SA et al. Effects of DHA-enriched fish oil on monocyte/macrophage activation marker sCD163, asymmetric dimethyl arginine, and insulin resistance in type 2 diabetic patients. Journal of clinical lipidology 2016;10(4):798-807.

114. Bhatia L, Scorletti E, Curzen N, Clough GF, Calder PC, Byrne CD. Improvement in non-alcoholic fatty liver disease severity is associated with a reduction in carotid intima-media thickness progression. Atherosclerosis 2016;246:13-20.

115. Gold DR, Litonjua AA, Carey VJ, Manson JE, Buring JE, Lee IM et al. Lung VITAL: Rationale, design, and baseline characteristics of an ancillary study evaluating the effects of vitamin D and/or marine omega-3 fatty acid supplements on acute exacerbations of chronic respiratory disease, asthma control, pneumonia and lung function in adults. Contemp Clin Trials 2016;47:185-195.

116. Viecelli AK, Pascoe EM, Polkinghorne KR, Hawley CM, Paul-Brent PA, Badve SV et al. Baseline characteristics of the omega-3 fatty acids (Fish oils) and Aspirin in Vascular access OUtcomes in REnal Disease (FAVOURED) study. Nephrology 2016;21(3):217-228.

117. Told R, Schmidl D, Palkovits S, Boltz A, Gouya G, Wolzt M et al. Antioxidative capacity of a dietary supplement on retinal hemodynamic function in a human lipopolysaccharide (LPS) model. Invest Ophthalmol Vis Sci 2014;56(1):403-411.

118. Gustafson KM, Carlson SE, Colombo J, Yeh HW, Shaddy DJ, Li S et al. Effects of docosahexaenoic acid supplementation during pregnancy on fetal heart rate and variability: a randomized clinical trial. Prostaglandins Leukot Essent Fatty Acids 2013;88(5):331-338.

119. Mansoori A, Sotoudeh G, Djalali M, Eshraghian MR, Keramatipour M, Nasli-Esfahani E et al. Docosahexaenoic Acid-Rich Fish Oil Supplementation Improves Body Composition without Influence of the PPARgamma Pro12Ala Polymorphism in Patients with Type 2 Diabetes: A Randomized, Double-Blind, Placebo-Controlled Clinical Trial. J Nutrigenet Nutrigenomics 2015;8(4-6):195-204.

120. Naini AE, Keyvandarian N, Mortazavi M, Taheri S, Hosseini SM. Effect of Omega-3 fatty acids on blood pressure and serum lipids in continuous ambulatory peritoneal dialysis patients. J Res Pharm Pract 2015;4(3):135-141.

121. Guthrie KA, LaCroix AZ, Ensrud KE, Joffe H, Newton KM, Reed SD et al. Pooled Analysis of Six Pharmacologic and Nonpharmacologic Interventions for Vasomotor Symptoms. Obstetrics and gynecology 2015;126(2):413-422.

122. Bays HE, Ballantyne CM, Braeckman RA, Stirtan WG, Doyle RT, Jr., Philip S et al. Icosapent Ethyl (Eicosapentaenoic Acid Ethyl Ester): Effects Upon High-Sensitivity C-Reactive Protein and Lipid Parameters in Patients With Metabolic Syndrome. Metab Syndr Relat Disord 2015;13(6):239-247.

123. Gonzalez-Casanova I, Stein AD, Hao W, Garcia-Feregrino R, Barraza-Villarreal A, Romieu I et al. Prenatal Supplementation with Docosahexaenoic Acid Has No Effect on Growth through 60 Months of Age. J Nutr 2015;145(6):1330-1334.

124. Sabour H, Norouzi Javidan A, Latifi S, Shidfar F, Heshmat R, Emami Razavi SH et al. Omega-3 fatty acids' effect on leptin and adiponectin concentrations in patients with spinal cord injury: A double-blinded randomized clinical trial. J Spinal Cord Med 2015;38(5):599-606.

125. Mansoori A, Sotoudeh G, Djalali M, Eshraghian MR, Keramatipour M, Nasli-Esfahani E et al. Effect of DHA-rich fish oil on PPARgamma target genes related to lipid metabolism in type 2 diabetes: A randomized, double-blind, placebo-controlled clinical trial. Journal of clinical lipidology 2015;9(6):770-777.

126. Fayyazi A, Khajeh A, Ghazavi A, Sangestani M. Omega 3 in Childhood Migraines: a Double Blind Randomized Clinical Trial. Iran J Child Neurol 2016;10(1):9-13.

127. Norouzi Javidan A, Sabour H, Latifi S, Abrishamkar M, Soltani Z, Shidfar F et al. Does consumption of polyunsaturated fatty acids influence on neurorehabilitation in traumatic spinal cord-injured individuals? A double-blinded clinical trial. Spinal Cord 2014;52(5):378-382.

128. Reed SD, Guthrie KA, Newton KM, Anderson GL, Booth-LaForce C, Caan B et al. Menopausal quality of life: RCT of yoga, exercise, and omega-3 supplements. Am J Obstet Gynecol 2014;210(3):244 e241-211.

129. Mohammadi E, Rafraf M, Farzadi L, Asghari-Jafarabadi M, Sabour S. Effects of omega-3 fatty acids supplementation on serum adiponectin levels and some metabolic risk factors in women with polycystic ovary syndrome. Asia Pac J Clin Nutr 2012;21(4):511-518.

130. Eussen SR, Geleijnse JM, Giltay EJ, Rompelberg CJ, Klungel OH, Kromhout D. Effects of n-3 fatty acids on major cardiovascular events in statin users and non-users with a history of myocardial infarction. Eur Heart J 2012;33(13):1582-1588.

131. Ghoreishi Z, Esfahani A, Djazayeri A, Djalali M, Golestan B, Ayromlou H et al. Omega-3 fatty acids are protective against paclitaxel-induced peripheral neuropathy: a randomized double-blind placebo controlled trial. BMC Cancer 2012;12:355.

132. Malekshahi Moghadam A, Saedisomeolia A, Djalali M, Djazayery A, Pooya S, Sojoudi F. Efficacy of omega-3 fatty acid supplementation on serum levels of tumour necrosis factor-alpha, C-reactive protein and interleukin-2 in type 2 diabetes mellitus patients. Singapore medical journal 2012;53(9):615-619.

133. Murphy BL, Stoll AL, Harris PQ, Ravichandran C, Babb SM, Carlezon WA, Jr. et al. Omega-3 fatty acid treatment, with or without cytidine, fails to show therapeutic properties in bipolar disorder: a double-blind, randomized add-on clinical trial. J Clin Psychopharmacol 2012;32(5):699-703.

134. Bays HE, Braeckman RA, Ballantyne CM, Kastelein JJ, Otvos JD, Stirtan WG et al. Icosapent ethyl, a pure EPA omega-3 fatty acid: effects on lipoprotein particle concentration and size in patients with very high triglyceride levels (the MARINE study). J Clin Lipidol 2012;6(6):565-572.

135. Amminger GP, Chanen AM, Ohmann S, Klier CM, Mossaheb N, Bechdolf A et al. Omega-3 fatty acid supplementation in adolescents with borderline personality disorder and ultra-high risk criteria for psychosis: a post hoc subgroup analysis of a double-blind, randomized controlled trial. Canadian journal of psychiatry Revue canadienne de psychiatrie 2013;58(7):402-408.

136. Ahren B, Mari A, Fyfe CL, Tsofliou F, Sneddon AA, Wahle KW et al. Effects of conjugated linoleic acid plus n-3 polyunsaturated fatty acids on insulin secretion and estimated insulin sensitivity in men. Eur J Clin Nutr 2009;63(6):778-786.

137. Belanger SA, Vanasse M, Spahis S, Sylvestre MP, Lippe S, L'Heureux F et al. Omega-3 fatty acid treatment of children with attention-deficit hyperactivity disorder: A randomized, double-blind, placebo-controlled study. Paediatr Child Health 2009;14(2):89-98.

138. Kairaluoma L, Narhi V, Ahonen T, Westerholm J, Aro M. Do fatty acids help in overcoming reading difficulties? A double-blind, placebo-controlled study of the effects of eicosapentaenoic acid and carnosine supplementation on children with dyslexia. Child Care Health Dev 2009;35(1):112-119.

139. Vedin I, Cederholm T, Freund Levi Y, Basun H, Garlind A, Faxen Irving G et al. Effects of docosahexaenoic acid-rich n-3 fatty acid supplementation on cytokine release from blood mononuclear leukocytes: the OmegAD study. Am J Clin Nutr 2008;87(6):1616-1622.

140. McDaniel JC, Belury M, Ahijevych K, Blakely W. Omega-3 fatty acids effect on wound healing. Wound Repair Regen 2008;16(3):337-345.

141. Surette ME, Stull D, Lindemann J. The impact of a medical food containing gammalinolenic and eicosapentaenoic acids on asthma management and the quality of life of adult asthma patients. Curr Med Res Opin 2008;24(2):559-567.

142. Jellema A, Plat J, Mensink RP. Weight reduction, but not a moderate intake of fish oil, lowers concentrations of inflammatory markers and PAI-1 antigen in obese men during the fasting and postprandial state. European journal of clinical investigation 2004;34(11):766-773.

143. Bradbury J, Myers SP, Oliver C. An adaptogenic role for omega-3 fatty acids in stress; a randomised placebo controlled double blind intervention study (pilot) [ISRCTN22569553]. Nutr J 2004;3:20.

144. Itomura M, Hamazaki K, Sawazaki S, Kobayashi M, Terasawa K, Watanabe S et al. The effect of fish oil on physical aggression in schoolchildren--a randomized, double-blind, placebo-controlled trial. J Nutr Biochem 2005;16(3):163-171.

145. Hjerkinn EM, Seljeflot I, Ellingsen I, Berstad P, Hjermann I, Sandvik L et al. Influence of long-term intervention with dietary counseling, long-chain n-3 fatty acid supplements, or both on circulating markers of endothelial activation in men with long-standing hyperlipidemia. Am J Clin Nutr 2005;81(3):583-589.

146. Hamazaki K, Syafruddin D, Tunru IS, Azwir MF, Asih PB, Sawazaki S et al. The effects of docosahexaenoic acid-rich fish oil on behavior, school attendance rate and malaria infection in school children--a double-blind, randomized, placebo-controlled trial in Lampung, Indonesia. Asia Pac J Clin Nutr 2008;17(2):258-263.

147. Bechoua S, Dubois M, Vericel E, Chapuy P, Lagarde M, Prigent AF. Influence of very low dietary intake of marine oil on some functional aspects of immune cells in healthy elderly people. Br J Nutr 2003;89(4):523-531.

148. Dunstan JA, Mori TA, Barden A, Beilin LJ, Holt PG, Calder PC et al. Effects of n-3 polyunsaturated fatty acid supplementation in pregnancy on maternal and fetal erythrocyte fatty acid composition. Eur J Clin Nutr 2004;58(3):429-437.

149. Hirayama S, Hamazaki T, Terasawa K. Effect of docosahexaenoic acid-containing food administration on symptoms of attention-deficit/hyperactivity disorder - a placebo-controlled double-blind study. Eur J Clin Nutr 2004;58(3):467-473.

150. Lovegrove JA, Lovegrove SS, Lesauvage SV, Brady LM, Saini N, Minihane AM et al. Moderate fish-oil supplementation reverses low-platelet, long-chain n-3 polyunsaturated fatty acid status and reduces plasma triacylglycerol concentrations in British Indo-Asians. Am J Clin Nutr 2004;79(6):974-982.

151. Park Y, Jones PG, Harris WS. Triacylglycerol-rich lipoprotein margination: a potential surrogate for whole-body lipoprotein lipase activity and effects of eicosapentaenoic and docosahexaenoic acids. Am J Clin Nutr 2004;80(1):45-50.

152. Sundrarjun T, Komindr S, Archararit N, Dahlan W, Puchaiwatananon O, Angthararak S et al. Effects of n-3 fatty acids on serum interleukin-6, tumour necrosis factor-alpha and soluble tumour necrosis factor receptor p55 in active rheumatoid arthritis. J Int Med Res 2004;32(5):443-454.

153. Buckley R, Shewring B, Turner R, Yaqoob P, Minihane AM. Circulating triacylglycerol and apoE levels in response to EPA and docosahexaenoic acid supplementation in adult human subjects. Br J Nutr 2004;92(3):477-483.

154. Wallace JM, McCabe AJ, Roche HM, Higgins S, Robson PJ, Gilmore WS et al. The effect of low-dose fish oil supplementation on serum growth factors in healthy humans. Eur J Clin Nutr 2000;54(9):690-694.

155. Shearer GC, Borkowski K, Puumala SL, Harris WS, Pedersen TL, Newman JW. Abnormal lipoprotein oxylipins in metabolic syndrome and partial correction by omega-3 fatty acids. Prostaglandins Leukot Essent Fatty Acids 2018;128:1-10.

156. Hosogoe N, Ishikawa S, Yokoyama N, Kozuma K, Isshiki T. Add-on Antiplatelet Effects of Eicosapentaenoic Acid With Tailored Dose Setting in Patients on Dual Antiplatelet Therapy. Int Heart J 2017;58(4):481-485.

157. Matsumura K, Noguchi H, Nishi D, Hamazaki K, Hamazaki T, Matsuoka YJ. Effects of omega-3 polyunsaturated fatty acids on psychophysiological symptoms of post-traumatic stress disorder in accident survivors: A randomized, double-blind, placebo-controlled trial. J Affect Disord 2017;224:27-31.

158. Elajami TK, Alfaddagh A, Lakshminarayan D, Soliman M, Chandnani M, Welty FK. Eicosapentaenoic and Docosahexaenoic Acids Attenuate Progression of Albuminuria in Patients With Type 2 Diabetes Mellitus and Coronary Artery Disease. J Am Heart Assoc 2017;6(7).

159. Noguchi H, Nishi D, Matsumura K, Hamazaki K, Hamazaki T, Matsuoka YJ. Limited effect of omega-3 fatty acids on the quality of life in survivors of traumatic injury: A randomized, placebo-controlled trial. Prostaglandins Leukot Essent Fatty Acids 2017;127:1-5.

160. Tan A, Sullenbarger B, Prakash R, McDaniel JC. Supplementation with eicosapentaenoic acid and docosahexaenoic acid reduces high levels of circulating proinflammatory cytokines in aging adults: A randomized, controlled study. Prostaglandins Leukot Essent Fatty Acids 2018;132:23-29.

161. Hodson L, Bhatia L, Scorletti E, Smith DE, Jackson NC, Shojaee-Moradie F et al. Docosahexaenoic acid enrichment in NAFLD is associated with improvements in hepatic metabolism and hepatic insulin sensitivity: a pilot study. Eur J Clin Nutr 2017;71(10):1251.

162. Nakagawa I, Yokoyama S, Omoto K, Takeshima Y, Matsuda R, Nishimura F et al. omega-3 Fatty Acids Ethyl Esters Suppress Cerebral Vasospasm and Improve Clinical Outcome Following Aneurysmal Subarachnoid Hemorrhage. World Neurosurg 2017;99:457-464.

163. Escote X, Felix-Soriano E, Gayoso L, Huerta AE, Alvarado MA, Ansorena D et al. Effects of EPA and lipoic acid supplementation on circulating FGF21 and the fatty acid profile in overweight/obese women following a hypocaloric diet. Food Funct 2018;9(5):3028-3036.

164. Golzari MH, Javanbakht MH, Ghaedi E, Mohammadi H, Djalali M. Effect of Eicosapentaenoic acid (EPA) supplementation on cardiovascular markers in patients with type 2 diabetes mellitus: A randomized, double-blind, placebo-controlled trial. Diabetes Metab Syndr 2018;12(3):411-415.

165. Arm JP, Horton CE, Mencia-Huerta JM, House F, Eiser NM, Clark TJ et al. Effect of dietary supplementation with fish oil lipids on mild asthma. Thorax 1988;43(2):84-92.

166. Hamazaki K, Itomura M, Huan M, Nishizawa H, Sawazaki S, Tanouchi M et al. Effect of omega-3 fatty acid-containing phospholipids on blood catecholamine concentrations in healthy volunteers: a randomized, placebo-controlled, double-blind trial. Nutrition 2005;21(6):705-710.

167. Khor BS, Liaw SJ, Shih HC, Wang LS. Randomized, double blind, placebo-controlled trial of fish-oil-based lipid emulsion infusion for treatment of critically ill patients with severe sepsis. Asian J Surg 2011;34(1):1-10.

168. Kremer JM, Bigauoette J, Michalek AV, Timchalk MA, Lininger L, Rynes RI et al. Effects of manipulation of dietary fatty acids on clinical manifestations of rheumatoid arthritis. Lancet 1985;1(8422):184-187.

169. Mozurkewich EL, Clinton CM, Chilimigras JL, Hamilton SE, Allbaugh LJ, Berman DR et al. The Mothers, Omega-3, and Mental Health Study: a double-blind, randomized controlled trial. Am J Obstet Gynecol 2013;208(4):313 e311-319.

170. Rauch B, Schiele R, Schneider S, Diller F, Victor N, Gohlke H et al. OMEGA, a randomized, placebo-controlled trial to test the effect of highly purified omega-3 fatty acids on top of modern guideline-adjusted therapy after myocardial infarction. Circulation 2010;122(21):2152-2159.

171. Swanson B, Keithley J, Baum L, Leurgans S, Adeyemi O, Barnes LL et al. Effects of Fish Oil on HIV-Related Inflammation and Markers of Immunosenescence: A Randomized Clinical Trial. Journal of alternative and complementary medicine 2018;24(7):709-716.

172. Thies F, Garry JM, Yaqoob P, Rerkasem K, Williams J, Shearman CP et al. Association of n-3 polyunsaturated fatty acids with stability of atherosclerotic plaques: a randomised controlled trial. Lancet 2003;361(9356):477-485.

173. Lawrence R, Sorrell T. Eicosapentaenoic acid in cystic fibrosis: evidence of a pathogenetic role for leukotriene B4. Lancet 1993;342(8869):465-469.

174. Stammers T, Sibbald B, Freeling P. Fish oil in osteoarthritis. Lancet 1989;2(8661):503.

175. Bittiner SB, Tucker WF, Cartwright I, Bleehen SS. A double-blind, randomised, placebo-controlled trial of fish oil in psoriasis. Lancet 1988;1(8582):378-380.

176. Hamazaki T, Tateno S, Shishido H. Eicosapentaenoic acid and IgA nephropathy. Lancet 1984;1(8384):1017-1018.

177. Dietary supplementation with n-3 polyunsaturated fatty acids and vitamin E after myocardial infarction: results of the GISSI-Prevenzione trial. Gruppo Italiano per lo Studio della Sopravvivenza nell'Infarto miocardico. Lancet 1999;354(9177):447-455.

178. Chew EY, Clemons TE, Agron E, Launer LJ, Grodstein F, Bernstein PS et al. Effect of Omega-3 Fatty Acids, Lutein/Zeaxanthin, or Other Nutrient Supplementation on Cognitive Function: The AREDS2 Randomized Clinical Trial. Jama 2015;314(8):791-801.

179. Gould JF, Treyvaud K, Yelland LN, Anderson PJ, Smithers LG, Gibson RA et al. Does n-3 LCPUFA supplementation during pregnancy increase the IQ of children at school age? Follow-up of a randomised controlled trial. BMJ Open 2016;6(5):e011465.

180. Galan P, Kesse-Guyot E, Czernichow S, Briancon S, Blacher J, Hercberg S et al. Effects of B vitamins and omega 3 fatty acids on cardiovascular diseases: a randomised placebo controlled trial. BMJ 2010;341:c6273.

181. Andrieu S, Guyonnet S, Coley N, Cantet C, Bonnefoy M, Bordes S et al. Effect of long-term omega 3 polyunsaturated fatty acid supplementation with or without multidomain intervention on cognitive function in elderly adults with memory complaints (MAPT): a randomised, placebo-controlled trial. Lancet Neurol 2017;16(5):377-389.

182. Age-Related Eye Disease Study 2 Research G. Lutein + zeaxanthin and omega-3 fatty acids for age-related macular degeneration: the Age-Related Eye Disease Study 2 (AREDS2) randomized clinical trial. JAMA 2013;309(19):2005-2015.

183. Kromhout D, Giltay EJ, Geleijnse JM, Alpha Omega Trial G. n-3 fatty acids and cardiovascular events after myocardial infarction. N Engl J Med 2010;363(21):2015-2026.

184. Berstad P, Seljeflot I, Veierod MB, Hjerkinn EM, Arnesen H, Pedersen JI. Supplementation with fish oil affects the association between very long-chain n-3 polyunsaturated fatty acids in serum non-esterified fatty acids and soluble vascular cell adhesion molecule-1. Clin Sci (Lond) 2003;105(1):13-20.

185. Dunstan JA, Mori TA, Barden A, Beilin LJ, Taylor AL, Holt PG et al. Maternal fish oil supplementation in pregnancy reduces interleukin-13 levels in cord blood of infants at high risk of atopy. Clin Exp Allergy 2003;33(4):442-448.

186. Durrington PN, Bhatnagar D, Mackness MI, Morgan J, Julier K, Khan MA et al. An omega-3 polyunsaturated fatty acid concentrate administered for one year decreased triglycerides in simvastatin treated patients with coronary heart disease and persisting hypertriglyceridaemia. Heart 2001;85(5):544-548.

187. Furuhjelm C, Warstedt K, Larsson J, Fredriksson M, Bottcher MF, Falth-Magnusson K et al. Fish oil supplementation in pregnancy and lactation may decrease the risk of infant allergy. Acta Paediatr 2009;98(9):1461-1467.

188. Holm T, Andreassen AK, Aukrust P, Andersen K, Geiran OR, Kjekshus J et al. Omega-3 fatty acids improve blood pressure control and preserve renal function in hypertensive heart transplant recipients. Eur Heart J 2001;22(5):428-436.

189. Qin Y, Nyheim H, Haram EM, Moritz JM, Hustvedt SO. A novel self-micro-emulsifying delivery system (SMEDS) formulation significantly improves the fasting absorption of EPA and DHA from a single dose of an omega-3 ethyl ester concentrate. Lipids Health Dis 2017;16(1):204.

190. Saravanan P, Bridgewater B, West AL, O'Neill SC, Calder PC, Davidson NC. Omega-3 fatty acid supplementation does not reduce risk of atrial fibrillation after coronary artery bypass surgery: a randomized, double-blind, placebo-controlled clinical trial. Circ Arrhythm Electrophysiol 2010;3(1):46-53.

191. Van Biervliet S, Devos M, Delhaye T, Van Biervliet JP, Robberecht E, Christophe A. Oral DHA supplementation in DeltaF508 homozygous cystic fibrosis patients. Prostaglandins Leukot Essent Fatty Acids 2008;78(2):109-115.

192. Garbagnati F, Cairella G, De Martino A, Multari M, Scognamiglio U, Venturiero V et al. Is antioxidant and n-3 supplementation able to improve functional status in poststroke patients? Results from the Nutristroke Trial. Cerebrovasc Dis 2009;27(4):375-383.

193. Grundt H, Nilsen DW, Hetland O, Mansoor MA. Clinical outcome and atherothrombogenic risk profile after prolonged wash-out following long-term treatment with high doses of n-3 PUFAs in patients with an acute myocardial infarction. Clin Nutr 2004;23(4):491-500.

194. Leaf A, Albert CM, Josephson M, Steinhaus D, Kluger J, Kang JX et al. Prevention of fatal arrhythmias in high-risk subjects by fish oil n-3 fatty acid intake. Circulation 2005;112(18):2762-2768.

195. West AL, Kindberg GM, Hustvedt SO, Calder PC. A Novel Self-Micro-Emulsifying Delivery System Enhances Enrichment of Eicosapentaenoic Acid and Docosahexaenoic Acid after Single and Repeated Dosing in Healthy Adults in a Randomized Trial. J Nutr 2018;148(11):1704-1715.

196. Lefort N, LeBlanc R, Surette ME. Dietary Buglossoides Arvensis Oil Increases Circulating n-3 Polyunsaturated Fatty Acids in a Dose-Dependent Manner and Enhances Lipopolysaccharide-Stimulated Whole Blood Interleukin-10-A Randomized Placebo-Controlled Trial. Nutrients 2017;9(3).

197. Pribis P. Effects of Walnut Consumption on Mood in Young Adults-A Randomized Controlled Trial. Nutrients 2016;8(11).

198. Bechdolf A, Muller H, Stutzer H, Wagner M, Maier W, Lautenschlager M et al. Rationale and baseline characteristics of PREVENT: a second-generation intervention trial in subjects at-risk (prodromal) of developing first-episode psychosis evaluating cognitive behavior therapy, aripiprazole, and placebo for the prevention of psychosis. Schizophr Bull 2011;37 Suppl 2(Suppl 2):S111-121.

199. Ormarsson OT, Geirsson T, Bjornsson ES, Jonsson T, Moller PH, Loftsson T et al. Clinical trial: marine lipid suppositories as laxatives. Mar Drugs 2012;10(9):2047-2054.

200. Lemke SL, Vicini JL, Su H, Goldstein DA, Nemeth MA, Krul ES et al. Dietary intake of stearidonic acid-enriched soybean oil increases the omega-3 index: randomized, double-blind clinical study of efficacy and safety. Am J Clin Nutr 2010;92(4):766-775.

201. Haugaard SB, Vaag A, Mu H, Madsbad S. Skeletal muscle structural lipids improve during weight-maintenance after a very low calorie dietary intervention. Lipids Health Dis 2009;8:34.

202. Bemelmans WJ, Lefrandt JD, Feskens EJ, van Haelst PL, Broer J, Meyboom-de Jong B et al. Increased alpha-linolenic acid intake lowers C-reactive protein, but has no effect on markers of atherosclerosis. Eur J Clin Nutr 2004;58(7):1083-1089.

203. Hamazaki K, Maekawa M, Toyota T, Dean B, Hamazaki T, Yoshikawa T. Fatty acid composition of the postmortem prefrontal cortex of patients with schizophrenia, bipolar disorder, and major depressive disorder. Psychiatry research 2015;227(2-3):353-359.

204. Hamazaki K, Kawaguchi Y, Nakano M, Yasuda T, Seki S, Hori T et al. Mead acid (20:3n-9) and n-3 polyunsaturated fatty acids are not associated with risk of posterior longitudinal ligament ossification: results of a case-control study. Prostaglandins Leukot Essent Fatty Acids 2015;96:31-36.

205. Schwartz LM, Woloshin S, Lu Z, Ross KM, Tessema FA, Peter D et al. Randomized Study of Providing Evidence Context to Mitigate Physician Misinterpretation Arising From Off-Label Drug Promotion. Circ Cardiovasc Qual Outcomes 2019;12(11):e006073.

206. Chaitman BR, Alexander KP, Cyr DD, Berger JS, Reynolds HR, Bangalore S et al. Myocardial Infarction in the ISCHEMIA Trial: Impact of Different Definitions on Incidence, Prognosis, and Treatment Comparisons. Circulation 2021;143(8):790-804.

207. Soares AA, Loucana PMC, Nasi EP, Sousa KMH, Sa OMS, Silva-Neto RP. A double- blind, randomized, and placebo-controlled clinical trial with omega-3 polyunsaturated fatty acids (OPFA ɷ-3) for the prevention of migraine in chronic migraine patients using amitriptyline. Nutr Neurosci 2018;21(3):219-223.

208. Giorlandino C, Giannarelli D. Effect of vaginally administered DHA fatty acids on pregnancy outcome in high risk pregnancies for preterm delivery: a double blinded randomised controlled trial. J Prenat Med 2013;7(3):42-45.

209. Khandouzi N, Shidfar F, Agah S, Hosseini AF, Dehnad A. Comparison of the Effects of Eicosapentaenoic Acid and Docosahexaenoic Acid on the Eradication of Helicobacter pylori Infection, Serum Inflammatory Factors and Total Antioxidant Capacity. Iran J Pharm Res 2015;14(1):149-157.

210. Darghosian L, Free M, Li J, Gebretsadik T, Bian A, Shintani A et al. Effect of omega-three polyunsaturated fatty acids on inflammation, oxidative stress, and recurrence of atrial fibrillation. Am J Cardiol 2015;115(2):196-201.

211. Ballantyne CM, Braeckman RA, Bays HE, Kastelein JJ, Otvos JD, Stirtan WG et al. Effects of icosapent ethyl on lipoprotein particle concentration and size in statin-treated patients with persistent high triglycerides (the ANCHOR Study). J Clin Lipidol 2015;9(3):377-383.

212. Almaas AN, Tamnes CK, Nakstad B, Henriksen C, Walhovd KB, Fjell AM et al. Long-chain polyunsaturated fatty acids and cognition in VLBW infants at 8 years: an RCT. Pediatrics 2015;135(6):972-980.

213. Hershman DL, Unger JM, Crew KD, Awad D, Dakhil SR, Gralow J et al. Randomized Multicenter Placebo-Controlled Trial of Omega-3 Fatty Acids for the Control of Aromatase Inhibitor-Induced Musculoskeletal Pain: SWOG S0927. J Clin Oncol 2015;33(17):1910-1917.

214. Elwakeel NM, Hazaa HH. Effect of omega 3 fatty acids plus low-dose aspirin on both clinical and biochemical profiles of patients with chronic periodontitis and type 2 diabetes: a randomized double blind placebo-controlled study. J Periodontal Res 2015;50(6):721-729.

215. Salehi B, Mohammadbeigi A, Sheykholeslam H, Moshiri E, Dorreh F. Omega-3 and Zinc supplementation as complementary therapies in children with attention-deficit/hyperactivity disorder. J Res Pharm Pract 2016;5(1):22-26.

216. Smith HE, Ryan KN, Stephenson KB, Westcott C, Thakwalakwa C, Maleta K et al. Multiple micronutrient supplementation transiently ameliorates environmental enteropathy in Malawian children aged 12-35 months in a randomized controlled clinical trial. J Nutr 2014;144(12):2059-2065.

217. Su KP, Lai HC, Yang HT, Su WP, Peng CY, Chang JP et al. Omega-3 fatty acids in the prevention of interferon-alpha-induced depression: results from a randomized, controlled trial. Biol Psychiatry 2014;76(7):559-566.

218. Naqvi AZ, Hasturk H, Mu L, Phillips RS, Davis RB, Halem S et al. Docosahexaenoic Acid and Periodontitis in Adults: A Randomized Controlled Trial. J Dent Res 2014;93(8):767-773.

219. Mahmoodi MR, Kimiagar M, Mehrabi Y. The effects of omega-3 plus vitamin E and zinc plus vitamin C supplementation on cardiovascular risk markers in postmenopausal women with type 2 diabetes. Ther Adv Endocrinol Metab 2014;5(4):67-76.

220. Rodrigo R, Gutierrez R, Fernandez R, Guzman P. Ageing improves the antioxidant response against postoperative atrial fibrillation: a randomized controlled trial. Interact Cardiovasc Thorac Surg 2012;15(2):209-214.

221. Sabour H, Larijani B, Vafa MR, Hadian MR, Heshmat R, Meybodi HA et al. The effects of n-3 fatty acids on inflammatory cytokines in osteoporotic spinal cord injured patients: A randomized clinical trial. J Res Med Sci 2012;17(4):322-327.

222. Szabo de Edelenyi F, Vergnaud AC, Ahluwalia N, Julia C, Hercberg S, Blacher J et al. Effect of B-vitamins and n-3 PUFA supplementation for 5 years on blood pressure in patients with CVD. Br J Nutr 2012;107(6):921-927.

223. Rice TW, Wheeler AP, Thompson BT, deBoisblanc BP, Steingrub J, Rock P et al. Enteral omega-3 fatty acid, gamma-linolenic acid, and antioxidant supplementation in acute lung injury. JAMA 2011;306(14):1574-1581.

224. Elkhouli AM. The efficacy of host response modulation therapy (omega-3 plus low-dose aspirin) as an adjunctive treatment of chronic periodontitis (clinical and biochemical study). J Periodontal Res 2011;46(2):261-268.

225. El-Sharkawy H, Aboelsaad N, Eliwa M, Darweesh M, Alshahat M, Kantarci A et al. Adjunctive treatment of chronic periodontitis with daily dietary supplementation with omega-3 Fatty acids and low-dose aspirin. J Periodontol 2010;81(11):1635-1643.

226. Dawczynski C, Martin L, Wagner A, Jahreis G. n-3 LC-PUFA-enriched dairy products are able to reduce cardiovascular risk factors: a double-blind, cross-over study. Clin Nutr 2010;29(5):592-599.

227. Einvik G, Klemsdal TO, Sandvik L, Hjerkinn EM. A randomized clinical trial on n-3 polyunsaturated fatty acids supplementation and all-cause mortality in elderly men at high cardiovascular risk. Eur J Cardiovasc Prev Rehabil 2010;17(5):588-592.

228. Lindemann J, David Pampe E, Peterkin JJ, Orozco-Cronin P, Belofsky G, Stull D. Clinical study of the effects on asthma-related QOL and asthma management of a medical food in adult asthma patients. Curr Med Res Opin 2009;25(12):2865-2875.

229. Harrison RA, Sagara M, Rajpura A, Armitage L, Birt N, Birt CA et al. Can foods with added soya-protein or fish-oil reduce risk factors for coronary disease? A factorial randomised controlled trial. Nutr Metab Cardiovasc Dis 2004;14(6):344-350.

230. Campagnoli C, Abba C, Ambroggio S, Peris C, Perona M, Sanseverino P. Polyunsaturated fatty acids (PUFAs) might reduce hot flushes: an indication from two controlled trials on soy isoflavones alone and with a PUFA supplement. Maturitas 2005;51(2):127-134.

231. Phillips T, Childs AC, Dreon DM, Phinney S, Leeuwenburgh C. A dietary supplement attenuates IL-6 and CRP after eccentric exercise in untrained males. Med Sci Sports Exerc 2003;35(12):2032-2037.

232. Hansen J, Grimsgaard S, Nordoy A, Bonaa KH. Dietary supplementation with highly purified eicosapentaenoic acid and docosahexaenoic acid does not influence PAI-1 activity. Thrombosis research 2000;98(2):123-132.

233. Stark KD, Park EJ, Maines VA, Holub BJ. Effect of a fish-oil concentrate on serum lipids in postmenopausal women receiving and not receiving hormone replacement therapy in a placebo-controlled, double-blind trial. Am J Clin Nutr 2000;72(2):389-394.

234. Bemelmans WJ, Muskiet FA, Feskens EJ, de Vries JH, Broer J, May JF et al. Associations of alpha-linolenic acid and linoleic acid with risk factors for coronary heart disease. Eur J Clin Nutr 2000;54(12):865-871.

235. Korobelnik JF, Rougier MB, Delyfer MN, Bron A, Merle BMJ, Savel H et al. Effect of Dietary Supplementation With Lutein, Zeaxanthin, and omega-3 on Macular Pigment: A Randomized Clinical Trial. JAMA Ophthalmol 2017;135(11):1259-1266.

236. Sala-Vila A, Diaz-Lopez A, Valls-Pedret C, Cofan M, Garcia-Layana A, Lamuela-Raventos RM et al. Dietary Marine omega-3 Fatty Acids and Incident Sight-Threatening Retinopathy in Middle-Aged and Older Individuals With Type 2 Diabetes: Prospective Investigation From the PREDIMED Trial. JAMA Ophthalmol 2016;134(10):1142-1149.

237. van Zanten AR, Sztark F, Kaisers UX, Zielmann S, Felbinger TW, Sablotzki AR et al. High-protein enteral nutrition enriched with immune-modulating nutrients vs standard high-protein enteral nutrition and nosocomial infections in the ICU: a randomized clinical trial. JAMA 2014;312(5):514-524.

238. Leng GC, Lee AJ, Fowkes FG, Jepson RG, Lowe GD, Skinner ER et al. Randomized controlled trial of gamma-linolenic acid and eicosapentaenoic acid in peripheral arterial disease. Clin Nutr 1998;17(6):265-271.

239. Writing Group for the ARG, Bonds DE, Harrington M, Worrall BB, Bertoni AG, Eaton CB et al. Effect of long-chain omega-3 fatty acids and lutein + zeaxanthin supplements on cardiovascular outcomes: results of the Age-Related Eye Disease Study 2 (AREDS2) randomized clinical trial. JAMA Intern Med 2014;174(5):763-771.

240. Eritsland J, Arnesen H, Gronseth K, Fjeld NB, Abdelnoor M. Effect of dietary supplementation with n-3 fatty acids on coronary artery bypass graft patency. Am J Cardiol 1996;77(1):31-36.

241. Shakeri J, Khanegi M, Golshani S, Farnia V, Tatari F, Alikhani M et al. Effects of Omega-3 Supplement in the Treatment of Patients with Bipolar I Disorder. Int J Prev Med 2016;7:77.

242. Cockayne NL, Duffy SL, Bonomally R, English A, Amminger PG, Mackinnon A et al. The Beyond Ageing Project Phase 2--a double-blind, selective prevention, randomised, placebo-controlled trial of omega-3 fatty acids and sertraline in an older age cohort at risk for depression: study protocol for a randomized controlled trial. Trials 2015;16:247.

243. Linecker M, Limani P, Botea F, Popescu I, Alikhanov R, Efanov M et al. "A randomized, double-blind study of the effects of omega-3 fatty acids (Omegaven) on outcome after major liver resection". BMC gastroenterology 2015;15:102.

244. Meldrum SJ, D'Vaz N, Dunstan J, Mori TA, Prescott SL. The Infant Fish Oil Supplementation Study (IFOS): design and research protocol of a double-blind, randomised controlled n--3 LCPUFA intervention trial in term infants. Contemporary clinical trials 2011;32(5):771-778.

245. Cruz-Mora J, Martinez-Hernandez NE, Martin del Campo-Lopez F, Viramontes-Horner D, Vizmanos-Lamotte B, Munoz-Valle JF et al. Effects of a symbiotic on gut microbiota in Mexican patients with end-stage renal disease. Journal of renal nutrition : the official journal of the Council on Renal Nutrition of the National Kidney Foundation 2014;24(5):330-335.

246. Bragt MC, Mensink RP. Comparison of the effects of n-3 long chain polyunsaturated fatty acids and fenofibrate on markers of inflammation and vascular function, and on the serum lipoprotein profile in overweight and obese subjects. Nutr Metab Cardiovasc Dis 2012;22(11):966-973.

247. Olano-Martin E, Anil E, Caslake MJ, Packard CJ, Bedford D, Stewart G et al. Contribution of apolipoprotein E genotype and docosahexaenoic acid to the LDL-cholesterol response to fish oil. Atherosclerosis 2010;209(1):104-110.

248. van der Tempel H, Tulleken JE, Limburg PC, Muskiet FA, van Rijswijk MH. Effects of fish oil supplementation in rheumatoid arthritis. Ann Rheum Dis 1990;49(2):76-80.

249. Green P, Fuchs J, Schoenfeld N, Leibovici L, Lurie Y, Beigel Y et al. Effects of fish-oil ingestion on cardiovascular risk factors in hyperlipidemic subjects in Israel: a randomized, double-blind crossover study. Am J Clin Nutr 1990;52(6):1118-1124.

250. Annuzzi G, Rivellese A, Capaldo B, Di Marino L, Iovine C, Marotta G et al. A controlled study on the effects of n-3 fatty acids on lipid and glucose metabolism in non-insulin-dependent diabetic patients. Atherosclerosis 1991;87(1):65-73.

251. Donnelly SM, Ali MA, Churchill DN. Effect of n-3 fatty acids from fish oil on hemostasis, blood pressure, and lipid profile of dialysis patients. J Am Soc Nephrol 1992;2(11):1634-1639.

252. de Fijter CW, Popp-Snijders C, Oe LP, Tran DD, van der Meulen J, Donker AJ. Does additional treatment with fish oil mitigate the side effects of recombinant human erythropoietin in dialysis patients? Haematologica 1995;80(4):332-334.

253. Marangell LB, Suppes T, Ketter TA, Dennehy EB, Zboyan H, Kertz B et al. Omega-3 fatty acids in bipolar disorder: clinical and research considerations. Prostaglandins Leukot Essent Fatty Acids 2006;75(4-5):315-321.

254. Mickleborough TD, Murray RL, Ionescu AA, Lindley MR. Fish oil supplementation reduces severity of exercise-induced bronchoconstriction in elite athletes. Am J Respir Crit Care Med 2003;168(10):1181-1189.

255. Stark KD, Holub BJ. Differential eicosapentaenoic acid elevations and altered cardiovascular disease risk factor responses after supplementation with docosahexaenoic acid in postmenopausal women receiving and not receiving hormone replacement therapy. Am J Clin Nutr 2004;79(5):765-773.

256. Lopez-Toledano MA, Thorsteinsson T, Daak AA, Maki KC, Johns C, Rabinowicz AL et al. Minimal food effect for eicosapentaenoic acid and docosahexaenoic acid bioavailability from omega-3-acid ethyl esters with an Advanced Lipid Technologies(TM) (ALT((R)))-based formulation. Journal of clinical lipidology 2017;11(2):394-405.

257. Maki KC, Bobotas G, Dicklin MR, Huebner M, Keane WF. Effects of MAT9001 containing eicosapentaenoic acid and docosapentaenoic acid, compared to eicosapentaenoic acid ethyl esters, on triglycerides, lipoprotein cholesterol, and related variables. Journal of clinical lipidology 2017;11(1):102-109.

258. Dai Perrard XY, Lian Z, Bobotas G, Dicklin MR, Maki KC, Wu H. Effects of n-3 fatty acid treatment on monocyte phenotypes in humans with hypertriglyceridemia. Journal of clinical lipidology 2017;11(6):1361-1371.

259. Manni A, Richie JP, Schetter SE, Calcagnotto A, Trushin N, Aliaga C et al. Stearoyl-CoA desaturase-1, a novel target of omega-3 fatty acids for reducing breast cancer risk in obese postmenopausal women. Eur J Clin Nutr 2017;71(6):762-765.

260. Lopez-Toledano MA, Thorsteinsson T, Daak A, Maki KC, Johns C, Rabinowicz AL et al. A Novel omega-3 Acid Ethyl Ester Formulation Incorporating Advanced Lipid Technologies(TM) (ALT((R))) Improves Docosahexaenoic Acid and Eicosapentaenoic Acid Bioavailability Compared with Lovaza((R)). Clin Ther 2017;39(3):581-591.

261. Prentice KJ, Wendell SG, Liu Y, Eversley JA, Salvatore SR, Mohan H et al. CMPF, a Metabolite Formed Upon Prescription Omega-3-Acid Ethyl Ester Supplementation, Prevents and Reverses Steatosis. EBioMedicine 2018;27:200-213.

262. Alfaddagh A, Elajami TK, Saleh M, Elajami M, Bistrian BR, Welty FK. The effect of eicosapentaenoic and docosahexaenoic acids on physical function, exercise, and joint replacement in patients with coronary artery disease: A secondary analysis of a randomized clinical trial. J Clin Lipidol 2018;12(4):937-947 e932.

263. Nakagawa I, Park HS, Yokoyama S, Wada T, Yamada S, Motoyama Y et al. Pretreatment with and ongoing use of omega-3 fatty acid ethyl esters reduce the slow-flow phenomenon and prevent in-stent restenosis in patients undergoing carotid artery stenting. J Vasc Surg 2017;66(1):122-129.

264. Endres S, Ghorbani R, Kelley VE, Georgilis K, Lonnemann G, van der Meer JW et al. The effect of dietary supplementation with n-3 polyunsaturated fatty acids on the synthesis of interleukin-1 and tumor necrosis factor by mononuclear cells. N Engl J Med 1989;320(5):265-271.

265. Phillipson BE, Rothrock DW, Connor WE, Harris WS, Illingworth DR. Reduction of plasma lipids, lipoproteins, and apoproteins by dietary fish oils in patients with hypertriglyceridemia. N Engl J Med 1985;312(19):1210-1216.

266. Abe K, Uwagawa T, Haruki K, Takano Y, Onda S, Sakamoto T et al. Effects of omega-3 Fatty Acid Supplementation in Patients with Bile Duct or Pancreatic Cancer Undergoing Chemotherapy. Anticancer Res 2018;38(4):2369-2375.

267. Schrepf R, Limmert T, Claus Weber P, Theisen K, Sellmayer A. Immediate effects of n-3 fatty acid infusion on the induction of sustained ventricular tachycardia. Lancet 2004;363(9419):1441-1442.

268. Sinclair H, Gale M. Eicosapentaenoic acid in fat. Lancet 1987;1(8543):1202.

269. Saynor R. Effects of omega-3 fatty acids on serum lipids. Lancet 1984;2(8404):696-697.

270. Daenen LG, Cirkel GA, Houthuijzen JM, Gerrits J, Oosterom I, Roodhart JM et al. Increased Plasma Levels of Chemoresistance-Inducing Fatty Acid 16:4(n-3) After Consumption of Fish and Fish Oil. JAMA Oncol 2015;1(3):350-358.

271. Arnold C, Jentsch S, Dawczynski J, Bohm V. Age-related macular degeneration: Effects of a short-term intervention with an oleaginous kale extract--a pilot study. Nutrition 2013;29(11-12):1412-1417.

272. Esposito K, Marfella R, Ciotola M, Di Palo C, Giugliano F, Giugliano G et al. Effect of a mediterranean-style diet on endothelial dysfunction and markers of vascular inflammation in the metabolic syndrome: a randomized trial. JAMA 2004;292(12):1440-1446.

273. Esposito K, Pontillo A, Di Palo C, Giugliano G, Masella M, Marfella R et al. Effect of weight loss and lifestyle changes on vascular inflammatory markers in obese women: a randomized trial. JAMA 2003;289(14):1799-1804.

274. Jula A, Marniemi J, Huupponen R, Virtanen A, Rastas M, Ronnemaa T. Effects of diet and simvastatin on serum lipids, insulin, and antioxidants in hypercholesterolemic men: a randomized controlled trial. JAMA 2002;287(5):598-605.

275. Middleton P, Gomersall JC, Gould JF, Shepherd E, Olsen SF, Makrides M. Omega-3 fatty acid addition during pregnancy. Cochrane Database Syst Rev 2018;11(11):CD003402.

276. Hill CL, March LM, Aitken D, Lester SE, Battersby R, Hynes K et al. Fish oil in knee osteoarthritis: a randomised clinical trial of low dose versus high dose. Ann Rheum Dis 2016;75(1):23-29.

277. Proudman SM, James MJ, Spargo LD, Metcalf RG, Sullivan TR, Rischmueller M et al. Fish oil in recent onset rheumatoid arthritis: a randomised, double-blind controlled trial within algorithm-based drug use. Ann Rheum Dis 2015;74(1):89-95.

278. Burr ML, Ashfield-Watt PA, Dunstan FD, Fehily AM, Breay P, Ashton T et al. Lack of benefit of dietary advice to men with angina: results of a controlled trial. Eur J Clin Nutr 2003;57(2):193-200.

279. Nosaka K, Miyoshi T, Iwamoto M, Kajiya M, Okawa K, Tsukuda S et al. Early initiation of eicosapentaenoic acid and statin treatment is associated with better clinical outcomes than statin alone in patients with acute coronary syndromes: 1-year outcomes of a randomized controlled study. Int J Cardiol 2017;228:173-179.

280. Doi M, Nosaka K, Miyoshi T, Iwamoto M, Kajiya M, Okawa K et al. Early eicosapentaenoic acid treatment after percutaneous coronary intervention reduces acute inflammatory responses and ventricular arrhythmias in patients with acute myocardial infarction: a randomized, controlled study. Int J Cardiol 2014;176(3):577-582.

281. Kohashi K, Nakagomi A, Saiki Y, Morisawa T, Kosugi M, Kusama Y et al. Effects of eicosapentaenoic acid on the levels of inflammatory markers, cardiac function and long-term prognosis in chronic heart failure patients with dyslipidemia. J Atheroscler Thromb 2014;21(7):712-729.

282. Kaul U, Sanghvi S, Bahl VK, Dev V, Wasir HS. Fish oil supplements for prevention of restenosis after coronary angioplasty. Int J Cardiol 1992;35(1):87-93.

283. Maki KC, Bays HE, Ballantyne CM, Underberg JA, Kastelein JJP, Johnson JB et al. A Head-to-Head Comparison of a Free Fatty Acid Formulation of Omega-3 Pentaenoic Acids Versus Icosapent Ethyl in Adults With Hypertriglyceridemia: The ENHANCE-IT Study. J Am Heart Assoc 2022;11(6):e024176.

284. Yuan F, Li H, Yang M, Chen W, Chen H, Xu H et al. Pharmacokinetics of Icosapent Ethyl: An Open-Label, Multiple Oral Dose, Parallel Design Study in Healthy Chinese Subjects. Clin Pharmacol Drug Dev 2023;12(1):6-13.

285. Barry AR, Dixon DL. Omega-3 fatty acids for the prevention of atherosclerotic cardiovascular disease. Pharmacotherapy 2021;41(12):1056-1065.

286. Kosmopoulos A, Bhatt DL, Meglis G, Verma R, Pan Y, Quan A et al. A randomized trial of icosapent ethyl in ambulatory patients with COVID-19. iScience 2021;24(9):103040.

287. Orringer CE, Jacobson TA, Maki KC. National Lipid Association Scientific Statement on the use of icosapent ethyl in statin-treated patients with elevated triglycerides and high or very-high ASCVD risk. J Clin Lipidol 2019;13(6):860-872.

288. Teramoto T, Shibata H, Suzaki Y, Matsui S, Uemura N, Tomiyama H et al. Discrepancy Between Fasting Flow-Mediated Dilation and Parameter of Lipids in Blood: A Randomized Exploratory Study of the Effect of Omega-3 Fatty Acid Ethyl Esters on Vascular Endothelial Function in Patients With Hyperlipidemia. Adv Ther 2020;37(5):2169-2183.

289. Dunbar RL, Gaudet D, Davidson M, Rensfeldt M, Yang H, Nilsson C et al. Omega-3 fatty acid exposure with a low-fat diet in patients with past hypertriglyceridemia-induced acute pancreatitis; an exploratory, randomized, open-label crossover study. Lipids Health Dis 2020;19(1):117.

290. Berger AA, Sherburne R, Urits I, Patel H, Eskander J. Icosapent Ethyl (Vascepa(R)) for the Treatment of Acute, Severe Pancreatitis. Cureus 2020;12(11):e11551.

291. Maki KC, Palacios OM, Buggia MA, Trivedi R, Dicklin MR, Maki CE. Effects of a Self-micro-emulsifying Delivery System Formulation Versus a Standard omega-3 Acid Ethyl Ester Product on the Bioavailability of Eicosapentaenoic Acid and Docosahexaenoic Acid: A Study in Healthy Men and Women in a Fasted State. Clin Ther 2018;40(12):2065-2076.

292. Back M, Hansson GK. Omega-3 fatty acids, cardiovascular risk, and the resolution of inflammation. FASEB J 2019;33(2):1536-1539.

293. Sezai A, Unosawa S, Taoka M, Osaka S, Obata K, Kanno S et al. Long-Term Comparison of Ethyl Icosapentate vs. Omega-3-Acid Ethyl in Patients With Cardiovascular Disease and Hypertriglyceridemia (DEFAT Trial). Circ J 2019;83(6):1368-1376.

294. Schott CK, Huang DT. omega-3 fatty acids, gamma-linolenic acid, and antioxidants: immunomodulators or inert dietary supplements? Crit Care 2012;16(6):325.

295. Martins JG. EPA but not DHA appears to be responsible for the efficacy of omega-3 long chain polyunsaturated fatty acid supplementation in depression: evidence from a meta-analysis of randomized controlled trials. J Am Coll Nutr 2009;28(5):525-542.

296. Parhofer KG, Chapman MJ, Nordestgaard BG. Efficacy and safety of icosapent ethyl in hypertriglyceridaemia: a recap. Eur Heart J Suppl 2020;22(Suppl J):J21-J33.

297. D'Almeida A, Carter JP, Anatol A, Prost C. Effects of a combination of evening primrose oil (gamma linolenic acid) and fish oil (eicosapentaenoic + docahexaenoic acid) versus magnesium, and versus placebo in preventing pre-eclampsia. Women Health 1992;19(2-3):117-131.

298. Deslypere JP. Influence of supplementation with N-3 fatty acids on different coronary risk factors in men--a placebo controlled study. Verh K Acad Geneeskd Belg 1992;54(3):189-216.

299. Bartoli GM, Palozza P, Marra G, Armelao F, Franceschelli P, Luberto C et al. n-3 PUFA and alpha-tocopherol control of tumor cell proliferation. Molecular aspects of medicine 1993;14(3):247-252.

300. Hoffman DR, Hughbanks-Wheaton DK, Pearson NS, Fish GE, Spencer R, Takacs A et al. Four-year placebo-controlled trial of docosahexaenoic acid in X-linked retinitis pigmentosa (DHAX trial): a randomized clinical trial. JAMA Ophthalmol 2014;132(7):866-873.

301. Nodari S, Triggiani M, Campia U, Manerba A, Milesi G, Cesana BM et al. n-3 polyunsaturated fatty acids in the prevention of atrial fibrillation recurrences after electrical cardioversion: a prospective, randomized study. Circulation 2011;124(10):1100-1106.

302. Kim CH, Han KA, Yu J, Lee SH, Jeon HK, Kim SH et al. Efficacy and Safety of Adding Omega-3 Fatty Acids in Statin-treated Patients with Residual Hypertriglyceridemia: ROMANTIC (Rosuvastatin-OMAcor iN residual hyperTrIglyCeridemia), a Randomized, Double-blind, and Placebo-controlled Trial. Clin Ther 2018;40(1):83-94.

303. Mosca L, Ballantyne CM, Bays HE, Guyton JR, Philip S, Doyle RT, Jr. et al. Usefulness of Icosapent Ethyl (Eicosapentaenoic Acid Ethyl Ester) in Women to Lower Triglyceride Levels (Results from the MARINE and ANCHOR Trials). Am J Cardiol 2017;119(3):397-403.

304. Manson JE, Cook NR, Lee IM, Christen W, Bassuk SS, Mora S et al. Vitamin D Supplements and Prevention of Cancer and Cardiovascular Disease. N Engl J Med 2019;380(1):33-44.

305. Investigators OT. Cardiovascular and Other Outcomes Postintervention With Insulin Glargine and Omega-3 Fatty Acids (ORIGINALE). Diabetes Care 2016;39(5):709-716.

306. Kromhout D, Geleijnse JM, de Goede J, Oude Griep LM, Mulder BJ, de Boer MJ et al. n-3 fatty acids, ventricular arrhythmia-related events, and fatal myocardial infarction in postmyocardial infarction patients with diabetes. Diabetes Care 2011;34(12):2515-2520.

307. Ghio S, Scelsi L, Latini R, Masson S, Eleuteri E, Palvarini M et al. Effects of n-3 polyunsaturated fatty acids and of rosuvastatin on left ventricular function in chronic heart failure: a substudy of GISSI-HF trial. Eur J Heart Fail 2010;12(12):1345-1353.

308. Finzi AA, Latini R, Barlera S, Rossi MG, Ruggeri A, Mezzani A et al. Effects of n-3 polyunsaturated fatty acids on malignant ventricular arrhythmias in patients with chronic heart failure and implantable cardioverter-defibrillators: A substudy of the Gruppo Italiano per lo Studio della Sopravvivenza nell'Insufficienza Cardiaca (GISSI-HF) trial. Am Heart J 2011;161(2):338-343 e331.

309. Aleksova A, Masson S, Maggioni AP, Lucci D, Fabbri G, Beretta L et al. n-3 polyunsaturated fatty acids and atrial fibrillation in patients with chronic heart failure: the GISSI-HF trial. Eur J Heart Fail 2013;15(11):1289-1295.

310. Angerer P, Kothny W, Stork S, von Schacky C. Effect of dietary supplementation with omega-3 fatty acids on progression of atherosclerosis in carotid arteries. Cardiovasc Res 2002;54(1):183-190.

311. Marchioli R, Levantesi G, Silletta MG, Barlera S, Bernardinangeli M, Carbonieri E et al. Effect of n-3 polyunsaturated fatty acids and rosuvastatin in patients with heart failure: results of the GISSI-HF trial. Expert Rev Cardiovasc Ther 2009;7(7):735-748.

312. Ishikawa Y, Yokoyama M, Saito Y, Matsuzaki M, Origasa H, Oikawa S et al. Preventive effects of eicosapentaenoic acid on coronary artery disease in patients with peripheral artery disease. Circ J 2010;74(7):1451-1457.

313. Marchioli R, Barzi F, Bomba E, Chieffo C, Di Gregorio D, Di Mascio R et al. Early protection against sudden death by n-3 polyunsaturated fatty acids after myocardial infarction: time-course analysis of the results of the Gruppo Italiano per lo Studio della Sopravvivenza nell'Infarto Miocardico (GISSI)-Prevenzione. Circulation 2002;105(16):1897-1903.

314. Bhatt DL, Miller M, Brinton EA, Jacobson TA, Steg PG, Ketchum SB et al. REDUCE-IT USA: Results From the 3146 Patients Randomized in the United States. Circulation 2020;141(5):367-375.

315. Verma S, Bhatt DL, Steg PG, Miller M, Brinton EA, Jacobson TA et al. Icosapent Ethyl Reduces Ischemic Events in Patients With a History of Previous Coronary Artery Bypass Grafting: REDUCE-IT CABG. Circulation 2021;144(23):1845-1855.

316. Singh N, Bhatt DL, Miller M, Steg PG, Brinton EA, Jacobson TA et al. Consistency of Benefit of Icosapent Ethyl by Background Statin Type in REDUCE-IT. J Am Coll Cardiol 2022;79(2):220-222.

317. Peterson BE, Bhatt DL, Steg PG, Miller M, Brinton EA, Jacobson TA et al. Treatment With Icosapent Ethyl to Reduce Ischemic Events in Patients With Prior Percutaneous Coronary Intervention: Insights From REDUCE-IT PCI. J Am Heart Assoc 2022;11(6):e022937.

318. Djousse L, Cook NR, Kim E, Walter J, Al-Ramady OT, Luttmann-Gibson H et al. Diabetes Mellitus, Race, and Effects of Omega-3 Fatty Acids on Incidence of Heart Failure Hospitalization. JACC Heart Fail 2022;10(4):227-234.

319. Selvaraj S, Bhatt DL, Steg PG, Miller M, Brinton EA, Jacobson TA et al. Impact of Icosapent Ethyl on Cardiovascular Risk Reduction in Patients With Heart Failure in REDUCE-IT. J Am Heart Assoc 2022;11(7):e024999.

320. Gaba P, Bhatt DL, Steg PG, Miller M, Brinton EA, Jacobson TA et al. Prevention of Cardiovascular Events and Mortality With Icosapent Ethyl in Patients With Prior Myocardial Infarction. J Am Coll Cardiol 2022;79(17):1660-1671.

321. Ridker PM, Rifai N, MacFadyen J, Glynn RJ, Jiao L, Steg PG et al. Effects of Randomized Treatment With Icosapent Ethyl and a Mineral Oil Comparator on Interleukin-1beta, Interleukin-6, C-Reactive Protein, Oxidized Low-Density Lipoprotein Cholesterol, Homocysteine, Lipoprotein(a), and Lipoprotein-Associated Phospholipase A2: A REDUCE-IT Biomarker Substudy. Circulation 2022;146(5):372-379.

322. Miller M, Bhatt DL, Steg PG, Brinton EA, Jacobson TA, Jiao L et al. Potential effects of icosapent ethyl on cardiovascular outcomes in cigarette smokers: REDUCE-IT smoking. Eur Heart J Cardiovasc Pharmacother 2023;9(2):129-137.

323. Nissen SE, Lincoff AM, Wolski K, Ballantyne CM, Kastelein JJP, Ridker PM et al. Association Between Achieved omega-3 Fatty Acid Levels and Major Adverse Cardiovascular Outcomes in Patients With High Cardiovascular Risk: A Secondary Analysis of the STRENGTH Trial. JAMA Cardiol 2021;6(8):1-8.

324. Jorgensen HS, Eide IA, Jenssen T, Asberg A, Bollerslev J, Godang K et al. Marine n-3 Polyunsaturated Fatty Acids and Bone Mineral Density in Kidney Transplant Recipients: A Randomized, Placebo-Controlled Trial. Nutrients 2021;13(7).

325. Picard F, Bhatt DL, Ducrocq G, Ohman EM, Goto S, Eagle KA et al. Generalizability of the REDUCE-IT trial and cardiovascular outcomes associated with hypertriglyceridemia among patients potentially eligible for icosapent ethyl therapy: An analysis of the REduction of Atherothrombosis for Continued Health (REACH) registry. Int J Cardiol 2021;340:96-104.

326. Camacho-Munoz D, Kiezel-Tsugunova M, Kiss O, Uddin M, Sunden M, Ryaboshapkina M et al. Omega-3 carboxylic acids and fenofibrate differentially alter plasma lipid mediators in patients with non-alcoholic fatty liver disease. FASEB J 2021;35(11):e21976.

327. Gaba P, Bhatt DL, Giugliano RP, Steg PG, Miller M, Brinton EA et al. Comparative Reductions in Investigator-Reported and Adjudicated Ischemic Events in REDUCE-IT. J Am Coll Cardiol 2021;78(15):1525-1537.

328. Majithia A, Bhatt DL, Friedman AN, Miller M, Steg PG, Brinton EA et al. Benefits of Icosapent Ethyl Across the Range of Kidney Function in Patients With Established Cardiovascular Disease or Diabetes: REDUCE-IT RENAL. Circulation 2021;144(22):1750-1759.

329. Ballantyne CM, Manku MS, Bays HE, Philip S, Granowitz C, Doyle RT, Jr. et al. Icosapent Ethyl Effects on Fatty Acid Profiles in Statin-Treated Patients With High Triglycerides: The Randomized, Placebo-controlled ANCHOR Study. Cardiol Ther 2019;8(1):79-90.

330. Baum SJ, Scholz KP. Rounding the corner on residual risk: Implications of REDUCE-IT for omega-3 polyunsaturated fatty acids treatment in secondary prevention of atherosclerotic cardiovascular disease. Clin Cardiol 2019;42(9):829-838.

331. Miller M, Ballantyne CM, Bays HE, Granowitz C, Doyle RT, Jr., Juliano RA et al. Effects of Icosapent Ethyl (Eicosapentaenoic Acid Ethyl Ester) on Atherogenic Lipid/Lipoprotein, Apolipoprotein, and Inflammatory Parameters in Patients With Elevated High-Sensitivity C-Reactive Protein (from the ANCHOR Study). Am J Cardiol 2019;124(5):696-701.

332. Vijayaraghavan K, Szerlip HM, Ballantyne CM, Bays HE, Philip S, Doyle RT, Jr. et al. Icosapent ethyl reduces atherogenic markers in high-risk statin-treated patients with stage 3 chronic kidney disease and high triglycerides. Postgrad Med 2019;131(6):390-396.

333. Hober A, Edfors F, Ryaboshapkina M, Malmqvist J, Rosengren L, Percy AJ et al. Absolute Quantification of Apolipoproteins Following Treatment with Omega-3 Carboxylic Acids and Fenofibrate Using a High Precision Stable Isotope-labeled Recombinant Protein Fragments Based SRM Assay. Mol Cell Proteomics 2019;18(12):2433-2446.

334. Lilleberg HS, Cichosz SL, Svensson M, Christensen JH, Fleischer J, Eide I et al. The Effect of Marine n-3 Polyunsaturated Fatty Acids on Heart Rate Variability in Renal Transplant Recipients: A Randomized Controlled Trial. Nutrients 2019;11(12).

335. Ferrieres J, Bataille V, Puymirat E, Schiele F, Simon T, Danchin N et al. Applicability of the REDUCE-IT trial to the FAST-MI registry. Are the results of randomized trials relevant in routine clinical practice? Clin Cardiol 2020;43(11):1260-1265.

336. Budoff MJ, Bhatt DL, Kinninger A, Lakshmanan S, Muhlestein JB, Le VT et al. Effect of icosapent ethyl on progression of coronary atherosclerosis in patients with elevated triglycerides on statin therapy: final results of the EVAPORATE trial. Eur Heart J 2020;41(40):3925-3932.

337. Peterson BE, Bhatt DL, Steg PG, Miller M, Brinton EA, Jacobson TA et al. Reduction in Revascularization With Icosapent Ethyl: Insights From REDUCE-IT Revascularization Analyses. Circulation 2021;143(1):33-44.

338. Nicholls SJ, Lincoff AM, Bash D, Ballantyne CM, Barter PJ, Davidson MH et al. Assessment of omega-3 carboxylic acids in statin-treated patients with high levels of triglycerides and low levels of high-density lipoprotein cholesterol: Rationale and design of the STRENGTH trial. Clin Cardiol 2018;41(10):1281-1288.
